# Selective serotonin reuptake inhibitors, and serotonin and norepinephrine reuptake inhibitors for anxiety, obsessive-compulsive, and stress disorders: A 3-level network meta-analysis

**DOI:** 10.1371/journal.pmed.1003664

**Published:** 2021-06-10

**Authors:** Natan Pereira Gosmann, Marianna de Abreu Costa, Marianna de Barros Jaeger, Luis Souza Motta, Júlia Frozi, Lucas Spanemberg, Gisele Gus Manfro, Pim Cuijpers, Daniel Samuel Pine, Giovanni Abrahão Salum

**Affiliations:** 1 Section of Negative Affect and Social Processes, Hospital de Clínicas de Porto Alegre, Federal University of Rio Grande do Sul, Porto Alegre, Brazil; 2 Anxiety Disorders Outpatient Program, Hospital de Clínicas de Porto Alegre, Federal University of Rio Grande do Sul, Porto Alegre, Brazil; 3 School of Medicine, Pontifícia Universidade Católica do Rio Grande do Sul, Porto Alegre, Brazil; 4 Department of Clinical, Neuro- and Developmental Psychology, Amsterdam Public Health Research Institute, Vrije Universiteit Amsterdam, Amsterdam, Netherlands; 5 Emotion and Development Branch, Section on Development and Affective Neuroscience, National Institute of Mental Health, Bethesda, Maryland, United States of America; Harvard Medical School, UNITED STATES

## Abstract

**Background:**

Anxiety, obsessive-compulsive, and stress-related disorders frequently co-occur, and patients often present symptoms of several domains. Treatment involves the use of selective serotonin reuptake inhibitors (SSRIs) and serotonin and norepinephrine reuptake inhibitors (SNRIs), but data on comparative efficacy and acceptability are lacking. We aimed to compare the efficacy of SSRIs, SNRIs, and placebo in multiple symptom domains in patients with these diagnoses over the lifespan through a 3-level network meta-analysis.

**Methods and findings:**

We searched for published and unpublished randomized controlled trials that aimed to assess the efficacy of SSRIs or SNRIs in participants (adults and children) with diagnosis of any anxiety, obsessive-compulsive, or stress-related disorder in MEDLINE, PsycINFO, Embase, and Cochrane Library from inception to 23 April 2015, with an update on 11 November 2020. We supplemented electronic database searches with manual searches for published and unpublished randomized controlled trials registered in publicly accessible clinical trial registries and pharmaceutical companies’ databases. No restriction was made regarding comorbidities with any other mental disorder, participants’ age and sex, blinding of participants and researchers, date of publication, or study language. The primary outcome was the aggregate measure of internalizing symptoms of these disorders. Secondary outcomes included specific symptom domains and treatment discontinuation rate. We estimated standardized mean differences (SMDs) with 3-level network meta-analysis with random slopes by study for medication and assessment instrument. Risk of bias appraisal was performed using the Cochrane Collaboration’s risk of bias tool. This study was registered in PROSPERO (CRD42017069090). We analyzed 469 outcome measures from 135 studies (*n* = 30,245). All medications were more effective than placebo for the aggregate measure of internalizing symptoms (SMD −0.56, 95% CI −0.62 to −0.51, *p* < 0.001), for all symptom domains, and in patients from all diagnostic categories. We also found significant results when restricting to the most used assessment instrument for each diagnosis; nevertheless, this restriction led to exclusion of 72.71% of outcome measures. Pairwise comparisons revealed only small differences between medications in efficacy and acceptability. Limitations include the moderate heterogeneity found in most outcomes and the moderate risk of bias identified in most of the trials.

**Conclusions:**

In this study, we observed that all SSRIs and SNRIs were effective for multiple symptom domains, and in patients from all included diagnostic categories. We found minimal differences between medications concerning efficacy and acceptability. This three-level network meta-analysis contributes to an ongoing discussion about the true benefit of antidepressants with robust evidence, considering the significantly larger quantity of data and higher statistical power when compared to previous studies. The 3-level approach allowed us to properly assess the efficacy of these medications on internalizing psychopathology, avoiding potential biases related to the exclusion of information due to distinct assessment instruments, and to explore the multilevel structure of transdiagnostic efficacy.

## Abstract 2

In a meta-analysis of randomized trials, Natan Pereira Gosmann and colleagues study efficacy of SSRIs and SNRIs for symptoms of anxiety, obsessive-compulsive, and stress-related disorders.

## Author summary

**Why was this study done?:**

- Studies assessing comorbidity in patients with anxiety, obsessive-compulsive, and stress-related disorders report rates above 50%, and patients often present symptoms of multiple symptom domains.
- The efficacy of selective serotonin reuptake inhibitors (SSRIs) and serotonin and norepinephrine reuptake inhibitors (SNRIs) on multiple mental health domains has not yet been studied by network meta-analysis in this field, to the best of our knowledge.
- Meta-analyses often restrain the statistical analysis to the most commonly used assessment instruments.

**What did the researchers do and find?:**

- We conducted a systematic review and 3-level network meta-analysis of 469 outcome measures, including all available measures of outcomes related to anxiety, obsessive-compulsive, and stress-related disorders.
- SSRIs and SNRIs presented small to moderate effect sizes for global improvement of mental health in participants from all diagnostic categories.
- We also found small to moderate effect sizes in our sensitivity analysis restricted to the most used assessment instruments; however, this restriction led to the exclusion of 72.71% of all outcome measures.

**What do these findings mean?:**

- Our results support previous findings related to the efficacy of SSRIs and SNRIs indicating that these medications are effective in multiple health domains.
- This study improved the evidence of the benefit of SSRIs and SNRIs for anxiety disorders. These results should guide psychiatrists, patients, clinicians, and policy makers on better evidence-based decisions for the initial treatment of these disorders.

## Introduction

Anxiety, obsessive-compulsive, and stress-related disorders are among the main causes of years lived with disability due to psychiatric disorders worldwide, being the leading cause in some countries [1,2]. While these conditions affect around 10% of the world’s population, only 10% of those affected receive appropriate treatment [3]. Costs associated with these disorders account for approximately 33% of mental-health-related expenditures, particularly those related to loss of productivity [4]. Therefore, offering appropriate evidenced-based treatment is crucial.

Controversy concerning antidepressants in the treatment of mood disorders [5,6] obscures vital questions for the treatment of other conditions, such as anxiety, obsessive-compulsive, and stress-related disorders. While selective serotonin reuptake inhibitors (SSRIs) and serotonin and norepinephrine reuptake inhibitors (SNRIs) are considered first-line pharmacological treatments [7], fewer large-scale quantitative reviews evaluate efficacy data for these conditions, as compared to mood disorders [8]. Accordingly, key questions remain unanswered. First, there is still debate on their efficacy and acceptability [9]. Second, across the many agents, sufficiently powered comparative efficacy and acceptability assessments are lacking [8]. Third, anxiety, obsessive-compulsive, and stress-related disorders often co-occur [10], but the efficacy of SSRIs and SNRIs for global improvement of transdiagnostic dimensions has not been studied [8]. Fourth, there is uncertainty about the most appropriate instruments to measure treatment gains due to the highly inconsistent and heterogeneous assessment landscape [11,12]. Therefore, including only studies restricted to specific scales, as previous network meta-analyses have commonly done [13,14], can lead to selective reporting, biased estimates, and exclusion of a great amount of the outcomes related to psychopathology. Lastly, effects of clinical and methodological moderators on the efficacy estimate of antidepressants need to be taken into account when investigating comparability across medications [6]. Hence, it is essential to assess the efficacy of these medications in multiple symptom domains, not restricting to any scale, and also to explore potential moderators of these estimates. Such data may inform patients, clinicians, and policy makers on the relative levels of efficacy in these many domains.

We aimed to evaluate the efficacy and acceptability of SSRIs, SNRIs, and placebo for internalizing symptoms of children and adults diagnosed with anxiety, obsessive-compulsive, or stress-related disorders, while also exploring the multilevel structure of efficacy in all symptom domains related to these diagnoses. We used data pooled through 3-level network meta-analysis and multiple 3-level meta-regression analyses accounting for clinical and methodological differences between studies.

## Methods

We report this study as recommended by the Preferred Reporting Items for Systematic Reviews and Meta-Analyses (PRISMA) extension statement for network meta-analysis (S1 Appendix) [15]. This study was prospectively registered in PROSPERO (CRD42017069090) on 12 June 2017, during data extraction, and updated in the register on 30 January 2018, to describe the stage of review and to include collaborators. Ethical approval was not required as this study synthesized data from already published studies.

### Inclusion criteria

We included randomized controlled trials (RCTs) assessing the efficacy of SSRIs, SNRIs, and placebo in participants with a primary diagnosis of any anxiety disorder, obsessive-compulsive disorder (OCD), or stress-related disorder according to standard diagnostic criteria (Feighner criteria, ICD-10, DSM-III, DSM-III-R, DSM-IV, DSM-IV-TR, DSM-5, and RDoC). No restriction was used regarding comorbidities with any other mental disorder (e.g., depression or bipolar disorder), participants’ age and sex, blinding of participants and researchers, date of publication, or study language. Studies had to compare any SSRI or SNRI with each other, with the same medication using distinct doses, or with placebo. We excluded trials with any kind of previous intervention (e.g., medication after psychotherapy), selection based on treatment resistance, or treatment arms with any combined intervention (e.g., medication and psychotherapy), given that we aimed to evaluate the efficacy of these antidepressants as monotherapy.

### Search strategy

We searched MEDLINE, PsycINFO, Embase, and Cochrane Library from inception to 23 April 2015, and updated the search on 11 November 2020, using keywords related to study design, interventions, and assessed disorders, defined after discussion with experts in this field (search terms are provided in S2 Appendix). We supplemented electronic database searches with manual searches for published and unpublished RCTs registered in ClinicalTrials.gov, ISRCTN registry, European Clinical Trials Database, Pan African Clinical Trials Registry, International Federation of Pharmaceutical Manufacturers & Associations, Australian New Zealand Clinical Trials Registry, Food and Drug Administration database, and pharmaceutical companies’ databases. Reference lists of included RCTs and relevant reviews were inspected, and experts were asked to indicate additional trials. We also contacted study authors to provide data of unpublished studies and to provide additional data related to incomplete reports of original papers, clarify inconsistencies, and report unpublished results.

### Data extraction and data synthesis

Four reviewers (MAC, MBJ, LSM, and JF), all psychiatrists, independently screened abstracts, assessed full-text articles, evaluated risk of bias, and extracted data, and a fifth reviewer (NPG) double-checked all data entries. Disagreements and inconsistencies were resolved by consensus of all review group members.

For trials with multiple publications, we included the most informative and complete study report. Any outcome measure of interest reported in only 1 of the reports was also extracted within the same trial data.

The primary outcome was the aggregate measure of internalizing symptoms (i.e., emotions and behaviors related to fear and response to stress). This measure is composed of any assessment of obsessive-compulsive, stress-related, or anxiety disorders that encompasses domains of generalized anxiety disorder, social anxiety disorder, panic disorder, agoraphobia, specific phobias, and separation anxiety disorder, as well as somatic symptoms and overall symptom severity. Subscale scores were included in the internalizing aggregate only if the total score of the higher factor was not reported within the same study. Secondary outcomes were treatment discontinuation rate due to any cause, discontinuation rate due to adverse events, and clusters of symptomatic scales classified by the authors into 7 groups (symptom domains) based on DSM-5 diagnostic criteria (generalized anxiety, social anxiety, somatic symptoms, panic, specific phobias, OCD, and post-traumatic stress disorder).

We included all baseline data and outcomes reported between 6 and 26 weeks of follow-up in the analysis. We considered outcome measures as close to 12 weeks as possible. If information at 12 weeks was not available, we used data from the time point closest to 12 weeks; if 2 time points were equidistant before and after 12 weeks, we used data from the later time point. Primary and secondary outcomes were defined before data analysis.

We used group-level data: Extracted information included primary and secondary outcomes, publication data, demographic data, inclusion and exclusion criteria of the study population, diagnostic system, intervention regime, control regime, sample comorbidities, items related to industry influence, data analysis method, discontinuation rates, response, and remission rates.

### Statistical analysis

We performed a 3-level network meta-analysis. We estimated efficacy as standardized mean difference (SMD), which was calculated by first estimating the standardized mean change (SMC), subtracting the initial score from the final score of any mental-health-related symptom to calculate change for each intervention group. After that, we subtracted the SMC of the placebo group from the SMC of the medication group [16], assuming a correlation between initial and final means of 0.25, based on previous reports of this measure concerning mental health assessments [17]. When not available, standard deviations (SDs) of baseline means were imputed using the mean of reported SDs of outcome measures evaluated with the same assessment instrument, as suggested by previous studies [18]. We interpreted SMDs of 0.2, 0.5, and 0.8 as small, moderate, and large effect size differences, respectively [19]. We present the multilevel structure of transdiagnostic efficacy with a circular bar plot, which indicates the effect of medications for each diagnosis and also the effect medications in specific symptom domains within each diagnosis. We report the estimated effect sizes for all included outcome measures with a caterpillar plot. This method presents the same structure as a forest plot, except that the estimates are ordered by their magnitude. This is preferable when there is a large number of estimates, focusing on the general pattern, given individual estimates are not fully discernible [20]. We assessed comparative efficacy using pairwise comparisons. Acceptability was measured by odds ratios (ORs) of treatment discontinuation due to any cause and treatment discontinuation due to adverse events. We estimated corresponding 95% confidence intervals (CIs) for all measures. Two-sided *p-*values less than 0.05 were considered statistically significant.

We conducted all meta-analysis and meta-regression models using 3-level models with random slopes by study for medication and assessment instrument (S3 Appendix) [21]. We estimated the between-study variance through τ^2^ estimates and heterogeneity through *I*^2^. Given that a placebo group could be used in multiple comparisons, the sample size of the placebo group was divided by the number of treatment comparisons [22]. We assessed network consistency using a local approach evaluating agreement between direct and indirect estimates of medication comparisons through the Bucher method [23]. Comparative acceptability was assessed using pairwise comparisons of the dropout rates of medications, using multilevel models with study as a random variable, given that the same trial may report rates of distinct medication groups. All analyses depict sample size (*n*), number of studies (*k*), and number of outcome measures (*o*). Analyses were performed using R (version 3.5.1), with package “metafor” [24].

### Assessment of bias

Risk of bias appraisal was performed using the Cochrane Collaboration’s risk of bias tool for RCTs [25]. We classified the risk of bias of studies as follows: low, if none of the domains in the instrument was rated as high risk of bias and 3 or fewer were rated as unclear risk; moderate, if one domain was rated as high risk of bias or none was rated as high risk of bias but 4 or more were rated as unclear risk; or high, for all other cases [26]. We assessed small study effects through funnel plots.

### Meta-regression analysis

Univariate and multiple meta-regression models considered the following variables: medication, comparator, equivalent dose (estimated using fluoxetine equivalents based on previous studies) [27], time to outcome measure, main diagnosis, sampling, sample age, publication year, benzodiazepine use, placebo lead-in, analysis method, and study funding. We classified study funding as academic, governmental or non-profit, industry, or unclear according to the funding source statement of the primary studies. We categorized all studies that did not explicitly report academic, governmental or non-profit, or industry funding sources or did not present any funding source statement as having unclear funding. Medication class was assessed only through univariate meta-regression. Since we evaluated each individual medication in the multiple meta-regression model, the inclusion of medication class would implicate multicollinearity. Also, we performed univariate meta-regressions with medication as moderator for each symptom domain. We performed all pairwise comparisons of medications for both efficacy and acceptability using the multiple meta-regression model with clinical and methodological moderators.

### Subgroup and sensitivity analyses

We performed a subgroup analysis for each included diagnosis using the multilevel aggregate measure. We also conducted a subgroup analysis restricting the analysis to the most used assessment instrument for each diagnosis, as commonly performed by previous network meta-analyses [13,14]. We conducted sensitivity analyses of efficacy estimates for the primary outcome considering imputation of baseline SD with the largest SD of assessment instrument, no baseline SD imputation, endpoint SMD as efficacy estimate, correlation between initial and final means of 0.5 and 0.7, only published trials, and only studies at low risk of bias. Moreover, for RCTs designed to evaluate patients diagnosed with OCD, we performed a sensitivity analysis excluding studies that included participants diagnosed with tic-related OCD, hoarding, repetitive behaviors of autism, or Tourette syndrome, given that these conditions are associated with lack of pharmacological responsiveness [28].

## Results

### Study characteristics

We screened 5,447 titles and abstracts and evaluated 420 full-text articles for inclusion (S4 Appendix). Of those, 23 (5.48%) full-text articles or complete reports were available only through direct contact with authors. We included 135 studies in the meta-analysis (124 published trials and 11 unpublished reports), which reported 469 outcome measures in 30,245 patients. Of those studies, we included 94 studies in the meta-regression analyses, due to incomplete report of moderators. All included studies were classified as double-blind. Generalized anxiety disorder was the main disorder assessed in 35 (25.93%) of the 135 trials, whereas social anxiety disorder was studied in 28 (20.74%), panic disorder in 25 (18.52%), OCD in 22 (16.30%), and post-traumatic stress disorder in 20 (14.81%); 5 (3.70%) trials were designed to evaluate more than 1 disorder. The mean age of participants in placebo groups was 35.69 years (SD, 10.59), compared with 36.10 years (SD, 9.50) in medication groups. Moreover, 117 (86.67%) trials were designed to assess adults, and 18 (13.33%) studies evaluated children and adolescents. Mean proportion of women was 53.80 (SD, 19.17) in the placebo group, compared with 55.06 (SD, 17.93) in medication groups. Of included studies, 23 (17.04%) were single-center trials. The median number of sites from multicenter trials was 22 (interquartile range, 11 to 46). Concerning diagnostic criteria, DSM-IV was used in 76 (56.30%) studies, DSM-III-R in 33 (24.44%), DSM-IV-TR in 14 (10.37%), and DSM-III in 3 (2.22%). Diagnostic criteria were not clear in 9 (6.67%) included studies (primary study information is provided in S5–S8 Appendices).

### Outcomes

**Fig 1:**
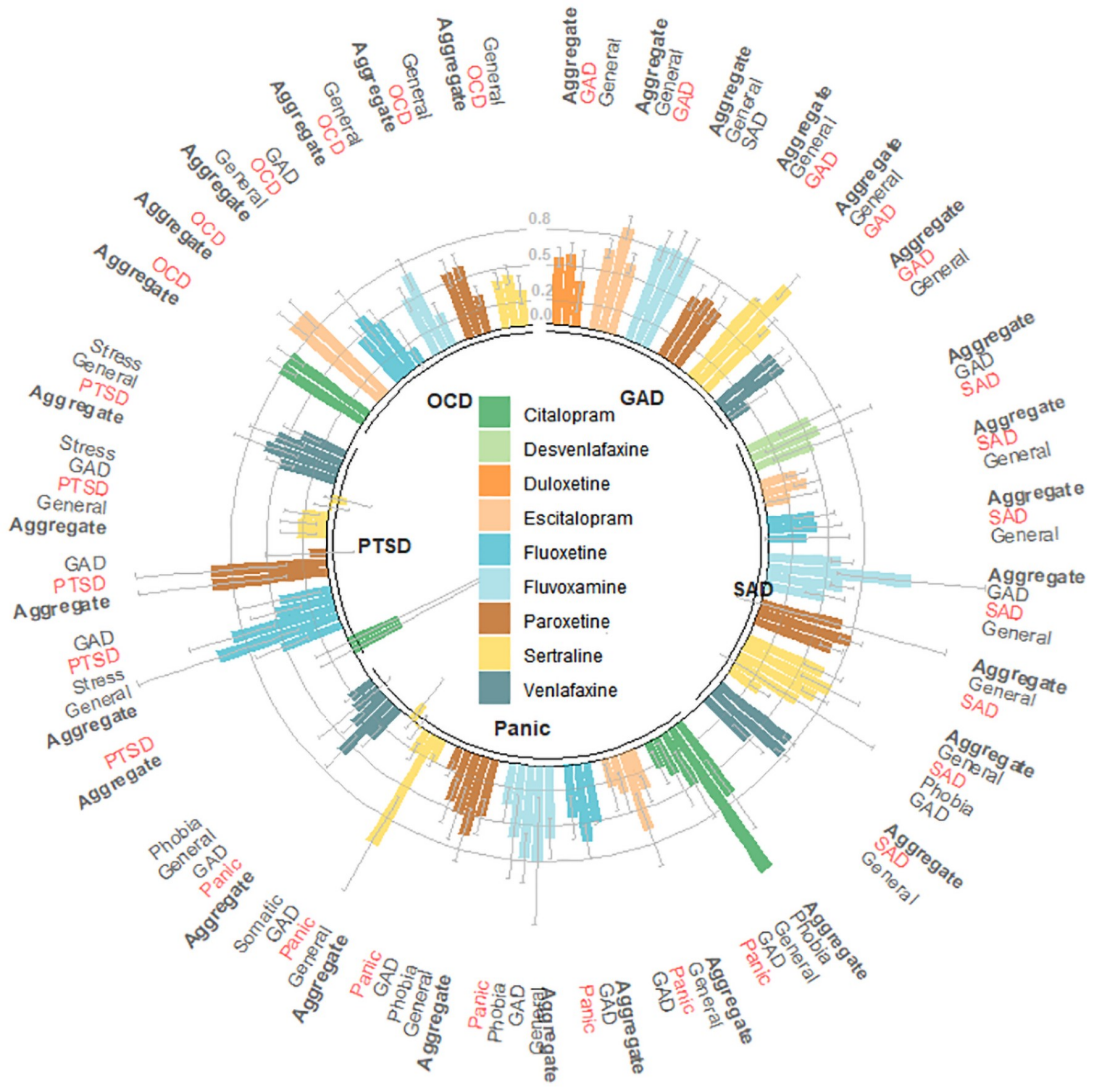
Standardized mean differences (SMDs) in the studied symptom domains within the 5 diagnoses. GAD, generalized anxiety disorder; OCD, obsessive-compulsive disorder; PTSD, post-traumatic stress disorder; SAD, social anxiety disorder. Patients’ diagnoses are presented in the center of the circular bar plot, and symptom domains are described outside. Effect sizes are presented as SMDs, and error bars represent estimated standard errors. SMDs related to the primary outcome (i.e., aggregate measure of the available symptom domains evaluated in patients within the same diagnosis) are highlighted in bold, and SMDs related to symptom domains that are concurrent with patients’ diagnosis are highlighted in red. Outcome measures classified as general represent scales designed to assess overall psychopathology.

**Fig 2:**
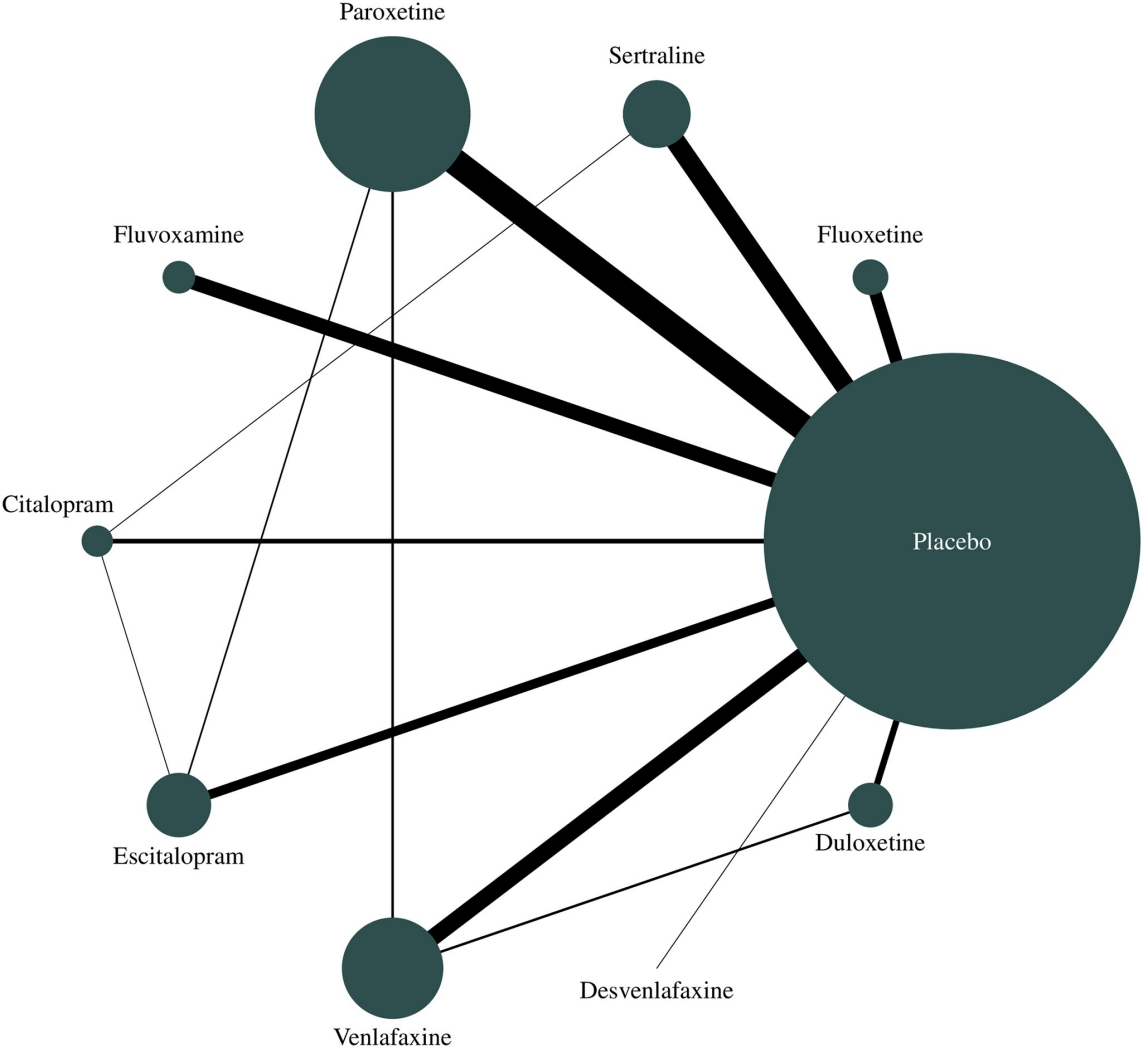
Network meta-analysis of available comparisons. Line width is proportional to the number of trials including every pair of treatments (direct comparisons). Circle size is proportional to the total number of participants randomly assigned to each treatment in the network.

**Fig 3:**
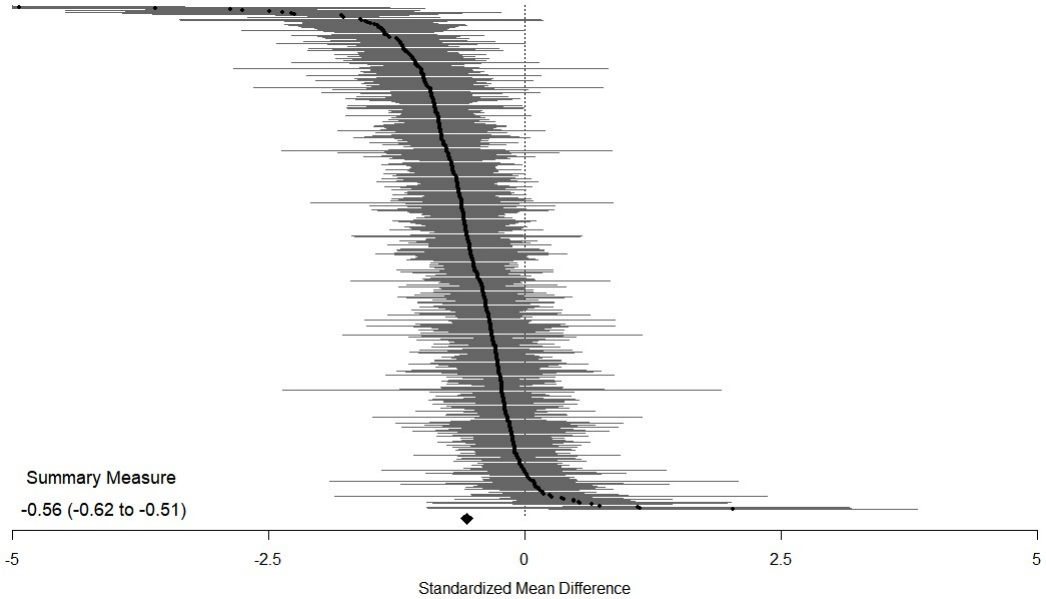
Caterpillar plot of all outcome measures included in the meta-analysis. Efficacy measured as standardized mean difference between medication and placebo for the primary outcome (aggregate measure of mental-health-related symptoms). Standardized mean differences less than 0 favor medication, and those greater than 0 favor placebo. Horizontal lines indicate 95% confidence intervals for each included outcome measure; the horizontal points of the diamond are the limits of the 95% confidence interval of the overall summary measure.

We found significant SMDs favoring medications over placebo for the pooled medication group (SMD −0.56, 95% CI −0.62 to −0.51, *p* < 0.001) and for all individual medications (Table 1), indicating moderate effect size on internalizing symptoms [26]. These differences reflect that SMCs from initial to final means in medication groups (SMC −1.70, 95% CI −1.83 to −1.57, *p* < 0.001) were higher than those found in placebo groups (SMC −1.11, 95% CI −1.22 to −1.00, *p* < 0.001) (S9 Appendix). We found moderate heterogeneity [22] for most outcomes. Figs 1 and 2 provide the multilevel structure of the study and the network diagram of direct comparisons, respectively. The caterpillar plot of all included outcome measures is presented in Fig 3.

**Table 1:** SMD between medication and placebo for the primary outcome (aggregate measure of mental-health-related symptoms) according to each medication class and each medication within the same class.

Medication	*o*/*k* (*n*)	Estimated SMD (95% CI)	SE	*p-*Value	τ^2^	Heterogeneity *I*^2^ (%)
**SSRIs and SNRIs**	469/135 (30,245)	−0.56 (−0.62 to −0.51)	0.03	<0.001	0.045	42.09
**SSRIs**	396/111 (22,146)	−0.57 (−0.64 to −0.50)	0.03	<0.001	0.039	37.68
Fluoxetine	64/16 (1,797)	−0.52 (−0.68 to −0.36)	0.08	<0.001	0.074	39.19
Sertraline	98/25 (4,071)	−0.43 (−0.57 to −0.29)	0.07	<0.001	0.091	58.83
Paroxetine	132/36 (8,790)	−0.60 (−0.72 to −0.49)	0.06	<0.001	0.091	64.05
Fluvoxamine	50/19 (2,276)	−0.68 (−0.88 to −0.49)	0.10	<0.001	0.162	68.55
Citalopram	19/6 (1,487)	−0.65 (−1.08 to −0.22)	0.22	0.003	0.196	66.24
Escitalopram	33/13 (3,725)	−0.61 (−0.76 to −0.46)	0.08	<0.001	0.048	46.47
**SNRIs**	73/28 (8,099)	−0.54 (−0.65 to −0.44)	0.05	<0.001	0.063	56.08
Venlafaxine	52/21 (5,621)	−0.55 (−0.68 to −0.41)	0.07	<0.001	0.094	65.58
Duloxetine	19/8 (2,418)	−0.56 (−0.71 to −0.41)	0.08	<0.001	0.021	31.44
Desvenlafaxine	2/1 (60)	−0.58 (−1.14 to −0.03)	0.28	0.04	—	—

*k*, number of studies; *n*, sample size; *o*, number of outcomes; SE, standard error; SMD, standardized mean difference; SNRI, serotonin and norepinephrine reuptake inhibitor; SSRI, selective serotonin reuptake inhibitor.

**Fig 4:**
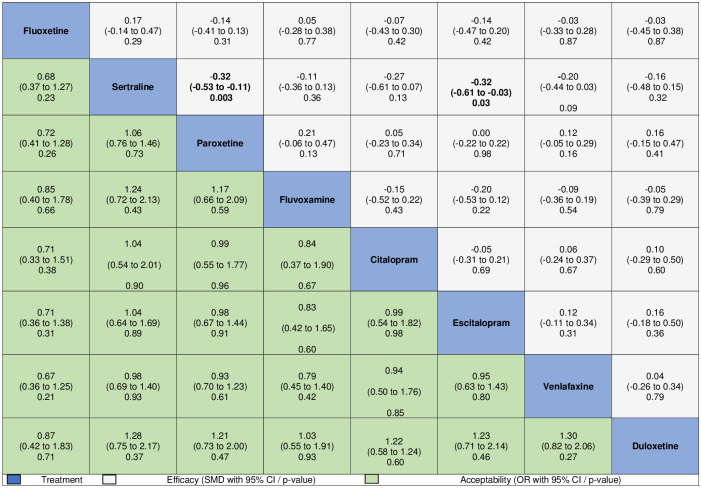
Comparisons of efficacy and discontinuation rates of all selective serotonin reuptake inhibitors and serotonin and norepinephrine reuptake inhibitors, considering 3-level multiple meta-regression models. Comparisons between treatments should be read from left to right, and the estimate is in the cell in common between the column-defining treatment and the row-defining treatment. For efficacy, standardized mean differences (SMDs) below 0 favor the column-defining treatment. For safety, odds ratio (ORs) above 1 favor the column-defining treatment.

**Fig 5:**
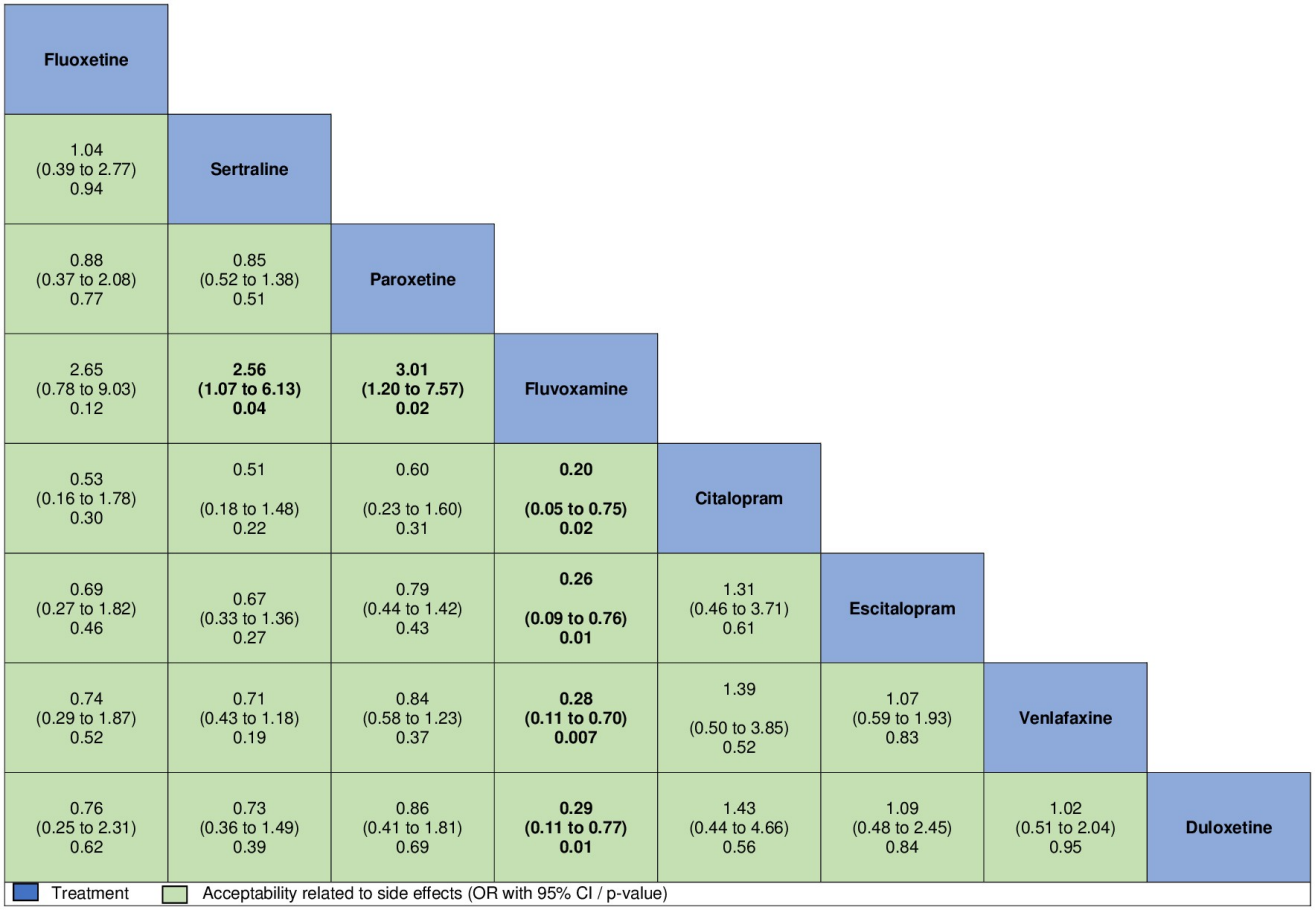
Comparisons of discontinuation rates due to adverse events of all selective serotonin reuptake inhibitors and serotonin and norepinephrine reuptake inhibitors, considering 3-level multiple meta-regression models. Comparisons between treatments should be read from left to right, and the estimate is in the cell in common between the column-defining treatment and the row-defining treatment. Odds ratios (ORs) above 1 favor the column-defining treatment.

Medication type did not significantly moderate treatment response. However, pairwise efficacy comparisons indicated that, compared to sertraline, both paroxetine (SMD −0.32, 95% CI −0.53 to −0.11, *p* = 0.003) and escitalopram (SMD −0.32, 95% CI −0.61 to −0.03, *p* = 0.03) were significantly more effective for the aggregate measure of internalizing symptoms, with no further significant differences between all other medications. Direct estimates were consistent with these findings (S10 Appendix). We also performed pairwise comparisons assessing acceptability differences among medications. No differences among medications were found for discontinuation rate due to any cause (Fig 4). Nevertheless, in comparison with all other medications except fluoxetine, fluvoxamine was associated with a higher rate of discontinuation due to adverse events (Fig 5).

All symptom domains related to anxiety, OCD, or stress disorders exhibited a favorable SMD in medication–placebo comparisons that could be classified as small to moderate (Table 2) [19]. Analyses also considered univariate meta-regressions for each included symptomatic domain with medication as moderator. Fluvoxamine was more effective for generalized anxiety disorder symptoms than fluoxetine (SMD −0.44, 95% CI −0.86 to −0.02, *p* = 0.04). For social anxiety disorder, panic disorder, post-traumatic stress disorder, and OCD symptoms, no significant differences between medications were found (S11 Appendix).

**Table 2:** SMD between medication (selective serotonin reuptake inhibitor or serotonin and norepinephrine reuptake inhibitor) and placebo for each symptom domain in the included studies.

Symptom domain	*o*/*k* (*n*)	Estimated SMD (95% CI)	SE	*p-*Value	τ^2^	Heterogeneity *I*^2^ (%)
GAD	128/68 (16,495)	−0.55 (−0.64 to −0.46)	0.05	<0.001	0.078	56.83
Social anxiety disorder	57/28 (6,668)	−0.67 (−0.76 to −0.58)	0.05	<0.001	0.005	9.78
Panic disorder	55/17 (4,040)	−0.30 (−0.37 to −0.23)	0.04	<0.001	0.034	36.16
Specific phobias	23/11 (2,651)	−0.51 (−0.78 to −0.25)	0.13	<0.001	0.008	16.49
PTSD	49/20 (2,907)	−0.42 (−0.67 to −0.17)	0.13	0.001	0.206	71.04
OCD	63/22 (3,835)	−0.59 (−0.70 to −0.48)	0.06	<0.001	0.001	1.34

GAD, generalized anxiety disorder; *k*, number of studies; *n*, sample size; *o*, number of outcomes; OCD, obsessive-compulsive disorder; PTSD, post-traumatic stress disorder; SE, standard error; SMD, standardized mean difference.

### Univariate and multiple meta-regression analyses

We performed univariate (S12 Appendix) and multiple (S13 Appendix) 3-level meta-regression analyses to investigate potential sources of heterogeneity in medication–placebo comparisons for the primary outcome. The multiple meta-regression model indicated higher efficacy for the aggregate measure of internalizing symptoms for 4 factors: (a) older relative to newer studies; (b) studies with outcome assessments at weeks 12 to 14, compared to those evaluating outcomes between weeks 6 to 8 and 9 to 11; (c) participants diagnosed with generalized anxiety disorder, compared to other diagnoses; and (d) studies funded by academic institutions, compared to all other sources of funding.

### Risk of bias assessment

Overall, 32 (23.70%) trials were rated as having high risk of bias, 65 (48.15%) as moderate, and 38 (28.15%) as low (S14 and S15 Appendices). Visual inspection of funnel plots did not suggest that small studies gave different results from larger studies in medication–placebo comparisons (S16–S22 Appendices).

### Subgroup and sensitivity analyses

We found significant results for efficacy on the aggregate measure of internalizing symptoms for all groups of standardized diagnosis of participants (Table 3), ranging from a SMD of −0.41 (95% CI −0.65 to −0.18, *p* < 0.001) for post-traumatic stress disorder to a SMD of −0.65 (95% CI −0.74 to −0.56, *p* < 0.001) for social anxiety disorder. Only 1 study assessed participants with primary diagnosis of specific phobia, so it was not included in the analysis stratified by mental disorder, given that it would not represent a pooled 3-level estimate. We also found significant results when restricting analysis to the most used assessment instrument for each diagnosis for all groups of standardized diagnosis of participants (Table 4), ranging from a SMD of −0.13 (95% CI −0.24 to −0.02, *p* = 0.02) for panic disorder to a SMD of −0.64 (95% CI −0.75 to −0.53, *p* < 0.001) for social anxiety disorder; however, this restriction led to the exclusion of 341 (72.71%) available outcome measures. Concerning sensitivity analyses, all efficacy estimates remained within the 95% CI of the main analysis (Table 5). In RCTs designed to assess OCD, we found SMDs of −0.53 (95% CI −0.71 to −0.35, *p* < 0.001) and −0.53 (95% CI −0.66 to −0.41, *p* < 0.001) for RCTs that included and excluded patients diagnosed with tic-related OCD, hoarding, repetitive behaviors of autism, or Tourette syndrome, respectively.

**Table 3:** SMD between medication and placebo for the primary outcome (aggregate measure of mental-health-related symptoms) according to standardized diagnosis in the participants of included studies.

DSM-5 diagnosis	*o*/*k* (*n*)	Estimated SMD (95% CI)	SE	*p-*Value	τ^2^	Heterogeneity *I*^2^ (%)
GAD	92/35 (10,564)	−0.64 (−0.73 to −0.55)	0.05	<0.001	0.044	45.25
Social anxiety disorder	75/28 (6,454)	−0.65 (−0.74 to −0.56)	0.05	<0.001	0.025	32.97
Panic disorder	134/25 (5,995)	−0.43 (−0.55 to −0.31)	0.06	<0.001	0.101	64.30
PTSD	69/20 (2,907)	−0.41 (−0.65 to −0.18)	0.12	<0.001	0.195	71.62
OCD	91/22 (3,849)	−0.53 (−0.64 to −0.42)	0.05	<0.001	0.003	2.99

GAD, generalized anxiety disorder; *k*, number of studies; *n*, sample size; *o*, number of outcomes; OCD, obsessive-compulsive disorder; PTSD, post-traumatic stress disorder; SE, standard error; SMD, standardized mean difference.

**Table 4:** SMD between medication and placebo for the most used assessment instrument according to standardized diagnosis of participants.

DSM-5 diagnosis and instrument	*o*/*k* (*n*)	Estimated SMD (95% CI)	SE	*p-*Value	τ^2^	Number of excluded outcomes (%)	Number of excluded studies (%)	Number of excluded participants (%)	Heterogeneity *I*^2^ (%)
Aggregate	128/93 (23,330)	−0.56 (−0.63 to −0.49)	0.04	<0.001	0.045	341 (72.71)	42 (31.11)	6,915 (22.86)	40.00
GAD—HAM-A	42/32 (9,962)	−0.61 (−0.72 to −0.50)	0.05	<0.001	0.035	50 (54.35)	3 (8.57)	602 (5.70)	61.51
Social anxiety disorder—LSAS	28/22 (5,433)	−0.64 (−0.75 to −0.53)	0.06	<0.001	0.023	47 (62.67)	6 (21.43)	1,021 (15.82)	30.09
Panic disorder—PAAS panic attacks/week	15/9 (2,265)	−0.13 (−0.24 to −0.02)	0.06	0.02	0.00	119 (88.81)	16 (64.0)	3,730 (62.22)	0.00
PTSD—CAPS	18/15 (2,570)	−0.51 (−0.71 to −0.31)	0.10	<0.001	0.044	51 (73.91)	5 (25.0)	337 (11.59)	29.46
OCD—YBOCS	25/15 (3,100)	−0.63 (−0.82 to −0.45)	0.09	<0.001	0.062	66 (72.53)	7 (31.82)	749 (19.46)	39.30

CAPS, Clinician-Administered PTSD Scale; GAD, generalized anxiety disorder; HAM-A, Hamilton Anxiety Rating Scale; *k*, number of studies; LSAS, Liebowitz Social Anxiety Scale; *n*, sample size; *o*, number of outcomes; OCD, obsessive-compulsive disorder; PAAS, Panic and Anticipatory Anxiety Scale; PTSD, post-traumatic stress disorder; SE, standard error; SMD, standardized mean difference; YBOCS, Yale–Brown Obsessive Compulsive Scale.

**Table 5:** Sensitivity analysis of each method of measure of association estimate between medication and placebo for the primary outcome (aggregate measure of mental-health-related symptoms).

Method	*o*/*k* (*n*)	Estimated measure (95% CI)	SE	*p-*Value	τ^2^	Heterogeneity *I*^2^ (%)
Only published	432/124 (28,196)	−0.58 (−0.64 to −0.52)	0.03	<0.001	0.061	49.64
SD imputation (maximum SD)	469/135 (30,245)	−0.52 (−0.57 to −0.46)	0.03	<0.001	0.074	55.50
No SD imputation	425/121 (27,228)	−0.55 (−0.61 to −0.49)	0.03	<0.001	0.078	55.51
Correlation of 0.5	469/135 (30,245)	−0.56 (−0.62 to −0.50)	0.03	<0.001	0.079	63.02
Correlation of 0.7	469/135 (30,245)	−0.56 (−0.61 to −0.50)	0.03	<0.001	0.068	66.75
Excluding outliers	462/132 (29,955)	−0.55 (−0.60 to −0.49)	0.03	<0.001	0.063	51.19
Endpoint standardized mean difference	185/53 (8,256)	−0.43 (−0.50 to −0.36)	0.04	<0.001	0.047	58.20
Only studies at low risk of bias	179/38 (9,291)	−0.56 (−0.67 to −0.45)	0.06	<0.001	0.100	60.19

*k*, number of studies; *n*, sample size; *o*, number of outcomes; SE, standard error.

## Discussion

In this study, we aimed to assess the efficacy and acceptability of SSRIs, SNRIs, and placebo for internalizing symptoms of children and adults diagnosed with anxiety, obsessive-compulsive, or stress-related disorders, accounting for clinical and methodological differences. Our results revealed higher efficacy of medications than placebo on the aggregate measure of internalizing symptoms. Effect sizes were small to moderate in overall psychopathology for all considered diagnoses and in all symptom domains. We also found significant results when restricting the analysis to the most used assessment instrument in each diagnosis; however, this restriction led to the exclusion of 72.71% of all available outcome measures. Moreover, estimates of efficacy were moderated by patient diagnosis, treatment duration, study funding, and study year of publication. Finally, concerning pairwise comparisons, we found small between-medication differences for paroxetine and escitalopram when compared to sertraline, considering efficacy. When evaluating acceptability through discontinuation rate due to any cause, no differences among medications were found; nevertheless, fluvoxamine was associated with a higher rate of discontinuation due to adverse events than all other medications, except fluoxetine.

To our knowledge, this is the first meta-analysis using a 3-level approach to evaluate the efficacy of antidepressants on multiple mental health domains of patients diagnosed with anxiety, obsessive-compulsive, or stress-related disorders [8]. All included SSRIs and SNRIs showed greater reduction in overall psychopathology than placebo, with effect sizes comparable to those of other interventions in medicine [29]. Combined with data on major depression [30], this should address concerns on the benefit of SSRIs and SNRIs in global mental health, given that one of the main criticisms about previous studies is that they did not account for multiple domains of emotional distress [5]. Moreover, our findings provide support for transdiagnostic systems of psychopathology, which emphasize that psychosocial impairment is better explained and predicted by transdiagnostic dimensions than traditional diagnoses [31,32]. Studies assessing comorbidity in patients with anxiety, obsessive-compulsive, and stress-related disorders report rates above 50% [10]. Standard network meta-analyses are designed to evaluate symptom domains separately [14], which might not represent most patients in clinical settings; thus, current evidence may be potentially misleading. This suggests the need to evaluate efficacy of treatments in multiple symptom domains, given that patients seek help for overall improvement in symptoms and functioning rather than improvements in specific symptom domains. In addition, there is no gold standard for assessing symptom severity for anxiety disorders, and standard network meta-analyses often restrict outcome measures to specific scales [13,14]. We also found small to moderate effect sizes when restricting the analysis to the most used assessment instrument in each diagnosis in our sensitivity analysis; nevertheless, this restriction led to the exclusion of 72.71% of all available outcome measures. This may indicate that a great amount of the literature is not included in previous studies, which significantly constraints current evidence and limits power. Hence, multiple-endpoint design also addresses low item overlap between assessment instruments, ranging from 37% similarity for anxiety scales to 45% for post-traumatic stress disorder, and concerns about biases inherent to each scale, given the inconsistent and highly heterogeneous current assessment landscape [11,12].

Publication of network meta-analyses in the psychiatry field is significantly increasing [8] as these analyses have been recognized as the highest level of evidence in treatment guidelines [33]. Nonetheless, unlike major depression and other narrowly defined psychiatric disorders, which allow a more “unidimensional” construct assessment, anxiety disorders are a group of highly correlated emotional disorders that require a distinct approach. The 3-level design addresses this important issue, at the same time allowing us to combine direct and indirect information in a network [34–36]. Although 3-level network meta-analyses, like standard meta-analyses, are susceptible to the quality of the primary studies, 3-level network meta-analyses may represent a significant methodological advancement to be used in this research field.

Cross-medication comparisons revealed lower efficacy of sertraline compared to paroxetine and escitalopram, and lower acceptability of fluvoxamine related to adverse events compared to all other medications, except fluoxetine. These findings could inform evidence-based medication choices. Nonetheless, these results should be interpreted cautiously, since differences concerning efficacy indicated small effect sizes, and statistically significant findings related to acceptability presented notably wide confidence intervals. Therefore, clinicians should also consider factors beyond efficacy and acceptability, such as patient’s prior experience with medication, the physician’s own experience, and potential budgetary constraints [37].

The most comprehensive network meta-analysis on medications for anxiety disorders before this analysis [14], which assessed only generalized anxiety disorder, found results consistent with our findings, indicating that SSRIs and SNRIs are effective for generalized anxiety disorder and that there are no significant differences among medications. Nevertheless, this previous work assessed only 89 outcome measures, which represents 18.98% of the 469 evaluated in our study. This significant difference is partially related to the exclusion of comorbidities. Given that anxiety disorders often co-occur, we understand that the inclusion of distinct disorders is a crucial aspect of this field. Bandelow and colleagues [38] also assessed the efficacy of antidepressants for anxiety disorders, including not only generalized anxiety disorder but also social anxiety disorder and panic disorder. Bandelow and colleagues’ work represents the largest meta-analysis in this field, evaluating 206 treatment arms related to the efficacy of medications. Without using a network meta-analysis approach, this work reported effect sizes of 2.09 for SSRIs and 2.25 for SNRIs and indicated substantial differences between medications, with effect sizes ranging from 1.06 for citalopram to 2.75 for escitalopram. These conflicting findings may be due to the use of pre–post effect sizes, which estimate the improvement within one group and not the difference between the intervention and the placebo group. This suggests a large variation in placebo response rates in trials assessing different medications for these disorders. Despite being commonly used, pre–post effect estimates have been criticized in the literature [17], given that it is impossible to disentangle which proportion of the effect size is caused by the intervention and which by other processes, such as natural recovery or the expectations of the patients.

Anxiety, obsessive-compulsive, and stress-related disorders often co-occur; given this, 2 previous meta-analyses have explored the benefit of antidepressants for these conditions. Roest and colleagues mainly focused on premarketing trials and found an overall effect size of 0.38, including 49 studies [9]. Sugarman and colleagues reported similar results, indicating an effect size of 0.34 based on 56 outcome measures [39]. These discrepancies compared to our findings and to our number of outcome measures reflect a major difference related to our 3-level approach. All previous meta-analyses included only 1 outcome measure for study, while we included all available outcome measures for each study. Since there is a dependency between effect sizes of the same study, we took these dependencies into account with the 3-level meta-analytical model [21], including assessment instrument as a random variable, and also using a network meta-analysis approach, including medication as a random variable. Moreover, these 2 previous studies restricted assessment instruments to the scales most commonly used in each diagnosis, which can lead to biased estimates and not account for co-occurring symptoms of distinct domains. Furthermore, our larger quantity of data allowed us to explore different potential moderators, given the higher statistical power.

We found no age group moderation effect, indicating that SSRIs and SNRIs are also effective for anxiety symptoms in younger individuals. These findings contrast with previous evidence on the efficacy of antidepressants for depressive symptoms indicating that children and adolescents do not present good response to treatments with SSRIs or SNRIs compared with adults [13]. Given that the temporal relationship of comorbidity suggests that the onset of anxiety disorders often occurs earlier, aiming to reduce psychopathology and morbidity before the onset of depression may be an important prevention strategy in clinical practice to be further investigated. Also, children and adolescents do not respond as well to psychotherapy as adults do [40], so pharmacological interventions may be of great importance.

### Strengths and limitations of the study

This study has some major strengths. To the best of our knowledge, this is the first 3-level network meta-analysis in the field of psychiatry and the largest meta-analysis to date to evaluate the efficacy of antidepressants on mental health symptoms of patients diagnosed with anxiety, obsessive-compulsive, or stress-related disorders, due to full inclusion of all available outcome measures in this field, and an extensive search for both published and unpublished trials, with no restriction regarding participant age, date of publication, or study language. This approach allows a well-powered comparison of efficacy and acceptability among these medications, exploring the multilevel structure of efficacy, avoiding exclusion of a great amount of available outcome measures, and avoiding biases related to specific symptoms or inherent to assessment instruments. Moreover, we extracted detailed clinical and methodological information for each included study, exploring potential moderators of efficacy estimates.

Nevertheless, our study has some limitations. First, the risk of bias assessment indicated some sources of potential bias, possibly restricting interpretation of the results; however, our sensitivity analysis of trials with low risk of bias produced estimates consistent with our main findings. Second, visual inspection of funnel plots indicated that small studies present different results from larger studies in some symptom domains, which may suggest a publication bias in this research field. Through an extensive search for both published and unpublished trials, we aimed to reduce the impact of this issue. Despite our larger quantity of data, and resulting greater statistical power, compared to other meta-analyses, our results should be interpreted cautiously. Third, standard deviations of baseline measures are not informed in all included studies and correlation between baseline and endpoint means were sparsely reported and, for this reason, were imputed or assumed. Nonetheless, the imputation method followed previous recommendations for meta-analyses [18], and the assumed correlation was based on previous reports concerning mental health [17]. Lastly, we identified moderate heterogeneity in our data analysis, as expected in meta-analyses with a 3-level design and with a large number of studies [41]. Accordingly, we explored and identified potential sources of heterogeneity through meta-regression and sensitivity analysis.

### Conclusions

To our knowledge, our 3-level network meta-analysis represents the most comprehensive review of available evidence to date regarding the efficacy of SSRIs and SNRIs for the treatment of anxiety, obsessive-compulsive, and stress-related disorders, considering not only specific domains but all assessments of internalizing symptoms related to these disorders. Our findings, estimated using a 3-level approach, improve the evidence for the benefit of SSRIs and SNRIs for anxiety disorders, given that previous meta-analyses were restricted to specific scales or specific symptom domains, which reduces statistical power and does not reflect clinical practice. This method allowed us to properly estimate the efficacy of these medications on overall psychopathology, avoiding potential biases related to assessment instruments, and also to explore the multilevel structure of transdiagnostic efficacy. Our study might contribute to guiding psychiatrists, patients, clinicians, and policy makers on better evidence-based decisions for the initial treatment of these disorders.

## Supporting information

S1 Appendix PRISMA network meta-analyses checklist. (DOCX)

S2 Appendix Search terms. (DOCX)

S3 Appendix Three-level model description. (DOCX)

S4 Appendix Flowchart of included and excluded studies. (DOCX)

S5 Appendix Study general information. (DOCX)

S6 Appendix Study demographic information. (DOCX)

S7 Appendix Intervention information. (DOCX)

S8 Appendix Outcome assessment information. (DOCX)

S9 Appendix Standardized mean change from baseline to endpoint for placebo, medication class, and medications within the same class for the primary outcome (aggregate measure of mental-health-related symptoms) retrieved from the included studies. (DOCX)

S10 Appendix Direct and indirect standardized mean differences for available head-to-head medication comparisons for the primary outcome (aggregate measure of mental-health-related symptoms). (DOCX)

S11 Appendix Univariate meta-regression according to medication versus placebo for each symptom domain in the included studies. (DOCX)

S12 Appendix Univariate meta-regressions for the primary outcome (aggregate measure of mental-health-related symptoms) comparing medication versus placebo. (DOCX)

S13 Appendix Multiple meta-regression for the primary outcome (aggregate measure of mental-health-related symptoms) comparing medication versus placebo. (DOCX)

S14 Appendix Risk of bias summary. (DOCX)

S15 Appendix Risk of bias in included studies. (DOCX)

S16 Appendix Funnel plot for all internalizing symptoms. (DOCX)

S17 Appendix Funnel plot for the generalized anxiety disorder domain. (DOCX)

S18 Appendix Funnel plot for the panic disorder domain. (DOCX)

S19 Appendix Funnel plot for the social anxiety disorder domain. (DOCX)

S20 Appendix Funnel plot for the specific phobia domain. (DOCX)

S21 Appendix Funnel plot for the obsessive-compulsive disorder domain. (DOCX)

S22 Appendix Funnel plot for the post-traumatic stress disorder domain. (DOCX)

## Abbreviations

OCD: obsessive-compulsive disorder
OR: odds ratio
RCT: randomized controlled trial
SD: standard deviation
SMC: standardized mean change
SMD: standardized mean difference
SNRI: serotonin and norepinephrine reuptake inhibitor
SSRI: selective serotonin reuptake inhibitor
