# Supplementary material for: Selective serotonin reuptake inhibitors, and serotonin and norepinephrine reuptake inhibitors for anxiety, obsessive-compulsive, and stress disorders: A 3-level network meta-analysis
Source: PLoS Med. 2021 Jun 10;18(6):e1003664. doi: 10.1371/journal.pmed.1003664 (PMC8224914; doi:10.1371/journal.pmed.1003664)
Supplement: S2 Appendix — (DOCX) [file pmed.1003664.s002.docx]

**S2 Appendix. Search terms**

(anxi* OR GAD OR phobi* OR “social anxiety” OR panic* OR obsessi* OR compulsi* OR traumatic* OR posttrauma* OR post-trauma* OR "post trauma*” OR "combat disorder*" OR "stress disorder*" OR OCD OR ptsd) AND ("selective serotonin reuptake" OR “selective serotonin re-uptake inhibitors” OR “serotonin-specific reuptake inhibitors” OR ssri OR fluoxetine OR fluvoxamine OR sertraline OR paroxetine OR citalopram OR escitalopram OR dapoxetine OR “serotonin-norepinephrine reuptake" OR SNRI* OR venlafaxine OR desvenlafaxine OR duloxetine OR milnacipran OR Levomilnacipran) AND ((randomized controlled trial[Publication Type] OR (randomized[Title/Abstract] OR randomised[Title/Abstract]) AND controlled[Title/Abstract] AND trial[Title/Abstract]) OR (meta-analysis OR metaanalysis OR "systematic review" OR metaanalyses OR meta-analyses OR "systematic-review"))**/**
