# Supplementary material for: Selective serotonin reuptake inhibitors, and serotonin and norepinephrine reuptake inhibitors for anxiety, obsessive-compulsive, and stress disorders: A 3-level network meta-analysis
Source: PLoS Med. 2021 Jun 10;18(6):e1003664. doi: 10.1371/journal.pmed.1003664 (PMC8224914; doi:10.1371/journal.pmed.1003664)
Supplement: S3 Appendix — (DOCX) [file pmed.1003664.s003.docx]

**S3 Appendix. Three-level model description**

model1<-rma.mv(yi=yi,

V=V,

data=data,

random = list(~instrument|study, ~medication|study),

slab=paste(author, instrument, sep=", "))
