# Supplementary material for: Selective serotonin reuptake inhibitors, and serotonin and norepinephrine reuptake inhibitors for anxiety, obsessive-compulsive, and stress disorders: A 3-level network meta-analysis
Source: PLoS Med. 2021 Jun 10;18(6):e1003664. doi: 10.1371/journal.pmed.1003664 (PMC8224914; doi:10.1371/journal.pmed.1003664)
Supplement: S4 Appendix — (DOCX) [file pmed.1003664.s004.docx]

**S4 Appendix. Flowchart of included and excluded studies**

Records screened
(n =5447)

Records after duplicates removed
(n =5447)

Studies included in quantitative synthesis (meta-analysis)
(n =135)

Articles excluded due to incomplete report of any moderator
(n =41)

Studies included in meta-regression analysis
(n =94)

Full-text articles excluded:

(n=285)

Previously reported data (n=86)

No data of interest reported (n=68)

Impossibility for extraction (n=39)

Insufficient data for meta-analysis (n=37)

Participants without anxiety disorders (n=20)

Not randomized (n=8)

Other (n=27)

Records excluded
(n = 5027)

## Eligibility

## Screening

## Identification

## Included

Studies included in qualitative synthesis
(n =135)

Full-text articles assessed for eligibility
(n =420)

Additional records identified through other sources
(n =19)

Clinical trials registers databases
(n =142)

Records identified through database searching
(n =5286)
