# Supplementary material for: Selective serotonin reuptake inhibitors, and serotonin and norepinephrine reuptake inhibitors for anxiety, obsessive-compulsive, and stress disorders: A 3-level network meta-analysis
Source: PLoS Med. 2021 Jun 10;18(6):e1003664. doi: 10.1371/journal.pmed.1003664 (PMC8224914; doi:10.1371/journal.pmed.1003664)
Supplement: S5 Appendix — (DOCX) [file pmed.1003664.s005.docx]

**S5 Appendix. Studies general information**

| **id** | **PMID /  Other ID** | **Author** | **Title** | **Pub. Status** | **Year of Publication** | **Funding** |
| --- | --- | --- | --- | --- | --- | --- |
| JF10 | 2004379131 | Allgulander | Efficacy of venlafaxine ER in patients with social anxiety disorder: A double-blind, placebo-controlled, parallel-group comparison with paroxetine [1] | published | 2004 | Industry |
| JF11 | 1999304629 | Allgulander C. | Paroxetine in social anxiety disorder: A randomized placebo-controlled study [2] | published | 1999 | Industry |
| JF15 | 2007092774 | Asakura S. | Fluvoxamine treatment of generalized social anxiety disorder in Japan: A randomized double-blind, placebo-controlled study [3] | published | 2006 | Industry |
| JF16 | 11472786 | Asnis | Fluvoxamine in the treatment of panic disorder: a multi-center, double-blind, placebo-controlled study in outpatients [4] | published | 2001 | Industry |
| JF20 | 10665629 | Bakker | Paroxetine, clomipramine, and cognitive therapy in the treatment of panic disorder [5] | published | 1999 | academic |
| JF22 | 2006434703 | Baldwin | Escitalopram and paroxetine in the treatment of generalised anxiety disorder: Randomised, placebo-controlled, double-blind study [6] | published | 2006 | Industry |
| JF25 | 1999268351 | Baldwin D. | Paroxetine in social phobia/social anxiety disorder: Randomised, double- blind, placebo-controlled study [7] | published | 1999 | Industry |
| JF28 | 1998025805 | Ballenger | Double-blind, fixed-dose, placebo-controlled study of paroxetine in the treatment of panic disorder [8] | published | 1998 | Industry |
| JF29 | 2010331033 | Bandelow | Extended-release quetiapine fumarate (quetiapine XR): A once-daily monotherapy effective in generalized anxiety disorder. Data from a randomized, double-blind, placebo-and active-controlled study [9] | published | 2010 | Industry |
| JF3 | 24644106 | Alaka | Efficacy and safety of duloxetine in the treatment of older adult patients with generalized anxiety disorder: a randomized, double-blind, placebo-controlled trial [10] | published | 2014 | Industry |
| JF34 | 2007567996 | Beidel D.C. | SET-C versus fluoxetine in the treatment of childhood social phobia [11] | published | 2007 | Industry |
| JF42 | 12649628 | Birmaher | Fluoxetine for the treatment of childhood anxiety disorders [12] | published | 2003 | governmental or non-profit |
| JF45 | 8422221 | Black | A comparison of fluvoxamine, cognitive therapy, and placebo in the treatment of panic disorder [13] | published | 1993 | academic |
| JF56 | 2005461776 | Bradwejn | Venlafaxine extended-release capsules in panic disorder: Flexible-dose, double-blind, placebo-controlled study [14] | published | 2005 | academic |
| JF59 | 2000137979 | Brady | Efficacy and safety of sertraline treatment of posttraumatic stress disorder: A randomized controlled trial [15] | published | 2000 | Unclear |
| JF61 | 2006335133 | Brawman-Mintzer O. | Sertraline treatment for generalized anxiety disorder: A randomized, double-blind, placebo-controlled study [16] | published | 2006 | Industry |
| JF7 | 2004388355 | Allgulander | Efficacy of sertraline in a 12-week trial for generalized anxiety disorder [17] | published | 2004 | Industry |
| JF72 | 2274626 | Chouinard | Results of a double-blind placebo controlled trial of a new serotonin uptake inhibitor, sertraline, in the treatment of obsessive-compulsive disorder [18] | published | 1990 | Industry |
| JF78 | 1999231913 | Connor K.M. | Fluoxetine in post-traumatic stress disorder. Randomised, double-blind study [19] | published | 1999 | Unclear |
| JF80 | 20455246 | Coric | Multicenter, randomized, double-blind, active comparator and placebo-controlled trial of a corticotropin-releasing factor receptor-1 antagonist in generalized anxiety disorder [20] | published | 2010 | governmental or non-profit |
| JF82 | 2013806668 | Da Costa | Comparison among clomipramine, fluoxetine, and placebo for the treatment of anxiety disorders in children and adolescents [21] | published | 2013 | Industry |
| JF83 | 15877709 | Dahl | Sertraline in generalized anxiety disorder: efficacy in treating the psychic and somatic anxiety factors [22] | published | 2005 | academic |
| JF87 | 1999307984 | Davidson | Efficacy, safety, and tolerability of venlafaxine extended release and buspirone in outpatients with generalized anxiety disorder [23] | published | 1999 | academic |
| JF88 | 2004332730 | Davidson | Escitalopram in the treatment of generalized anxiety disorder: Double-blind, placebo controlled, flexible-dose study [24] | published | 2004 | Industry |
| JF89 | 15206657 | Davidson | Fluvoxamine-controlled release formulation for the treatment of generalized socialanxiety disorder [25] | published | 2004 | Industry |
| JF9 | 17559726 | Koponen | Efficacy of Duloxetine for the Treatment of GeneralizedAnxiety Disorder: Implications for Primary Care Physicians [26] | published | 2007 | Industry |
| JF94 | NA | Asakura S. | A randomized, double-blind, placebo-controlled study of escitalopram in patients with social anxiety disorder in Japan [27] | published | 2016 | Industry |
| LM10 | 1697419 | Den Boer | Serotonin function in panic disorder: a double blind placebo controlled study with fluvoxamine and ritanserin [28] | published | 1990 | Industry |
| LM23 | 2011102179 | Fani N. | Increased neural response to trauma scripts in posttraumatic stress disorder following paroxetine treatment: A pilot study [29] | published | 2011 | Industry |
| LM24 | 2009487779 | Fani N. | Neuropsychological functioning in patients with posttraumatic stress disorder following short-term paroxetine treatment [30] | published | 2009 | Industry |
| LM34 | 2007265412 | Friedman M.J. | Randomized, double-blind comparison of sertraline and placebo for posttraumatic stress disorder in a department of veterans affairs setting [31] | published | 2007 | Industry |
| LM37 | 2000217510 | Gelenberg | Efficacy of venlafaxine extended-release capsules in nondepressed outpatients with generalized anxiety disorder a 6-month randomized controlled trial [32] | published | 2000 | Industry |
| LM39 | 2001231890 | Geller D.A. | Fluoxetine treatment for obsessive-compulsive disorder in children and adolescents: A placebo-controlled clinical trial [33] | published | 2001 | Industry |
| LM4 | 2006486251 | Davidson J. | Treatment of posttraumatic stress disorder with venlafaxine extended release: A 6-month randomized controlled trial [34] | published | 2006 | Industry |
| LM40 | 2004455208 | Geller D.A. | Paroxetine treatment in children and adolescents with obsessive-compulsive disorder: A randomized, multicenter, double-blind, placebo-controlled trial [35] | published | 2004 | Industry |
| LM42 | 2014038390 | Gimenez M. | Functional effects of chronic paroxetine versus placebo on the fear, stress and anxiety brain circuit in Social Anxiety Disorder: Initial validation of an imaging protocol for drug discovery [36] | published | 2013 | Industry |
| LM48 | 1996126650 | Goodman W.K. | Treatment of obsessive-compulsive disorder with fluvoxamine: A multicentre, double-blind, placebo-controlled trial [37] | published | 1996 | Industry |
| LM5 | 2004433622 | Davidson J.R.T. | Fluoxetine, comprehensive cognitive behavioral therapy, and placebo in generalized social phobia [38] | published | 2004 | governmental or non-profit |
| LM50 | 1995114731 | Greist | Double-blind parallel comparison of three dosages of sertraline and placebo in outpatients with obsessive-compulsive disorder [39] | published | 1995 | Industry |
| LM54 | 2007173305 | Hartford | Duloxetine as an SNRI treatment for generalized anxiety disorder: Results from a placebo and active-controlled trial [40] | published | 2007 | Industry |
| LM57 | 10907802 | Hertzberg | Lack of efficacy for fluoxetine in PTSD: a placebo controlled trial in combat veterans [41] | published | 2000 | Industry |
| LM59 | 8227490 | Hoehn-Saric | Effect of fluvoxamine on panic disorder [42] | published | 1993 | academic |
| LM6 | 2001168539 | Davidson J.R.T. | Multicenter, double-blind comparison of sertraline and placebo in the treatment of posttraumatic stress disorder [43] | published | 2001 | Industry |
| LM60 | 2003250843 | Hollander | A double-blind, placebo-controlled study of the efficacy and safety of controlled-release fluvoxamine in patients with obsessive-compulsive disorder [44] | published | 2003 | Industry |
| LM67 | 2143637 | Jenike | A controlled trial of fluvoxamine in obsessive-compulsive disorder: implications for a serotonergic theory [45] | published | 1990 | academic |
| LM69 | 1997265747 | Jenike M.A. | Placebo-controlled trial of fluoxetine and phenelzine for obsessive- compulsive disorder [46] | published | 1997 | governmental or non-profit |
| LM71 | 2004359161 | Kamijima K. | Paroxetine in the treatment of obsessive-compulsive disorder: Randomized, double-blind, placebo-controlled study in Japanese patients [47] | published | 2004 | academic |
| LM72 | 2009168154 | Kasper | Efficacy of pregabalin and venlafaxine-XR in generalized anxiety disorder: Results of a double-blind, placebo-controlled 8-week trial [48] | published | 2009 | Industry |
| LM73 | 2005116331 | Kasper | Escitalopram in the treatment of social anxiety disorder: Randomised, placebo-controlled, flexible-dosage study [49] | published | 2005 | Industry |
| LM74 | 2014307110 | Kasper S. | Lavender oil preparation Silexan is effective in generalized anxiety disorder - A randomized, double-blind comparison to placebo and paroxetine [50] | published | 2014 | academic |
| LM76 | 1995265890 | Katzelnick D.J. | Sertraline for social phobia: A double-blind, placebo-controlled crossover study [51] | published | 1995 | Industry |
| LM86 | 20462466 | Koszycki | A randomized trial of sertraline, self-administered cognitive behavior therapy, and their combination for panic disorder [52] | published | 2011 | Industry |
| LM95 | 28266242 | Li | Effect and safety of sertraline for treat posttraumatic stress disorder: a multicenter randomised controlled study [53] | published | 2017 | academic |
| MC1 | 16175565 | Ledley | Impact of depressive symptoms on the treatment of generalized social anxiety disorder [54] | published | 2005 | governmental or non-profit |
| MC10 | 15003077 | Lepola | Controlled-release paroxetine in the treatment of patients with social anxiety disorder [55] | published | 2004 | Industry |
| MC12 | 2009266799 | Liebowitz | A double-blind, placebo-controlled, parallel-group, flexible-dose study of venlafaxine extended release capsules in adult outpatients with panic disorder [56] | published | 2009 | Industry |
| MC13 | 2002049438 | Liebowitz | A randomized, double-blind, fixed-dose comparison of paroxetine and placebo in the treatment of generalized social anxiety disorder [57] | published | 2002 | Industry |
| MC14 | 2005105216 | Liebowitz | A randomized controlled trial of venlafaxine extended release in generalized social anxiety disorder [58] | published | 2005 | Industry |
| MC15 | 2003299727 | Liebowitz | Efficacy of sertraline in severe generalized social anxiety disorder: Results of a double-blind, placebo-controlled study [59] | published | 2003 | Industry |
| MC16 | 12447029 | Liebowitz | Fluoxetine in children and adolescents with OCD: a placebo-controlled trial [60] | published | 2002 | Industry |
| MC17 | 2005062649 | Liebowitz M.R. | Venlafaxine extended release vs placebo and paroxetine in social anxiety disorder [61] | published | 2005 | Industry |
| MC2 | 2000096828 | Leinonen | Citalopram controls phobic symptoms in patients with panic disorder: Randomized controlled trial [62] | published | 2000 | academic |
| MC20a | 1998228027 | Londborg P.D. | Sertraline in the treatment of panic disorder. A multi-site, double- blind, placebo-controlled, fixed-dose investigation [63] | published | 1998 | Industry |
| MC20b | 1998228027 | Londborg P.D. | Sertraline in the treatment of panic disorder. A multi-site, double- blind, placebo-controlled, fixed-dose investigation [63] | published | 1998 | Industry |
| MC22 | 2013802284 | Mahableshwarkar | A randomised, double-blind, placebo-controlled, duloxetine-referenced study of the efficacy and tolerability of vortioxetine in the acute treatment of adults with generalised anxiety disorder [64] | published | 2013 | Industry |
| MC25 | 2007528912 | March | A Randomized Controlled Trial of Venlafaxine ER Versus Placebo in Pediatric Social Anxiety Disorder [65] | published | 2007 | Industry |
| MC26 | 2004455374 | March | Cognitive-behavior therapy, sertraline, and their combination for children and adolescents with obsessive-compulsive disorder: The pediatric OCD treatment study (POTS) randomized controlled trial [66] | published | 2004 | governmental or non-profit |
| MC28 | 1998400055 | March J.S. | Sertraline in children and adolescents with obsessive-compulsive disorder: A multicenter randomized controlled trial [67] | published | 1998 | Industry |
| MC3 | 2003412218 | Lenox-Smith | A double-blind, randomised, placebo controlled study of venlafaxine XL in patients with generalised anxiety disorder in primary care [68] | published | 2003 | Industry |
| MC31 | 2001420732 | Marshall | Efficacy and safety of paroxetine treatment for chronic PTSD: A fixed-dose, placebo-controlled study [69] | published | 2001 | Industry |
| MC32 | 2007163092 | Marshall | A controlled trial of paroxetine for chronic PTSD, dissociation, and interpersonal problems in mostly minority adults [70] | published | 2007 | Unclear |
| MC33 | 17414240 | Martenyi | Failed efficacy of fluoxetine in the treatment of posttraumatic stress disorder: results of a fixed-dose, placebo-controlled study [71] | published | 2007 | Industry |
| MC34 | 2006287406 | Martenyi | Fluoxetine in the acute treatment and relapse prevention of combat-related post-traumatic stress disorder: Analysis of the veteran group of a placebo-controlled, randomized clinical trial [72] | published | 2006 | Unclear |
| MC38 | 2011677266 | Merideth | Efficacy and tolerability of extended release quetiapine fumarate monotherapy in the acute treatment of generalized anxiety disorder: A randomized, placebo controlled and active-controlled study [73] | published | 2011 | Industry |
| MC39 | 9812120 | Michelson | Outcome assessment and clinical improvement in panic disorder: evidence from a randomized controlled trial of fluoxetine and placebo. The Fluoxetine Panic Disorder Study Group [74] | published | 1998 | Industry |
| MC4 | 2005031032 | Lenze | Efficacy and tolerability of citalopram in the treatment of late-life anxiety disorders: Results from an 8-week randomized, placebo-controlled trial [75] | published | 2005 | academic |
| MC40 | 2002013536 | Michelson D. | Efficacy of usual antidepressant dosing regimens of fluoxetine in panic disorder. Randomised, placebo-controlled trial [76] | published | 2001 | Industry |
| MC42 | 1994091030 | Montgomery | A double-blind, placebo-controlled study of fluoxetine in patients with DSM-III-R obsessive-compulsive disorder [77] | published | 1993 | Unclear |
| MC44 | 2006302530 | Montgomery | Efficacy and safety of pregabalin in the treatment of generalized anxiety disorder: A 6-week, multicenter, randomized, double-blind, placebo-controlled comparison of pregabalin and venlafaxine [78] | published | 2006 | Industry |
| MC45 | 2001077435 | Montgomery | Citalopram 20 mg, 40 mg and 60 mg are all effective and well tolerated compared with placebo in obsessive-compulsive disorder [79] | published | 2001 | Industry |
| MC51 | 9160622 | Nair | Comparison of fluvoxamine, imipramine, and placebo in the treatment of outpatients with panic disorder [80] | published | 1996 | Industry |
| MC55 | 18485261 | Nicolini | Improvement of psychic and somatic symptoms in adult patients with generalized anxiety disorder: examination from a duloxetine, venlafaxine extended-release and placebo-controlled trial [81] | published | 2008 | Unclear |
| MC56 | 2004493169 | Nimatoudis I. | Remission rates with venlafaxine extended release in Greek outpatients with generalized anxiety disorder. A double-blind, randomized, placebo [82] | published | 2004 | Unclear |
| MC6 | 2009047569 | Lenze | Escitalopram for older adults with generalized anxiety disorder: A randomized controlled trial [83] | published | 2009 | governmental or non-profit |
| MC62 | 21349225 | Panahi | A randomized, double-blind, placebo-controlled trial on the efficacy and tolerability of sertraline in Iranian veterans with post-traumatic stress disorder [84] | published | 2011 | academic |
| MC73 | 2007466592 | Pollack | A randomized controlled trial of venlafaxine ER and paroxetine in the treatment of outpatients with panic disorder [85] | published | 2007 | Industry |
| MC77 | 11411817 | Pollack | Paroxetine in the treatment of generalized anxiety disorder: results of a placebo-controlled, flexible-dosage trial [86] | published | 2001 | Industry |
| MC79 | 2007096532 | Pollack | A double-blind study of the efficacy of venlafaxine extended-release, paroxetine, and placebo in the treatment of panic disorder [87] | published | 2006 | Industry |
| MC81 | 1997005505 | Pollack | Venlafaxine for panic disorder: Results from a double-blind, placebo- controlled study [88] | published | 1996 | Unclear |
| MC82 | 1998374883 | Pollack M.H. | Sertraline in the treatment of panic disorder: A flexible-dose multicenter trial [89] | published | 1998 | Industry |
| MJ1 | 2004408566 | Rickels | A double-blind, placebo-controlled study of a flexible dose of venlafaxine ER in adult outpatients with generalized social anxiety disorder [90] | published | 2004 | Unclear |
| MJ14 | 2008171568 | Rynn | Efficacy and safety of duloxetine in the treatment of generalized anxiety disorder: A flexible-dose, progressive-titration, placebo-controlled trial [91] | published | 2007 | Industry |
| MJ16 | 2001420736 | Rynn M.A. | Placebo-controlled trial of sertraline in the treatment of children with generalized anxiety disorder [92] | published | 2001 | governmental or non-profit |
| MJ17 | 1998292538 | Sandmann J. | Fluvoxamine or placebo in the treatment of panic disorder and relationship to blood concentrations of fluvoxamine [93] | published | 1998 | academic |
| MJ2 | 2000222191 | Rickels | Efficacy of extended-release Venlafaxine in nondepressed outpatients with generalized anxiety disorder [94] | published | 2000 | Industry |
| MJ22 | 1996223201 | Sharp D.M. | Global measures of outcome in a controlled comparison of pharmacological and psychological treatment of panic disorder and agoraphobia in primary care [95] | published | 1997 | Industry |
| MJ25 | 15669886 | Sheehan | Efficacy and tolerability of controlled-release paroxetine in the treatment of panic disorder [96] | published | 2005 | Industry |
| MJ3 | 2005347556 | Rickels K. | Paroxetine treatment of generalized anxiety disorder: A double-blind, placebo-controlled study [97] | published | 2003 | Industry |
| MJ36 | 2003496184 | Stahl | Escitalopram in the Treatment of Panic Disorder: A Randomized, Double-Blind, Placebo-Controlled Trial [98] | published | 2003 | Industry |
| MJ4 | 1429406 | Riddle | Double-blind, crossover trial of fluoxetine and placebo in children and adolescents with obsessive-compulsive disorder [99] | published | 1992 | governmental or non-profit |
| MJ42 | 2005044275 | Stein | Efficacy of low and higher dose extended-release venlafaxine in generalized social anxiety disorder: A 6-month randomized controlled trial [100] | published | 2004 | Industry |
| MJ44 | 2007198511 | Stein | Escitalopram in obsessive-compulsive disorder: A randomized, placebo-controlled, paroxetine-referenced, fixed-dose, 24-week study [101] | published | 2007 | Industry |
| MJ5 | 2001046511 | Riddle M.A. | Fluvoxamine for children and adolescents with obsessive-compulsive disorder: A randomized, controlled, multicenter trial [102] | published | 2001 | Industry |
| MJ53 | 1999166603 | Stein M.B. | Fluvoxamine treatment of social phobia (social anxiety disorder): A double-blind, placebo-controlled study [103] | published | 1999 | Industry |
| MJ54 | 1998297625 | Stein M.B. | Paroxetine treatment of generalized social phobia (social anxiety disorder): A randomized controlled trial [104] | published | 1998 | Industry |
| MJ56 | 2015802599 | Strawn | A randomized, placebo-controlled study of duloxetine for the treatment of children and adolescents with generalized anxiety disorder [105] | published | 2015 | Industry |
| MJ6 | 2011001857 | Robb A.S. | Sertraline treatment of children and adolescents with posttraumatic stress disorder: A double-blind, placebo-controlled trial [106] | published | 2010 | Industry |
| MJ64 | 14608246 | Tucker | Can physiologic assessment and side effects tease out differences in PTSD trials? A double-blind comparison of citalopram, sertraline, and placebo [107] | published | 2003 | Industry |
| MJ66 | 2001431494 | Tucker P. | Paroxetine in the treatment of chronic posttraumatic stress disorder: Results of a placebo-controlled, flexible-dosage trial [108] | published | 2001 | Unclear |
| MJ7 | 2000093467 | Rolland P.D. | Treatment of generalised anxiety disorder with venlafaxine XR. A randomised, double-blind trial in comparison with buspirone and placebo [109] | published | 2000 | Industry |
| MJ70 | 2001050465 | Van Ameringen M.A. | Sertraline treatment of generalized social phobia: A 20-week, double-blind, placebo-controlled study [110] | published | 2001 | Industry |
| MJ71 | 2007077920 | Van Der Kolk | A Randomized clinical trial of eye movement desensitization and reprocessing (EMDR), fluoxetine, and pill placebo in the treatment of posttraumatic stress disorder: treatment effects and long-term maintenance [111] | published | 2007 | Industry |
| MJ73 | 1994203727 | Van Vliet I.M. | Psychopharmacological treatment of social phobia; a double blind placebo controlled study with fluvoxamine [112] | published | 1993 | governmental or non-profit |
| MJ77 | 9330022 | Wade | The effect of citalopram in panic disorder [113] | published | 1997 | Unclear |
| MJ78 | 2004471921 | Wagner | A multicenter, randomized, double-blind, placebo-controlled trial of paroxetine in children and adolescents with social anxiety disorder [114] | published | 2004 | Unclear |
| MJ79 | 18974308 | Walkup | Cognitive behavioral therapy, sertraline, or a combination in childhood anxiety [115] | published | 2008 | Industry |
| MJ80 | 11323729 | Walkup | Fluvoxamine for the treatment of anxiety disorders in children and adolescents. The Research Unit on Pediatric Psychopharmacology Anxiety Study Group [116] | published | 2001 | governmental or non-profit |
| MJ84 | 2004049533 | Westenberg | A Double-Blind Placebo-Controlled Study of Controlled Release Fluvoxamine for the Treatment of Generalized Social Anxiety Disorder [117] | published | 2004 | Industry |
| MJ85 | 2528158 | Westenberg | Selective monoamine uptake inhibitors and a serotonin antagonist in the treatment of panic disorder [118] | published | 1989 | Industry |
| MJ89 | 2011586428 | Wu | Duloxetine versus placebo in the treatment of patients with generalized anxiety disorder in China [119] | published | 2011 | academic |
| MJ93 | 2002132412 | Zohar | Double-blind placebo-controlled pilot study of sertraline in military veterans with posttraumatic stress disorder [120] | published | 2002 | Industry |
| MJ94 | 1996302206 | Zohar | Paroxetine versus clomipramine in the treatment of obsessive-compulsive disorder [121] | published | 1996 | Industry |
| MJ96a | 2192564 | Jenike | Sertraline in Obsessive-Compulsive Disorder: A double-Blind Comparison With Placebo [122] | published | 1990 | Industry |
| MJ96b | 2192564 | Jenike | Sertraline in Obsessive-Compulsive Disorder: A double-Blind Comparison With Placebo [122] | published | 1990 | Industry |
| UNG9 | NKF100110 | Unknown | A Randomized, Double-Blind, Parallel-Group, Placebo-Controlled, Forced-Dose Titration Study Evaluating the Efficacy and Safety of a New Chemical Entity (NCE) and Paroxetine in Subjects with Social Anxiety Disorder. | unpublished | unpublished | Industry |
| UNG1 | SCT-MD-05 | Unknown | Escitalopram in the treatment of generalized anxiety disorder: Double-blind, placebo controlled, flexible-dose study | unpublished | unpublished | Industry |
| UNG10 | NKP102280 | Unknown | A double-blind, double dummy, placebo-controlled, randomised, parallel group positron emission tomography (PET) study to investigate the effects of a 8 week administration of a new compound and Paroxetine in combination or Paroxetine alone (7.5 mg) on regional cerebral blood flow (rCBF) during a Public Speaking test in subjects affected by social anxiety disorder (SAD). | unpublished | unpublished | Industry |
| UNG11 | BRL-029060/CPMS- 116 | Unknown | Paroxetine versus Placebo in the Treatment of Obsessive-Compulsive Disorder | unpublished | unpublished | Industry |
| UNG12 | MY- 1028/BRL-029060/1/CPMS-118 | Unknown | Paroxetine versus Clomipramine and Placebo in the Treatment of Obsessive-Compulsive Disorder | unpublished | unpublished | Industry |
| UNG17 | NKP103401 | Unknown | A randomized, double-blind, parallel group, placebo-controlled fixed dose study comparing the efficacy and safety of New Chemical Entity (NCE))/Paroxetine combination of Paroxetine monotherapy to placebo in subjects with Social Anxiety Disorder (SAD) | unpublished | unpublished | Industry |
| UNG2 | SCT-MD-06 | Unknown | Flexible-dose comparison of the safety and efficacy of Escitalopram and placebo in the treatment of generalized anxiety disorder | unpublished | unpublished | Industry |
| UNG3 | NCT01933919 | Unknown | A phase 3 study of fluvoxamine (SME3110) in pediatric/adolescent patients with obsessive compulsive disorder | unpublished | unpublished | Industry |
| UNG6 | GSK 637 | Hewett | A double-blind, placebo controlled study to evaluate the efficacy and tolerability of paroxetine in patients with generalized anxiety disorder (GAD) | unpublished | unpublished | Industry |
| UNG7 | GSK 791 | Unknown | A randomized, double-blind, placebo-controlled, flexible dosage trial to evaluate the efficacy and tolerability of Paroxetine CR in patients with generalized anxiety disorder (GAD) | unpublished | unpublished | Industry |
| UNG8 | Sonne draft | Sonne | The effect of Paroxetine in the treatment of comorbid PTSD and substance dependence | unpublished | unpublished | Industry |
| UPD3 | 10.4172/2167-1044.S1-014 | Liebowitz MR | A 12-Week Double-Blind, Placebo-Controlled, Flexible-Dose Trial of Desvenlafaxine Extended-Release Tablets in Generalized Social Anxiety Disorder | published | 2015 | Industry |
| UPD8 | 32857933 | Strawn JR | Escitalopram in Adolescents With Generalized Anxiety Disorder: A Double-Blind, Randomized, Placebo-Controlled Study | published | 2015 | governmental or non-profit |

**References**

1. Allgulander C, Mangano R, Zhang J, Dahl AA, Lepola U, Sjödin I, et al. Efficacy of Venlafaxine ER in patients with social anxiety disorder: a double-blind, placebo-controlled, parallel-group comparison with paroxetine. Hum Psychopharmacol Clin Exp. 2004;19: 387–396. doi:10.1002/hup.602

2. Allgulander C. Paroxetine in social anxiety disorder: a randomized placebo-controlled study. Acta Psychiatr Scand. 1999;100: 193–198. doi:10.1111/j.1600-0447.1999.tb10845.x

3. Asakura S, Tajima O, Koyama T. Fluvoxamine treatment of generalized social anxiety disorder in Japan: a randomized double-blind, placebo-controlled study. Int J Neuropsychopharm. 2007;10: 263–274. doi:10.1017/S1461145706006602

4. Asnis GM, Hameedi FA, Goddard AW, Potkin SG, Black D, Jameel M, et al. Fluvoxamine in the treatment of panic disorder: a multi-center, double-blind, placebo-controlled study in outpatients. Psychiatry Res. 2001;103: 1–14. doi:10.1016/S0165-1781(01)00265-7

5. Bakker A, van Dyck R, Spinhoven P, van Balkom AJ. Paroxetine, clomipramine, and cognitive therapy in the treatment of panic disorder. J Clin Psychiatry. 1999;60: 831–838. doi:10.4088/jcp.v60n1205

6. Baldwin DS, Huusom AKT, Maehlum E. Escitalopram and paroxetine in the treatment of generalised anxiety disorder: randomised, placebo-controlled, double-blind study. Br J Psychiatry. 2006;189: 264–272. doi:10.1192/bjp.bp.105.012799

7. Baldwin D, Bobes J, Stein DJ, Scharwächter I, Faure M. Paroxetine in social phobia/social anxiety disorder. Randomised, double-blind, placebo-controlled study. Paroxetine Study Group. Br J Psychiatry. 1999;175: 120–126. doi:10.1192/bjp.175.2.120

8. Ballenger JC, Wheadon DE, Steiner M, Bushnell W, Gergel IP. Double-blind, fixed-dose, placebo-controlled study of paroxetine in the treatment of panic disorder. Am J Psychiatry. 1998;155: 36–42. doi:10.1176/ajp.155.1.36

9. Bandelow B, Chouinard G, Bobes J, Ahokas A, Eggens I, Liu S, et al. Extended-release quetiapine fumarate (quetiapine XR): a once-daily monotherapy effective in generalized anxiety disorder. Data from a randomized, double-blind, placebo- and active-controlled study. Int J Neuropsychopharm. 2010;13: 305–320. doi:10.1017/S1461145709990423

10. Alaka KJ, Noble W, Montejo A, Dueñas H, Munshi A, Strawn JR, et al. Efficacy and safety of duloxetine in the treatment of older adult patients with generalized anxiety disorder: a randomized, double-blind, placebo-controlled trial. Int J Geriatr Psychiatry. 2014;29: 978–986. doi:10.1002/gps.4088

11. Beidel DC, Turner SM, Sallee FR, Ammerman RT, Crosby LA, Pathak S. SET-C Versus Fluoxetine in the Treatment of Childhood Social Phobia. J Am Acad Child Adolesc Psychiatry. 2007;46: 1622–1632. doi:10.1097/chi.0b013e318154bb57

12. Birmaher B, Axelson DA, Monk K, Kalas C, Clark DB, Ehmann M, et al. Fluoxetine for the treatment of childhood anxiety disorders. J Am Acad Child Adolesc Psychiatry. 2003;42: 415–423. doi:10.1097/01.CHI.0000037049.04952.9F

13. Black DW, Wesner R, Bowers W, Gabel J. A comparison of fluvoxamine, cognitive therapy, and placebo in the treatment of panic disorder. Arch Gen Psychiatry. 1993;50: 44–50. doi:10.1001/archpsyc.1993.01820130046008

14. Bradwejn J, Ahokas A, Stein DJ, Salinas E, Emilien G, Whitaker T. Venlafaxine extended-release capsules in panic disorder: Flexible-dose, double-blind, placebo-controlled study. Br J Psychiatry. 2005;187: 352–359. doi:10.1192/bjp.187.4.352

15. Brady K, Pearlstein T, Asnis GM, Baker D, Rothbaum B, Sikes CR, et al. Efficacy and safety of sertraline treatment of posttraumatic stress disorder: a randomized controlled trial. JAMA. 2000;283: 1837–1844. doi:10.1001/jama.283.14.1837

16. Brawman-Mintzer O, Knapp RG, Rynn M, Carter RE, Rickels K. Sertraline treatment for generalized anxiety disorder: a randomized, double-blind, placebo-controlled study. J Clin Psychiatry. 2006;67: 874–881. doi:10.4088/jcp.v67n0603

17. Allgulander C. Efficacy of Sertraline in a 12-Week Trial for Generalized Anxiety Disorder. Am J Psychiatry. 2004;161: 1642–1649. doi:10.1176/appi.ajp.161.9.1642

18. Chouinard G, Goodman W, Greist J, Jenike M, Rasmussen S, White K, et al. Results of a double-blind placebo controlled trial of a new serotonin uptake inhibitor, sertraline, in the treatment of obsessive-compulsive disorder. Psychopharmacol Bull. 1990;26: 279–284.

19. Connor KM, Sutherland SM, Tupler LA, Malik ML, Davidson JR. Fluoxetine in post-traumatic stress disorder. Randomised, double-blind study. Br J Psychiatry. 1999;175: 17–22. doi:10.1192/bjp.175.1.17

20. Coric V, Feldman HH, Oren DA, Shekhar A, Pultz J, Dockens RC, et al. Multicenter, randomized, double-blind, active comparator and placebo-controlled trial of a corticotropin-releasing factor receptor-1 antagonist in generalized anxiety disorder. Depress Anxiety. 2010;27: 417–425. doi:10.1002/da.20695

21. da Costa CZG, de Morais RMCB, Zanetta DMT, Turkiewicz G, Lotufo Neto F, Morikawa M, et al. Comparison among clomipramine, fluoxetine, and placebo for the treatment of anxiety disorders in children and adolescents. J Child Adolesc Psychopharmacol. 2013;23: 687–692. doi:10.1089/cap.2012.0110

22. Dahl AA, Ravindran A, Allgulander C, Kutcher SP, Austin C, Burt T. Sertraline in generalized anxiety disorder: efficacy in treating the psychic and somatic anxiety factors. Acta Psychiatr Scand. 2005;111: 429–435. doi:10.1111/j.1600-0447.2005.00529.x

23. Davidson JRT, DuPont RL, Hedges D, Haskins JT. Efficacy, Safety, and Tolerability of Venlafaxine Extended Release and Buspirone in Outpatients With Generalized Anxiety Disorder. J Clin Psychiatry. 1999;60: 528–535. doi:10.4088/JCP.v60n0805

24. Davidson JRT, Bose A, Korotzer A, Zheng H. Escitalopram in the treatment of generalized anxiety disorder: double-blind, placebo controlled, flexible-dose study. Depress Anxiety. 2004;19: 234–240. doi:10.1002/da.10146

25. Davidson J, Yaryura-Tobias J, DuPont R, Stallings L, Barbato LM, van der Hoop RG, et al. Fluvoxamine-controlled release formulation for the treatment of generalized social anxiety disorder. J Clin Psychopharmacol. 2004;24: 118–125. doi:10.1097/01.jcp.0000106222.36344.96

26. Koponen H, Allgulander C, Erikson J, Dunayevich E, Pritchett Y, Detke MJ, et al. Efficacy of Duloxetine for the Treatment of Generalized Anxiety Disorder: Implications for Primary Care Physicians. Prim Care Companion J Clin Psychiatry. 2007;09: 100–107. doi:10.4088/PCC.v09n0203

27. Asakura S, Hayano T, Hagino A, Koyama T. A randomized, double-blind, placebo-controlled study of escitalopram in patients with social anxiety disorder in Japan. Curr Med Res Opin. 2016;32: 749–757. doi:10.1185/03007995.2016.1146663

28. Den Boer JA, Westenberg HGM. Serotonin function in panic disorder: a double blind placebo controlled study with fluvoxamine and ritanserin. Psychopharmacology. 1990;102: 85–94. doi:10.1007/BF02245749

29. Fani N, Ashraf A, Afzal N, Jawed F, Kitayama N, Reed L, et al. Increased neural response to trauma scripts in posttraumatic stress disorder following paroxetine treatment: A pilot study. Neurosci Lett. 2011;491: 196–201. doi:10.1016/j.neulet.2011.01.037

30. Fani N, Kitayama N, Ashraf A, Reed L, Afzal N, Jawed F, et al. Neuropsychological functioning in patients with posttraumatic stress disorder following short-term paroxetine treatment. Psychopharmacol Bull. 2009;42: 53–68.

31. Friedman MJ, Marmar CR, Baker DG, Sikes CR, Farfel GM. Randomized, Double-Blind Comparison of Sertraline and Placebo for Posttraumatic Stress Disorder in a Department of Veterans Affairs Setting. J Clin Psychiatry. 2007;68: 711–720. doi:10.4088/JCP.v68n0508

32. Gelenberg AJ, Lydiard RB, Rudolph RL, Aguiar L, Haskins JT, Salinas E. Efficacy of Venlafaxine Extended-Release Capsules in Nondepressed Outpatients With Generalized Anxiety Disorder: A 6-Month Randomized Controlled Trial. JAMA. 2000;283: 3082–3088. doi:10.1001/jama.283.23.3082

33. Geller DA, Hoog SL, Heiligenstein JH, Ricardi RK, Tamura R, Kluszynski S, et al. Fluoxetine treatment for obsessive-compulsive disorder in children and adolescents: a placebo-controlled clinical trial. J Am Acad Child Adolesc Psychiatry. 2001;40: 773–779. doi:10.1097/00004583-200107000-00011

34. Davidson J, Baldwin D, Stein DJ, Kuper E, Benattia I, Ahmed S, et al. Treatment of Posttraumatic Stress Disorder With Venlafaxine Extended Release: A 6-Month Randomized Controlled Trial. Arch Gen Psychiatry. 2006;63: 1158–1165. doi:10.1001/archpsyc.63.10.1158

35. Geller DA, Wagner KD, Emslie G, Murphy T, Carpenter DJ, Wetherhold E, et al. Paroxetine treatment in children and adolescents with obsessive-compulsive disorder: a randomized, multicenter, double-blind, placebo-controlled trial. J Am Acad Child Adolesc Psychiatry. 2004;43: 1387–1396. doi:10.1097/01.chi.0000138356.29099.f1

36. Giménez M, Ortiz H, Soriano-Mas C, López-Solà M, Farré M, Deus J, et al. Functional effects of chronic paroxetine versus placebo on the fear, stress and anxiety brain circuit in Social Anxiety Disorder: Initial validation of an imaging protocol for drug discovery. Eur Neuropsychopharmacol. 2014;24: 105–116. doi:10.1016/j.euroneuro.2013.09.004

37. Goodman WK, Kozak MJ, Liebowitz M, White KL. Treatment of obsessive-compulsive disorder with fluvoxamine: a multicentre, double-blind, placebo-controlled trial. Int Clin Psychopharmacol. 1996;11: 21–29.

38. Davidson JRT, Foa EB, Huppert JD, Keefe FJ, Franklin ME, Compton JS, et al. Fluoxetine, Comprehensive Cognitive Behavioral Therapy, and Placeboin Generalized Social Phobia. Arch Gen Psychiatry. 2004;61: 1005–1013. doi:10.1001/archpsyc.61.10.1005

39. Greist J, Chouinard G, DuBoff E, Halaris A, Kim SW, Koran L, et al. Double-blind parallel comparison of three dosages of sertraline and placebo in outpatients with obsessive-compulsive disorder. Arch Gen Psychiatry. 1995;52: 289–295.

40. Hartford J, Kornstein S, Liebowitz M, Pigott T, Russell J, Detke M, et al. Duloxetine as an SNRI treatment for generalized anxiety disorder: results from a placebo and active-controlled trial: Int Clin Psychopharmacol. 2007;22: 167–174. doi:10.1097/YIC.0b013e32807fb1b2

41. Hertzberg M, Feldman M, Beckham J, Kudler H, Davidson J. Lack of Efficacy for Fluoxetine in PTSD: A Placebo Controlled Trial in Combat Veterans. Ann of Clinical Psychiatry. 2000;12: 101–105. doi:10.3109/10401230009147096

42. Hoehn-Saric R, McLeod DR, Hipsley PA. Effect of fluvoxamine on panic disorder. J Clin Psychopharmacol. 1993;13: 321–326.

43. Davidson JR, Rothbaum BO, van der Kolk BA, Sikes CR, Farfel GM. Multicenter, double-blind comparison of sertraline and placebo in the treatment of posttraumatic stress disorder. Arch Gen Psychiatry. 2001;58: 485–492. doi:10.1001/archpsyc.58.5.485

44. Hollander E, Koran LM, Goodman WK, Greist JH, Ninan PT, Yang H, et al. A Double-Blind, Placebo-Controlled Study of the Efficacy and Safety of Controlled-Release Fluvoxamine in Patients With Obsessive-Compulsive Disorder. J Clin Psychiatry. 2003;64: 640–647. doi:10.4088/JCP.v64n0604

45. Jenike MA, Hyman S, Baer L, Holland A, Minichiello WE, Buttolph L, et al. A controlled trial of fluvoxamine in obsessive-compulsive disorder: implications for a serotonergic theory. Am J Psychiatry. 1990;147: 1209–1215. doi:10.1176/ajp.147.9.1209

46. Jenike MA, Baer L, Minichiello WE, Rauch SL, Buttolph ML. Placebo-controlled trial of fluoxetine and phenelzine for obsessive-compulsive disorder. Am J Psychiatry. 1997;154: 1261–1264. doi:10.1176/ajp.154.9.1261

47. Kamijima K, Murasaki M, Asai M, Higuchi T, Nakajima T, Taga C, et al. Paroxetine in the treatment of obsessive-compulsive disorder: randomized, double-blind, placebo-controlled study in Japanese patients. Psychiatry Clin Neurosci. 2004;58: 427–433. doi:10.1111/j.1440-1819.2004.01278.x

48. Kasper S, Herman B, Nivoli G, Ameringen MV, Petralia A, Mandel FS, et al. Efficacy of pregabalin and venlafaxine-XR in generalized anxiety disorder: results of a double-blind, placebo-controlled 8-week trial: Int Clin Psychopharmacol. 2009;24: 87–96. doi:10.1097/YIC.0b013e32831d7980

49. Kasper S, Stein DJ, Loft H, Nil R. Escitalopram in the treatment of social anxiety disorder: Randomised, placebo-controlled, flexible-dosage study. Br J Psychiatry. 2005;186: 222–226. doi:10.1192/bjp.186.3.222

50. Kasper S, Gastpar M, Müller WE, Volz H-P, Möller H-J, Schläfke S, et al. Lavender oil preparation Silexan is effective in generalized anxiety disorder – a randomized, double-blind comparison to placebo and paroxetine. Int J Neuropsychopharm. 2014;17: 859–869. doi:10.1017/S1461145714000017

51. Katzelnick DJ, Kobak KA, Greist JH, Jefferson JW, Mantle JM, Serlin RC. Sertraline for social phobia: a double-blind, placebo-controlled crossover study. Am J Psychiatry. 1995;152: 1368–1371. doi:10.1176/ajp.152.9.1368

52. Koszycki D, Taljaard M, Segal Z, Bradwejn J. A randomized trial of sertraline, self-administered cognitive behavior therapy, and their combination for panic disorder. Psychol Med. 2011;41: 373–383. doi:10.1017/S0033291710000930

53. Li W, Ma Y-B, Yang Q, Li B-L, Meng Q-G, Zhang Y. Effect and safety of sertraline for treat posttraumatic stress disorder: a multicenter randomised controlled study. Int J Psychiatry Clin Pract. 2017;21: 151–155. doi:10.1080/13651501.2017.1291838

54. Ledley DR, Huppert JD, Foa EB, Davidson JRT, Keefe FJ, Potts NLS. Impact of depressive symptoms on the treatment of generalized social anxiety disorder. Depress Anxiety. 2005;22: 161–167. doi:10.1002/da.20121

55. Lepola U, Bergtholdt B, St Lambert J, Davy KL, Ruggiero L. Controlled-release paroxetine in the treatment of patients with social anxiety disorder. J Clin Psychiatry. 2004;65: 222–229. doi:10.4088/jcp.v65n0213

56. Liebowitz MR, Asnis G, Mangano R, Tzanis E. A Double-Blind, Placebo-Controlled, Parallel-Group, Flexible-Dose Study of Venlafaxine Extended Release Capsules in Adult Outpatients With Panic Disorder. J Clin Psychiatry. 2009;70: 550–561. doi:10.4088/JCP.08m04238

57. Liebowitz MR, Stein MB, Tancer M, Carpenter D, Oakes R, Pitts CD. A Randomized, Double-Blind, Fixed-Dose Comparison of Paroxetine and Placebo in the Treatment of Generalized Social Anxiety Disorder. J Clin Psychiatry. 2002;63: 66–74. doi:10.4088/JCP.v63n0113

58. Liebowitz MR, Mangano RM, Bradwejn J, Asnis G, SAD Study Group. A randomized controlled trial of venlafaxine extended release in generalized social anxiety disorder. J Clin Psychiatry. 2005;66: 238–247. doi:10.4088/jcp.v66n0213

59. Liebowitz MR, DeMartinis NA, Weihs K, Londborg PD, Smith WT, Chung H, et al. Efficacy of sertraline in severe generalized social anxiety disorder: results of a double-blind, placebo-controlled study. J Clin Psychiatry. 2003;64: 785–792. doi:10.4088/jcp.v64n0708

60. Liebowitz MR, Turner SM, Piacentini J, Beidel DC, Clarvit SR, Davies SO, et al. Fluoxetine in children and adolescents with OCD: a placebo-controlled trial. J Am Acad Child Adolesc Psychiatry. 2002;41: 1431–1438. doi:10.1097/00004583-200212000-00014

61. Liebowitz MR, Gelenberg AJ, Munjack D. Venlafaxine Extended Release vs Placebo and Paroxetine in Social Anxiety Disorder. Arch Gen Psychiatry. 2005;62: 190–198. doi:10.1001/archpsyc.62.2.190

62. Leinonen E, Lepola U, Koponen H, Turtonen J, Wade A, Lehto H. Citalopram controls phobic symptoms in patients with panic disorder: randomized controlled trial. J Psychiatry Neurosci. 2000;25: 24–32.

63. Londborg PD, Wolkow R, Smith WT, Duboff E, England D, Ferguson J, et al. Sertraline in the treatment of panic disorder: A multi-site, double-blind, placebo-controlled, fixed-dose investigation. Br J Psychiatry. 1998;173: 54–60. doi:10.1192/bjp.173.1.54

64. Mahableshwarkar AR, Jacobsen PL, Chen Y, Simon JS. A randomised, double-blind, placebo-controlled, duloxetine-referenced study of the efficacy and tolerability of vortioxetine in the acute treatment of adults with generalised anxiety disorder. Int J Clin Pract. 2014;68: 49–59. doi:10.1111/ijcp.12328

65. March JS, Entusah AR, Rynn M, Albano AM, Tourian KA. A Randomized Controlled Trial of Venlafaxine ER Versus Placebo in Pediatric Social Anxiety Disorder. Biol Psychiatry. 2007;62: 1149–1154. doi:10.1016/j.biopsych.2007.02.025

66. Walkup JT, Albano AM, Piacentini J, Birmaher B, Compton SN, Sherrill JT, et al. Cognitive behavioral therapy, sertraline, or a combination in childhood anxiety. N Engl J Med. 2008;359: 2753–2766. doi:10.1056/NEJMoa0804633

67. March JS, Biederman J, Wolkow R, Safferman A, Mardekian J, Cook EH, et al. Sertraline in children and adolescents with obsessive-compulsive disorder: a multicenter randomized controlled trial. JAMA. 1998;280: 1752–1756. doi:10.1001/jama.280.20.1752

68. Lenox-Smith AJ, Reynolds A. A double-blind, randomised, placebo controlled study of venlafaxine XL in patients with generalised anxiety disorder in primary care. Br J Gen Pract. 2003;53: 772–777.

69. Marshall RD, Beebe KL, Oldham M, Zaninelli R. Efficacy and Safety of Paroxetine Treatment for Chronic PTSD: A Fixed-Dose, Placebo-Controlled Study. Am J Psychiatry. 2001;158: 1982–1988. doi:10.1176/appi.ajp.158.12.1982

70. Marshall RD, Lewis-Fernandez R, Blanco C, Simpson HB, Lin S-H, Vermes D, et al. A controlled trial of paroxetine for chronic PTSD, dissociation, and interpersonal problems in mostly minority adults. Depress Anxiety. 2007;24: 77–84. doi:10.1002/da.20176

71. Martenyi F, Brown EB, Caldwell CD. Failed Efficacy of Fluoxetine in the Treatment of Posttraumatic Stress Disorder: Results of a Fixed-Dose, Placebo-Controlled Study. J Clin Psychopharmacol. 2007;27: 166–170. doi:10.1097/JCP.0b013e31803308ce

72. Martenyi F, Soldatenkova V. Fluoxetine in the acute treatment and relapse prevention of combat-related post-traumatic stress disorder: Analysis of the veteran group of a placebo-controlled, randomized clinical trial. Eur Neuropsychopharmacol. 2006;16: 340–349. doi:10.1016/j.euroneuro.2005.10.007

73. Merideth C, Cutler AJ, She F, Eriksson H. Efficacy and tolerability of extended release quetiapine fumarate monotherapy in the acute treatment of generalized anxiety disorder: a randomized, placebo controlled and active-controlled study. Int Clin Psychopharmacol. 2012;27: 40–54. doi:10.1097/YIC.0b013e32834d9f49

74. Michelson D, Lydiard RB, Pollack MH, Tamura RN, Hoog SL, Tepner R, et al. Outcome Assessment and Clinical Improvement in Panic Disorder: Evidence From a Randomized Controlled Trial of Fluoxetine and Placebo. AJP. 1998;155: 1570–1577. doi:10.1176/ajp.155.11.1570

75. Lenze EJ, Mulsant BH, Shear MK, Dew MA, Miller MD, Pollock BG, et al. Efficacy and Tolerability of Citalopram in the Treatment of Late-Life Anxiety Disorders: Results From an 8-Week Randomized, Placebo-Controlled Trial. Am J Psychiatry. 2005;162: 146–150. doi:10.1176/appi.ajp.162.1.146

76. Michelson D, Allgulander C, Dantendorfer K, Knezevic A, Maierhofer D, Micev V, et al. Efficacy of usual antidepressant dosing regimens of fluoxetine in panic disorder: Randomised, placebo-controlled trial. Br J Psychiatry. 2001;179: 514–518. doi:10.1192/bjp.179.6.514

77. Montgomery SA, McIntyre A, Osterheider M, Sarteschi P, Zitterl W, Zohar J, et al. A double-blind, placebo-controlled study of fluoxetine in patients with DSM-III-R obsessive-compulsive disorder. The Lilly European OCD Study Group. Eur Neuropsychopharmacol. 1993;3: 143–152. doi:10.1016/0924-977x(93)90266-o

78. Montgomery SA, Tobias K, Zornberg GL, Kasper S, Pande AC. Efficacy and Safety of Pregabalin in the Treatment of Generalized Anxiety Disorder: A 6-Week, Multicenter, Randomized, Double-Blind, Placebo-Controlled Comparison of Pregabalin and Venlafaxine. J Clin Psychiatry. 2006;67: 771–782. doi:10.4088/JCP.v67n0511

79. Montgomery SA, Kasper S, Stein DJ, Hedegaard KB, Lemming OM. Citalopram 20 mg, 40 mg and 60 mg are all effective and well tolerated compared with placebo in obsessive-compulsive disorder: Int Clin Psychopharmacol. 2001;16: 75–86. doi:10.1097/00004850-200103000-00002

80. Nair NP, Bakish D, Saxena B, Amin M, Schwartz G, West TE. Comparison of fluvoxamine, imipramine, and placebo in the treatment of outpatients with panic disorder. Anxiety. 1996;2: 192–198. doi:10.1002/(SICI)1522-7154(1996)2:4<192::AID-ANXI6>3.0.CO;2-Q

81. Nicolini H, Bakish D, Duenas H, Spann M, Erickson J, Hallberg C, et al. Improvement of psychic and somatic symptoms in adult patients with generalized anxiety disorder: examination from a duloxetine, venlafaxine extended-release and placebo-controlled trial. Psychol Med. 2009;39: 267–276. doi:10.1017/S0033291708003401

82. Nimatoudis I, Zissis NP, Kogeorgos J, Theodoropoulou S, Vidalis A, Kaprinis G. Remission rates with venlafaxine extended release in Greek outpatients with generalized anxiety disorder. A double-blind, randomized, placebo controlled study: Int Clin Psychopharmacol. 2004;19: 331–336. doi:10.1097/00004850-200411000-00003

83. Lenze EJ, Rollman BL, Shear MK, Dew MA, Pollock BG, Ciliberti C, et al. Escitalopram for Older Adults With Generalized Anxiety Disorder: A Randomized Controlled Trial. JAMA. 2009;301: 295–303. doi:10.1001/jama.2008.977

84. Panahi Y, Moghaddam BR, Sahebkar A, Nazari MA, Beiraghdar F, Karami G, et al. A randomized, double-blind, placebo-controlled trial on the efficacy and tolerability of sertraline in Iranian veterans with post-traumatic stress disorder. Psychol Med. 2011;41: 2159–2166. doi:10.1017/S0033291711000201

85. Pollack M, Mangano R, Entsuah R, Tzanis E, Simon NM, Zhang Y. A randomized controlled trial of venlafaxine ER and paroxetine in the treatment of outpatients with panic disorder. Psychopharmacology (Berl). 2007;194: 233–242. doi:10.1007/s00213-007-0821-0

86. Pollack MH, Zaninelli R, Goddard A, McCafferty JP, Bellew KM, Burnham DB, et al. Paroxetine in the treatment of generalized anxiety disorder: results of a placebo-controlled, flexible-dosage trial. J Clin Psychiatry. 2001;62: 350–357. doi:10.4088/jcp.v62n0508

87. Pollack MH, Lepola U, Koponen H, Simon NM, Worthington JJ, Emilien G, et al. A double-blind study of the efficacy of venlafaxine extended-release, paroxetine, and placebo in the treatment of panic disorder. Depress Anxiety. 2007;24: 1–14. doi:10.1002/da.20218

88. Pollack MH, Worthington JJ, Otto MW, Maki KM, Smoller JW, Manfro GG, et al. Venlafaxine for panic disorder: results from a double-blind, placebo-controlled study. Psychopharmacol Bull. 1996;32: 667–670.

89. Pollack MH, Otto MW, Worthington JJ, Manfro GG, Wolkow R. Sertraline in the treatment of panic disorder: a flexible-dose multicenter trial. Arch Gen Psychiatry. 1998;55: 1010–1016. doi:10.1001/archpsyc.55.11.1010

90. Rickels K, Mangano R, Khan A. A double-blind, placebo-controlled study of a flexible dose of venlafaxine ER in adult outpatients with generalized social anxiety disorder. J Clin Psychopharmacol. 2004;24: 488–496. doi:10.1097/01.jcp.0000138764.31106.60

91. Rynn M, Russell J, Erickson J, Detke MJ, Ball S, Dinkel J, et al. Efficacy and safety of duloxetine in the treatment of generalized anxiety disorder: a flexible-dose, progressive-titration, placebo-controlled trial. Depress Anxiety. 2008;25: 182–189. doi:10.1002/da.20271

92. Rynn MA, Siqueland L, Rickels K. Placebo-controlled trial of sertraline in the treatment of children with generalized anxiety disorder. Am J Psychiatry. 2001;158: 2008–2014. doi:10.1176/appi.ajp.158.12.2008

93. Sandmann J, Lörch B, Bandelow B, Härtter S, Winter P, Hiemke C, et al. Fluvoxamine or placebo in the treatment of panic disorder and relationship to blood concentrations of fluvoxamine. Pharmacopsychiatry. 1998;31: 117–121. doi:10.1055/s-2007-979311

94. Rickels K, Pollack MH, Sheehan DV, Haskins JT. Efficacy of extended-release venlafaxine in nondepressed outpatients with generalized anxiety disorder. Am J Psychiatry. 2000;157: 968–974. doi:10.1176/appi.ajp.157.6.968

95. Sharp DM, Power KG, Simpson RJ, Swanson V, Anstee JA. Global measures of outcome in a controlled comparison of pharmacological and psychological treatment of panic disorder and agoraphobia in primary care. Br J Gen Pract. 1997;47: 150–155.

96. Sheehan DV, Burnham DB, Iyengar MK, Perera P, Paxil CR Panic Disorder Study Group. Efficacy and tolerability of controlled-release paroxetine in the treatment of panic disorder. J Clin Psychiatry. 2005;66: 34–40.

97. Rickels K, Zaninelli R, McCafferty J, Bellew K, Iyengar M, Sheehan D. Paroxetine treatment of generalized anxiety disorder: a double-blind, placebo-controlled study. Am J Psychiatry. 2003;160: 749–756. doi:10.1176/appi.ajp.160.4.749

98. Stahl SM, Gergel I, Li D. Escitalopram in the treatment of panic disorder: a randomized, double-blind, placebo-controlled trial. J Clin Psychiatry. 2003;64: 1322–1327. doi:10.4088/jcp.v64n1107

99. Riddle MA, Scahill L, King RA, Hardin MT, Anderson GM, Ort SI, et al. Double-blind, crossover trial of fluoxetine and placebo in children and adolescents with obsessive-compulsive disorder. J Am Acad Child Adolesc Psychiatry. 1992;31: 1062–1069. doi:10.1097/00004583-199211000-00011

100. Stein MB, Pollack MH, Bystritsky A, Kelsey JE, Mangano RM. Efficacy of low and higher dose extended-release venlafaxine in generalized social anxiety disorder: a 6-month randomized controlled trial. Psychopharmacology. 2005;177: 280–288. doi:10.1007/s00213-004-1957-9

101. Stein DJ, Wreford Andersen E, Tonnoir B, Fineberg N. Escitalopram in obsessive–compulsive disorder: a randomized, placebo-controlled, paroxetine-referenced, fixed-dose, 24-week study. Curr Med Res Opin. 2007;23: 701–711. doi:10.1185/030079907X178838

102. Riddle MA, Reeve EA, Yaryura-Tobias JA, Yang HM, Claghorn JL, Gaffney G, et al. Fluvoxamine for Children and Adolescents With Obsessive-Compulsive Disorder: A Randomized, Controlled, Multicenter Trial. J Am Acad Child Adolesc Psychiatry. 2001;40: 222–229. doi:10.1097/00004583-200102000-00017

103. Stein MB, Fyer AJ, Davidson JR, Pollack MH, Wiita B. Fluvoxamine treatment of social phobia (social anxiety disorder): a double-blind, placebo-controlled study. Am J Psychiatry. 1999;156: 756–760. doi:10.1176/ajp.156.5.756

104. Stein MB, Liebowitz MR, Lydiard RB, Pitts CD, Bushnell W, Gergel I. Paroxetine treatment of generalized social phobia (social anxiety disorder): a randomized controlled trial. JAMA. 1998;280: 708–713. doi:10.1001/jama.280.8.708

105. Strawn JR, Prakash A, Zhang Q, Pangallo BA, Stroud CE, Cai N, et al. A Randomized, Placebo-Controlled Study of Duloxetine for the Treatment of Children and Adolescents With Generalized Anxiety Disorder. J Am Acad Child Adolesc Psychiatry. 2015;54: 283–293. doi:10.1016/j.jaac.2015.01.008

106. Robb AS, Cueva JE, Sporn J, Yang R, Vanderburg DG. Sertraline treatment of children and adolescents with posttraumatic stress disorder: a double-blind, placebo-controlled trial. J Child Adolesc Psychopharmacol. 2010;20: 463–471. doi:10.1089/cap.2009.0115

107. Tucker P, Potter-Kimball R, Wyatt DB, Parker DE, Burgin C, Jones DE, et al. Can physiologic assessment and side effects tease out differences in PTSD trials? A double-blind comparison of citalopram, sertraline, and placebo. Psychopharmacol Bull. 2003;37: 135–149.

108. Tucker P, Zaninelli R, Yehuda R, Ruggiero L, Dillingham K, Pitts CD. Paroxetine in the Treatment of Chronic Posttraumatic Stress Disorder: Results of a Placebo-Controlled, Flexible-Dosage Trial. J Clin Psychiatry. 2001;62: 860–868. doi:10.4088/JCP.v62n1105

109. Rolland PD, Kablinger AS, Brannon GE, Freeman AM. Treatment of Generalised Anxiety Disorder with Venlafaxine XR: A Randomised, Double-Blind Trial in Comparison with Buspirone and Placebo. Clinical Drug Investigation. 2000;19: 163–165. doi:10.2165/00044011-200019020-00009

110. Van Ameringen MA, Lane RM, Walker JR, Bowen RC, Chokka PR, Goldner EM, et al. Sertraline treatment of generalized social phobia: a 20-week, double-blind, placebo-controlled study. Am J Psychiatry. 2001;158: 275–281. doi:10.1176/appi.ajp.158.2.275

111. van der Kolk BA, Spinazzola J, Blaustein ME, Hopper JW, Hopper EK, Korn DL, et al. A Randomized Clinical Trial of Eye Movement Desensitization and Reprocessing (EMDR), Fluoxetine, and Pill Placebo in the Treatment of Posttraumatic Stress Disorder: Treatment Effects and Long-Term Maintenance. J Clin Psychiatry. 2007;68: 37–46. doi:10.4088/JCP.v68n0105

112. van Vliet IM, den Boer JA, Westenberg HGM. Psychopharmacological treatment of social phobia; a double blind placebo controlled study with fluvoxamine. Psychopharmacology. 1994;115: 128–134. doi:10.1007/BF02244762

113. Wade AG, Lepola U, Koponen HJ, Pedersen V, Pedersen T. The effect of citalopram in panic disorder. Br J Psychiatry. 1997;170: 549–553. doi:10.1192/bjp.170.6.549

114. Wagner KD, Berard R, Stein MB, Wetherhold E, Carpenter DJ, Perera P, et al. A multicenter, randomized, double-blind, placebo-controlled trial of paroxetine in children and adolescents with social anxiety disorder. Arch Gen Psychiatry. 2004;61: 1153–1162. doi:10.1001/archpsyc.61.11.1153

115. Walkup JT, Albano AM, Piacentini J, Birmaher B, Compton SN, Sherrill JT, et al. Cognitive behavioral therapy, sertraline, or a combination in childhood anxiety. N Engl J Med. 2008;359: 2753–2766. doi:10.1056/NEJMoa0804633

116. Walkup JT, Labellarte MJ, Riddle MA, Pine DS, Greenhill L, Klein R, et al. Fluvoxamine for the Treatment of Anxiety Disorders in Children and Adolescents. N Engl J Med. 2001;344: 1279–1285. doi:10.1056/NEJM200104263441703

117. Westenberg HGM, Stein DJ, Yang H, Li D, Barbato LM. A double-blind placebo-controlled study of controlled release fluvoxamine for the treatment of generalized social anxiety disorder. J Clin Psychopharmacol. 2004;24: 49–55. doi:10.1097/01.jcp.0000104906.75206.8b

118. Westenberg HG, den Boer JA. Selective monoamine uptake inhibitors and a serotonin antagonist in the treatment of panic disorder. Psychopharmacol Bull. 1989;25: 119–123.

119. Wu W-Y, Wang G, Ball SG, Desaiah D, Ang Q-Q. Duloxetine versus placebo in the treatment of patients with generalized anxiety disorder in China. Chin Med J (Engl). 2011;124: 3260–3268.

120. Zohar J, Amital D, Miodownik C, Kotler M, Bleich A, Lane RM, et al. Double-Blind Placebo-Controlled Pilot Study of Sertraline in Military Veterans With Posttraumatic Stress Disorder: J Clin Psychopharmacol. 2002;22: 190–195. doi:10.1097/00004714-200204000-00013

121. Zohar J, Judge R. Paroxetine versus clomipramine in the treatment of obsessive-compulsive disorder. OCD Paroxetine Study Investigators. Br J Psychiatry. 1996;169: 468–474. doi:10.1192/bjp.169.4.468

122. Jenike MA, Baer L, Summergrad P, Minichiello WE, Holland A, Seymour R. Sertraline in obsessive-compulsive disorder: a double-blind comparison with placebo. Am J Psychiatry. 1990;147: 923–928. doi:10.1176/ajp.147.7.923
