# Supplementary material for: Selective serotonin reuptake inhibitors, and serotonin and norepinephrine reuptake inhibitors for anxiety, obsessive-compulsive, and stress disorders: A 3-level network meta-analysis
Source: PLoS Med. 2021 Jun 10;18(6):e1003664. doi: 10.1371/journal.pmed.1003664 (PMC8224914; doi:10.1371/journal.pmed.1003664)
Supplement: S6 Appendix — (DOCX) [file pmed.1003664.s006.docx]

**S6 Appendix. Studies demographic information**

| **id** | **Country** | **Number of Sites** | **Population** | **Sampling** | **Main Disorder** | **n Placebo Arm (Baseline)** | **n Drug(s) Arm(s) (Baseline)** | **Number of Drug(s) Arms** |
| --- | --- | --- | --- | --- | --- | --- | --- | --- |
| JF10 | Unclear | Several | Adults/Elderly | Outpatients | SAD | 132 | 257 | 2 |
| JF11 | Sweden | 1 | Adults/Elderly | Outpatients | SAD | 48 | 44 | 1 |
| JF15 | Japan | 54 | Adults/Elderly | Unclear | SAD | 89 | 176 | 1 |
| JF16 | USA | 1 | Adults/Elderly | Unclear | Panic | 95 | 93 | 1 |
| JF20 | Netherlands | 2 | Adults/Elderly | Outpatients | Panic | 39 | 38 | 1 |
| JF22 | Several | 63 | Adults/Elderly | Outpatients | GAD | 139 | 407 | 3 |
| JF25 | Several | 39 | Adults/Elderly | Community | SAD | 151 | 139 | 1 |
| JF28 | USA and Canada | 20 | Adults/Elderly | Unclear | Panic | 69 | 209 | 3 |
| JF29 | Several | 112 | Adults/Elderly | Outpatients | GAD | 217 | 217 | 1 |
| JF3 | Several | 47 | Adults/Elderly | Outpatients | GAD | 140 | 151 | 1 |
| JF34 | USA | 2 | Children/Adolescents | Community | SAD | 32 | 33 | 1 |
| JF42 | Unclear | Unclear | Children/Adolescents | Mixed | More than 1 AnxDis | 37 | 37 | 1 |
| JF45 | Unclear | Several | Adults/Elderly | Community | Panic | 25 | 25 | 1 |
| JF56 | Several | 50 | Adults/Elderly | Outpatients | Panic | 180 | 181 | 1 |
| JF59 | Unclear | 14 | Adults/Elderly | Outpatients | PTSD | 93 | 94 | 1 |
| JF61 | USA | 9 | Adults/Elderly | Outpatients | GAD | 170 | 68 | 1 |
| JF7 | Several | 21 | Adults/Elderly | Outpatients | GAD | 190 | 188 | 1 |
| JF72 | Unclear | 6 | Adults/Elderly | Unclear | OCD | 44 | 43 | 1 |
| JF78 | Unclear | Unclear | Adults/Elderly | Community | PTSD | 27 | 27 | 1 |
| JF80 | USA | 50 | Adults/Elderly | Outpatients | GAD | 104 | 53 | 1 |
| JF82 | Brazil | 1 | Children/Adolescents | Unclear | More than 1 AnxDis | 11 | 10 | 1 |
| JF83 | Several | 21 | Adults/Elderly | Outpatients | GAD | 189 | 184 | 1 |
| JF87 | USA | 17 | Adults/Elderly | Outpatients | GAD | 98 | 174 | 2 |
| JF88 | USA | NA | Adults/Elderly | Outpatients | GAD | 157 | 158 | 1 |
| JF89 | USA | 23 | Adults/Elderly | Mixed | GAD | 140 | 139 | 1 |
| JF9 | Several | 42 | Adults/Elderly | Outpatients | GAD | 175 | 338 | 2 |
| JF94 | Japan | 86 | Adults/Elderly | Outpatients | SAD | 196 | 392 | 2 |
| LM10 | Netherlands | 1 | Adults/Elderly | Outpatients | Panic | 19 | 20 | 1 |
| LM23 | US | 1 | Adults/Elderly | Outpatients | PTSD | 6 | 7 | 1 |
| LM24 | US | 1 | Adults/Elderly | Outpatients | PTSD | 10 | 8 | 1 |
| LM34 | USA | 10 | Adults/Elderly | Outpatients | PTSD | 83 | 86 | 1 |
| LM37 | USA | 14 | Adults/Elderly | Outpatients | GAD | 127 | 124 | 1 |
| LM39 | USA | 21 | Children/Adolescents | Unclear | OCD | 32 | 69 | 1 |
| LM4 | Several | 56 | Adults/Elderly | Outpatients | PTSD | 168 | 161 | 1 |
| LM40 | Several | 36 | Children/Adolescents | Outpatients | OCD | 105 | 98 | 1 |
| LM42 | Spain | 1 | Adults/Elderly | Outpatients | SAD | 16 | 17 | 1 |
| LM48 | USA | 4 | Adults/Elderly | Outpatients | OCD | 78 | 78 | 1 |
| LM5 | USA | 2 | Adults/Elderly | Outpatients | SAD | 60 | 57 | 1 |
| LM50 | USA | 11 | Adults/Elderly | Outpatients | OCD | 84 | 241 | 3 |
| LM54 | USA | 42 | Adults/Elderly | Outpatients | GAD | 161 | 326 | 2 |
| LM57 | USA | 1 | Adults/Elderly | Outpatients | PTSD | 6 | 6 | 1 |
| LM59 | USA | NA | Adults/Elderly | Community | Panic | 36 | 18 | 1 |
| LM6 | USA | 12 | Adults/Elderly | Outpatients | PTSD | 108 | 100 | 1 |
| LM60 | USA | several | Adults/Elderly | Unclear | OCD | 126 | 127 | 1 |
| LM67 | USA | NA | Adults/Elderly | Outpatients | OCD | 20 | 18 | 1 |
| LM69 | USA | NA | Adults/Elderly | Outpatients | OCD | 21 | 23 | 1 |
| LM71 | Japan | 56 | Adults/Elderly | Outpatients | OCD | 94 | 94 | 1 |
| LM72 | Several | 47 | Adults/Elderly | Mixed | GAD | 128 | 125 | 1 |
| LM73 | Several | 41 | Adults/Elderly | Outpatients | SAD | 177 | 181 | 1 |
| LM74 | Germany | 57 | Adults/Elderly | Outpatients | GAD | 136 | 137 | 1 |
| LM76 | USA | 1 | Adults/Elderly | Outpatients | SAD | 6 | 6 | 1 |
| LM86 | Canada | 15 | Adults/Elderly | Outpatients | Panic | 62 | 62 | 1 |
| LM95 | China | 1 | Adults/Elderly | Outpatients | PTSD | 36 | 36 | 1 |
| MC1 | USA | 2 | Adults/Elderly | Unclear | SAD | 55 | 54 | 1 |
| MC10 | Several | NA | Adults/Elderly | Outpatients | SAD | 184 | 186 | 1 |
| MC12 | Several | 56 | Adults/Elderly | Outpatients | Panic | 168 | 175 | 1 |
| MC13 | Several | 22 | Adults/Elderly | Outpatients | SAD | 94 | 289 | 3 |
| MC14 | Several | 19 | Adults/Elderly | Mixed | SAD | 138 | 133 | 1 |
| MC15 | USA | 20 | Adults/Elderly | Outpatients | SAD | 204 | 211 | 1 |
| MC16 | USA | 2 | Children/Adolescents | Unclear | OCD | 22 | 21 | 1 |
| MC17 | USA | 26 | Adults/Elderly | Outpatients | SAD | 144 | 269 | 2 |
| MC2 | Several | 22 | Adults/Elderly | Unclear | Panic | 64 | 192 | 2 |
| MC20a | USA | 7 | Adults/Elderly | Outpatients | Panic | 44 | 127 | 1 |
| MC20b | USA | 7 | Adults/Elderly | Outpatients | Panic | 43 | 127 | 3 |
| MC22 | USA | 72 | Adults/Elderly | Unclear | GAD | 52 | 156 | 1 |
| MC25 | USA | 48 | Children/Adolescents | Outpatients | SAD | 148 | 137 | 1 |
| MC26 | USA | 3 | Children/Adolescents | Outpatients | OCD | 28 | 28 | 1 |
| MC28 | USA | 12 | Children/Adolescents | Outpatients | OCD | 95 | 92 | 1 |
| MC3 | UK | 31 | Adults/Elderly | Community | GAD | 122 | 122 | 1 |
| MC31 | USA | 59 | Adults/Elderly | Outpatients | PTSD | 186 | 365 | 2 |
| MC32 | USA | NA | Adults/Elderly | Unclear | PTSD | 27 | 25 | 1 |
| MC33 | USA | 43 | Adults/Elderly | Unclear | PTSD | 88 | 323 | 2 |
| MC34 | Several | 8 | Adults/Elderly | Unclear | PTSD | 34 | 110 | 1 |
| MC38 | USA | 64 | Adults/Elderly | Unclear | GAD | 212 | 203 | 1 |
| MC39 | Unclear | Several | Adults/Elderly | Unclear | Panic | 78 | 165 | 2 |
| MC4 | USA | 1 | Adults/Elderly | Mixed | More than 1 AnxDis | 17 | 17 | 1 |
| MC40 | Several | 9 | Adults/Elderly | Outpatients | Panic | 90 | 90 | 1 |
| MC42 | Several | 13 | Adults/Elderly | Unclear | OCD | 55 | 158 | 3 |
| MC44 | Several | 76 | Adults/Elderly | Outpatients | GAD | 101 | 113 | 1 |
| MC45 | Several | 53 | Adults/Elderly | Unclear | OCD | 100 | 390 | 3 |
| MC51 | Canada | 3 | Adults/Elderly | Outpatients | Panic | 47 | 43 | 1 |
| MC55 | Several | 33 | Adults/Elderly | Outpatients | GAD | 169 | 411 | 3 |
| MC56 | Greece | 4 | Adults/Elderly | Outpatients | GAD | 22 | 24 | 1 |
| MC6 | USA | 1 | Adults/Elderly | Mixed | GAD | 93 | 86 | 1 |
| MC62 | Iran | 1 | Adults/Elderly | Outpatients | PTSD | 35 | 35 | 1 |
| MC73 | Several | 39 | Adults/Elderly | Outpatients | Panic | 157 | 467 | 3 |
| MC77 | Several | 35 | Adults/Elderly | Outpatients | GAD | 163 | 161 | 1 |
| MC79 | Several | 71 | Adults/Elderly | Outpatients | Panic | 156 | 478 | 3 |
| MC81 | USA | 1 | Adults/Elderly | Outpatients | Panic | 12 | 13 | 1 |
| MC82 | USA | 10 | Adults/Elderly | Outpatients | Panic | 88 | 88 | 1 |
| MJ1 | Unclear | 17 | Adults/Elderly | Outpatients | SAD | 135 | 126 | 1 |
| MJ14 | USA | 27 | Adults/Elderly | Outpatients | GAD | 159 | 168 | 1 |
| MJ16 | USA | 1 | Children/Adolescents | Outpatients | GAD | 11 | 11 | 1 |
| MJ17 | Germany | 2 | Adults/Elderly | Outpatients | Panic | 23 | 23 | 1 |
| MJ2 | USA | 15 | Adults/Elderly | Outpatients | GAD | 96 | 253 | 3 |
| MJ22 | Scotland | NA | Adults/Elderly | Outpatients | Panic | 37 | 36 | 1 |
| MJ25 | United States, Canada | Several | Adults/Elderly | Unclear | Panic | 445 | 444 | 1 |
| MJ3 | USA, Canada | 50 | Adults/Elderly | Outpatients | GAD | 180 | 386 | 2 |
| MJ36 | USA | Several | Adults/Elderly | Outpatients | Panic | 119 | 247 | 2 |
| MJ4 | USA | 1 | Children/Adolescents | Outpatients | OCD | 7 | 7 | 1 |
| MJ42 | USA | 19 | Adults/Elderly | Outpatients | SAD | 134 | 261 | 2 |
| MJ44 | Canada, Finland,  France, Germany, South Africa,  Sweden | 58 | Adults/Elderly | Outpatients | OCD | 115 | 351 | 3 |
| MJ5 | USA | 17 | Children/Adolescents | Outpatients | OCD | 63 | 57 | 1 |
| MJ53 | USA | 4 | Adults/Elderly | Outpatients | SAD | 44 | 48 | 1 |
| MJ54 | USA, Canada | 13 | Adults/Elderly | Outpatients | SAD | 93 | 94 | 1 |
| MJ56 | USA, Mexico,  South Africa | 32 | Children/Adolescents | Outpatients | GAD | 137 | 135 | 1 |
| MJ6 | USA | 21 | Children/Adolescents | Outpatients | PTSD | 62 | 67 | 1 |
| MJ64 | USA | 1 | Adults/Elderly | Outpatients | PTSD | 10 | 48 | 2 |
| MJ66 | USA, Canada | 37 | Adults/Elderly | Outpatients | PTSD | 156 | 151 | 1 |
| MJ7 | USA | 1 | Adults/Elderly | Outpatients | GAD | 62 | 67 | 1 |
| MJ70 | Canada | 10 | Adults/Elderly | Outpatients | SAD | 69 | 135 | 1 |
| MJ71 | USA | NA | Adults/Elderly | Community | PTSD | 29 | 30 | 1 |
| MJ73 | Netherlands | 1 | Adults/Elderly | Outpatients | SAD | 13 | 15 | 1 |
| MJ77 | Finland, Sweden, Netherlands, UK | 22 | Adults/Elderly | Outpatients | Panic | 96 | 281 | 3 |
| MJ78 | USA, South Africa  Canda, Belgium | 38 | Children/Adolescents | Outpatients | SAD | 157 | 165 | 1 |
| MJ79 | USA | 6 | Children/Adolescents | Outpatients | More than 1 AnxDis | 76 | 133 | 1 |
| MJ80 | USA | 5 | children/Adolescents | Outpatients | more than 1 AnxDis | 65 | 63 | 1 |
| MJ84 | France, Germany,  UK, Ireland,  Netherlands,  South Africa, USA | 42 | Adults/Elderly | Outpatients | SAD | 151 | 149 | 1 |
| MJ85 | Netherlands | NA | adults/Elderly | Outpatients | Panic | 20 | 20 | 1 |
| MJ89 | China | 9 | Adults/Elderly | Outpatients | GAD | 102 | 108 | 1 |
| MJ93 | Israel | 3 | Adults/Elderly | Outpatients | PTSD | 19 | 23 | 1 |
| MJ94 | Several | Several | Adults/Elderly | Outpatients | OCD | 99 | 201 | 1 |
| MJ96 | USA | NA | Adults/Elderly | Outpatients | OCD | 9 | 10 | 1 |
| MJ97 | USA | NA | Adults/Elderly | Outpatients | OCD | 9 | 10 | 1 |
| UNG09 | Several | 27 | Adults/Elderly | Outpatients | SAD | 71 | 36 | 1 |
| UNG1 | USA | 25 | Adults/Elderly | Outpatients | GAD | 128 | 124 | 1 |
| UNG10 | Sweden | 1 | Adults/Elderly | Outpatients | SAD | 12 | 12 | 1 |
| UNG11 | USA | 15 | Adults/Elderly | Outpatients | OCD | 88 | 260 | 3 |
| UNG12 | USA | 13 | Adults/Elderly | Outpatients | OCD | 77 | 82 | 1 |
| UNG17 | Several | 16 | Adults/Elderly | Outpatients | SAD | 62 | 66 | 1 |
| UNG2 | USA | 19 | Adults/Elderly | Outpatients | GAD | 138 | 143 | 1 |
| UNG3 | Japan | 34 | Children/Adolescents | Unclear | OCD | 18 | 19 | 1 |
| UNG6 | Several | 50 | Adults/Elderly | Outpatients | GAD | 183 | 181 | 1 |
| UNG7 | USA | 32 | Adults/Elderly | Unclear | GAD | 163 | 164 | 1 |
| UNG8 | USA | 1 | Adults/Elderly | Outpatients | PTSD | 11 | 11 | 1 |
| UPD3 | USA | 1 | Adults/Elderly | Outpatients | SAD | 30 | 30 | 1 |
| UPD8 | USA | 1 | Children/Adolescents | Outpatients | GAD | 25 | 26 | 1 |
| GAD, generalized anxiety disorder; PTSD, post-traumatic stress disorder; OCD, obsessive-compulsive disorder | | | | | | | | |
