# Supplementary material for: Selective serotonin reuptake inhibitors, and serotonin and norepinephrine reuptake inhibitors for anxiety, obsessive-compulsive, and stress disorders: A 3-level network meta-analysis
Source: PLoS Med. 2021 Jun 10;18(6):e1003664. doi: 10.1371/journal.pmed.1003664 (PMC8224914; doi:10.1371/journal.pmed.1003664)
Supplement: S7 Appendix — (DOCX) [file pmed.1003664.s007.docx]

**S7 Appendix. Intervention information**

| **id** | **Drug(s)  mean dose or  min. to max. dose** | **Drug(s) Class** | **Comparator** | **Number of Visits** | **Outcome Week** | **Concomitant  use of Benzodiazepines** | **Placebo (led in) exclusion** |
| --- | --- | --- | --- | --- | --- | --- | --- |
| JF10 | venlafaxine - 192mg paroxetine - 44mg | SNRI,SSRI | placebo | 9 | 12-14 week | not informed | no |
| JF11 | paroxetine - 20 to 50mg | SSRI | placebo | 7 | 12-14 week | no | no |
| JF15 | fluvoxamine - 180mg | SSRI | placebo | 9 | 9-11 week | yes | not informed |
| JF16 | fluvoxamine - 100 to 300mg | SSRI | placebo | 9 | 6-8 week | yes | yes |
| JF20 | paroxetine - 20 to 60mg | SSRI | placebo | not informed | 12-14 week | no | yes |
| JF22 | escitalopram - 5mg escitalopram - 20mg paroxetine - 20mg | SSRI | head-to-head | 10 | 12-14 week | no | yes |
| JF25 | paroxetine - 35mg | SSRI | placebo | 8 | 12-14 week | no | yes |
| JF28 | paroxetine - 10mg paroxetine - 20mg paroxetine - 40mg | SSRI | different dose | 8 | 9-11 week | yes | yes |
| JF29 | paroxetine - 20mg | SSRI | placebo | 9 | 6-8 week | no | not informed |
| JF3 | duloxetine - 30 to 120mg | SNRI | placebo | 5 | 9-11 week | no | no |
| JF34 | fluoxetine - 10 to 40mg | SSRI | placebo | 13 | 12-14 week | not informed | not informed |
| JF42 | fluoxetine - 20mg | SSRI | placebo | 13 | 12-14 week | not informed | not informed |
| JF45 | fluvoxamine - 50 to 300mg | SSRI | placebo | not informed | 6-8 week | not informed | yes |
| JF56 | venlafaxine - 163mg | SNRI | placebo | 9 | 9-11 week | not informed | yes |
| JF59 | sertraline - 133mg | SSRI | placebo | 9 | 12-14 week | not informed | yes |
| JF61 | sertraline - 149mg | SSRI | placebo | 9 | 9-11 week | no | no |
| JF7 | sertraline - 95mg | SSRI | placebo | 7 | 12-14 week | no | yes |
| JF72 | sertraline - 50 to 200mg | SSRI | placebo | 6 | 6-8 week | yes | yes |
| JF78 | fluoxetine - 30mg | SSRI | placebo | 9 | 12-14 week | not informed | not informed |
| JF80 | escitalopram - 10 to 20mg | SSRI | placebo | not informed | 6-8 week | no | not informed |
| JF82 | fluoxetine - 35mg | SSRI | placebo | not informed | 12-14 week | not informed | not informed |
| JF83 | sertraline - 50 to 100mg | SSRI | placebo | 7 | 12-14 week | no | yes |
| JF87 | venlafaxine - 75mg venlafaxine - 150mg | SNRI | placebo | 7 | 6-8 week | no | yes |
| JF88 | escitalopram - 12mg | SSRI | placebo | 6 | 6-8 week | no | yes |
| JF89 | fluvoxamine - 174mg | SSRI | placebo | 10 | 12-14 week | not informed | yes |
| JF9 | duloxetine - 60mg duloxetine - 120mg | SNRI | different dose | 6 | 9-11 week | no | yes |
| JF94 | escitalopram - 10mg escitalopram - 20mg | SSRI | different dose | 7 | 12-14 week | not informed | not informed |
| LM10 | fluvoxamine - 75 to 150mg | SSRI | placebo | 6 | 6-8 week | not informed | no |
| LM23 | paroxetine – 12,5 to 62,5mg | SSRI | placebo | NA | 12-14 week | not informed | not informed |
| LM24 | paroxetine – 12,5 to 62,5mg | SSRI | placebo | 4 | 6-8 week | not informed | not informed |
| LM34 | sertraline - 135mg | SSRI | placebo | 8 | 12-14 week | no | yes |
| LM37 | venlafaxine - 75 to 225mg | SNRI | placebo | 12 | 12-14 week | no | no |
| LM39 | fluoxetine - 25mg | SSRI | placebo | 10 | 12-14 week | not informed | no |
| LM4 | venlafaxine - 182mg | SNRI | placebo | 8 | 21-26 week | unclear | no |
| LM40 | paroxetine - 25mg | SSRI | placebo | 7 | 9-11 week | not informed | no |
| LM42 | paroxetine - 20mg | SSRI | placebo | 4 | 6-8 week | unclear | no |
| LM48 | fluvoxamine - 230mg | SSRI | placebo | 5 | 9-11 week | yes | no |
| LM5 | fluoxetine - 44mg | SSRI | placebo | 4 | 12-14 week | not informed | no |
| LM50 | sertraline - 50mg sertraline - 100mg sertraline - 2000mg | SSRI | different dose | 8 | 12-14 week | no | yes |
| LM54 | duloxetine - 107mg venlafaxine - 184mg | SNRI | head-to-head | 7 | 9-11 week | not informed | not informed |
| LM57 | fluoxetine - 48mg | SSRI | placebo | 8 | 12-14 week | no | no |
| LM59 | fluvoxamine - 205mg | SSRI | placebo | 9 | 6-8 week | no | no |
| LM6 | sertraline - 146mg | SSRI | placebo | 9 | 12-14 week | no | yes |
| LM60 | fluvoxamine - 271mg | SSRI | placebo | 7 | 12-14 week | no | no |
| LM67 | fluvoxamine - 294mg | SSRI | placebo | 6 | 9-11 week | no | no |
| LM69 | fluoxetine - 78mg | SSRI | placebo | 6 | 9-11 week | no | no |
| LM71 | paroxetine - 40 to 50mg | SSRI | placebo | 6 | 12-14 week | not informed | no |
| LM72 | venlafaxine - 155mg | SNRI | placebo | 7 | 6-8 week | not informed | no |
| LM73 | escitalopram - 18mg | SSRI | placebo | 8 | 12-14 week | no | yes |
| LM74 | paroxetine - 20mg | SSRI | placebo | 6 | 9-11 week | no | no |
| LM76 | sertraline - 134mg | SSRI | placebo | 4 | 9-11 week | not informed | not informed |
| LM86 | sertraline - 116mg | SSRI | placebo | 8 | 12-14 week | yes | no |
| LM95 | sertraline - 135mg | SSRI | placebo | not informed | 12-14 week | not informed | not informed |
| MC1 | fluoxetine - 10 to 40mg | SSRI | placebo | 10 | 12-14 week | unclear | no |
| MC10 | paroxetine - 32mg | SSRI | placebo | 8 | 12-14 week | no | unclear |
| MC12 | venlafaxine - 188mg | SNRI | placebo | 8 | 9-11 week | no | yes |
| MC13 | paroxetine - 20mg paroxetine - 40mg paroxetine - 60mg | SSRI | different dose | 8 | 12-14 week | no | yes |
| MC14 | venlafaxine - 165mg | SNRI | placebo | 9 | 12-14 week | no | yes |
| MC15 | sertraline - 159mg | SSRI | placebo | 8 | 12-14 week | no | yes |
| MC16 | fluoxetine - 65mg | SSRI | placebo | 9 | 6-8 week | no | no |
| MC17 | venlafaxine - 202mg paroxetine - 46mg | SNRI,SSRI | head-to-head | not informed | 12-14 week | no | yes |
| MC2 | citalopram - 10 to 15mg citalopram - 20 to 40mg | SSRI | different dose | not informed | 12-14 week | yes | yes |
| MC20a | sertraline - 50 to 200mg | SSRI | different dose | 9 | 12-14 week | no | yes |
| MC20b | sertraline - 50mg sertraline - 100mg sertraline - 2000mg | SSRI | different dose | 9 | 12-14 week | no | yes |
| MC22 | duloxetine - 60mg | SNRI | head-to-head | 6 | 6-8 week | unclear | not informed |
| MC25 | venlafaxine - 142mg | SNRI | different dose | 9 | 12-14 week | no | not informed |
| MC26 | sertraline - 170mg | SSRI | placebo | 9 | 12-14 week | no | not informed |
| MC28 | sertraline - 167mg | SSRI | placebo | 8 | 12-14 week | no | yes |
| MC3 | venlafaxine - 110mg | SNRI | placebo | 6 | 6-8 week | no | no |
| MC31 | paroxetine - 20mg paroxetine - 40mg | SSRI | different dose | 7 | 12-14 week | no | yes |
| MC32 | paroxetine - 40mg | SSRI | placebo | 3 | 9-11 week | no | yes |
| MC33 | fluoxetine - 20mg fluoxetine - 40mg | SSRI | different dose | not informed | 12-14 week | not informed | no |
| MC34 | fluoxetine - 65mg | SSRI | placebo | not informed | 12-14 week | not informed | no |
| MC38 | escitalopram - 10mg | SSRI | placebo | not informed | 6-8 week | not informed | no |
| MC39 | fluoxetine - 10mg fluoxetine - 20mg | SSRI | different dose | 6 | 9-11 week | no | no |
| MC4 | citalopram - 10 to 30mg | SSRI | placebo | 7 | 6-8 week | yes | no |
| MC40 | fluoxetine - 30mg | SSRI | placebo | 7 | 12-14 week | not informed | unclear |
| MC42 | fluoxetine - 20mg fluoxetine - 40mg fluoxetine - 60mg | SSRI | different dose | 7 | 6-8 week 9-11 week | yes | yes |
| MC44 | venlafaxine - 75mg | SNRI | placebo | 6 | 6-8 week | no | no |
| MC45 | citalopram - 20mg citalopram - 40mg citalopram - 60mg | SSRI | different dose | 6 | 12-14 week | no | yes |
| MC51 | fluvoxamine - 171mg | SSRI | placebo | 9 | 6-8 week | yes | yes |
| MC55 | duloxetine - 20mg duloxetine - 90mg venlafaxine - 151mg | SNRI | head-to-head | not informed | 9-11 week | no | no |
| MC56 | venlafaxine - 75 to 150mg | SNRI | placebo | 7 | 9-11 week | no | yes |
| MC6 | escitalopram - 10 to 20mg | SSRI | placebo | 9 | 12-14 week | yes | no |
| MC62 | sertraline - 140mg | SSRI | placebo | 7 | 9-11 week | yes | not informed |
| MC73 | venlafaxine - 75mg venlafaxine - 225mg paroxetine - 40mg | SNRI,SSRI | head-to-head | 9 | 12-14 week | no | yes |
| MC77 | paroxetine - 27mg | SSRI | placebo | 4 | 6-8 week | no | yes |
| MC79 | venlafaxine - 75mg venlafaxine - 150mg paroxetine - 40mg | SNRI,SSRI | head-to-head | 9 | 12-14 week | no | yes |
| MC81 | venlafaxine - 166mg | SNRI | placebo | 9 | 6-8 week | not informed | yes |
| MC82 | sertraline - 131mg | SSRI | placebo | 8 | 9-11 week | no | yes |
| MJ1 | venlafaxine - 165mg | SNRI | placebo | 9 | 12-14 week | no | yes |
| MJ14 | duloxetine - 102mg | SNRI | placebo | 6 | 9-11 week | no | yes |
| MJ16 | sertraline - 50mg | SSRI | placebo | 10 | 9-11 week | not informed | no |
| MJ17 | fluvoxamine - 160mg | SSRI | placebo | 7 | 6-8 week | not informed | no |
| MJ2 | venlafaxine - 75mg venlafaxine - 150mg venlafaxine - 225mg | SNRI | different dose | 8 | 6-8 week | no | no |
| MJ22 | fluvoxamine - 100 to 150mg | SSRI | placebo | 8 | 12-14 week | not informed | yes |
| MJ25 | paroxetine - 50mg | SSRI | placebo | 11 | 9-11 week | no | yes |
| MJ3 | paroxetine - 20mg paroxetine - 40mg | SSRI | different dose | 7 | 6-8 week | no | yes |
| MJ36 | escitalopram - 11mg citalopram - 21mg | SSRI | head-to-head | 7 | 9-11 week | no | yes |
| MJ4 | fluoxetine - 20mg | SSRI | placebo | 3 | 6-8 week | not informed | no |
| MJ42 | venlafaxine - 72mg venlafaxine - 214mg | SNRI | different dose | 12 | 12-14 week | not informed | yes |
| MJ44 | escitalopram - 10mg escitalopram - 20mg paroxetine - 40mg | SSRI | head-to-head | 11 | 12-14 week | no | no |
| MJ5 | fluvoxamine - 165mg | SSRI | placebo | 8 | 9-11 week | no | yes |
| MJ53 | fluvoxamine - 202mg | SSRI | placebo | 9 | 12-14 week | yes | no |
| MJ54 | paroxetine - 37mg | SSRI | placebo | 8 | 12-14 week | not informed | yes |
| MJ56 | duloxetine - 54mg | SNRI | placebo | 6 | 9-11 week | no | no |
| MJ6 | sertraline - 106mg | SSRI | placebo | 6 | 9-11 week | no | no |
| MJ64 | citalopram - 36mg sertraline - 134mg | SSRI | head-to-head | 8 | 9-11 week | yes | no |
| MJ66 | paroxetine - 28mg | SSRI | placebo | 7 | 12-14 week | not informed | yes |
| MJ7 | venlafaxine - 75mg venlafaxine - 150mg | SNRI | different dose | 9 | 6-8 week | no | unclear |
| MJ70 | sertraline - 147mg | SSRI | placebo | 9 | 18-20 week | no | yes |
| MJ71 | fluoxetine - 30mg | SSRI | placebo | not informed | 6-8 week | not informed | no |
| MJ73 | fluvoxamine - 50 to 150mg | SSRI | placebo | 6 | 12-14 week | yes | no |
| MJ77 | citalopram - 10 to 15mg citalopram - 20 to 40mg citalopram - 40 to 60mg | SSRI | different dose | 9 | 6-8 week | yes | yes |
| MJ78 | paroxetine - 33mg | SSRI | placebo | 10 | 15-17 week | not informed | no |
| MJ79 | sertraline - 25 to 200mg | SSRI | placebo | 9 | 12-14 week | not informed | no |
| MJ80 | fluvoxamine - 300mg | SSRI | placebo | 8 | 6-8 week | no | no |
| MJ84 | fluvoxamine - 209mg | SSRI | placebo | 7 | 12-14 week | not informed | no |
| MJ85 | fluvoxamine - 300mg | SSRI | placebo | 7 | 6-8 week | not informed | not informed |
| MJ89 | duloxetine - 60 to 120mg | SNRI | placebo | not informed | 15-17 week | yes | no |
| MJ93 | sertraline - 120mg | SSRI | placebo | 6 | 9-11 week | yes | yes |
| MJ94 | paroxetine - 38mg | SSRI | placebo | 4 | 12-14 week | yes | yes |
| MJ96 | sertraline - 200mg | SSRI | placebo | 7 | 9-11 week | no | no |
| MJ97 | Sertraline - 200mg | SSRI | placebo | 7 | 9-11 week | no | no |
| UNG09 | paroxetine - 20 to 30mg | SSRI | placebo | not informed | 12-14 week | not informed | not informed |
| UNG1 | escitalopram - 10 to 20mg | SSRI | placebo | 6 | 6-8 week | no | yes |
| UNG10 | paroxetine - 8mg | SSRI | placebo | not informed | 12-14 week 6-8 week | not informed | not informed |
| UNG11 | paroxetine - 20mg paroxetine - 40mg paroxetine - 60mg | SSRI | placebo | 8 | 12-14 week | no | yes |
| UNG12 | paroxetine - 20 to 60mg | SSRI | placebo | not informed | 12-14 week | no | not informed |
| UNG17 | paroxetine - 8mg | SSRI | placebo | not informed | 12-14 week | not informed | not informed |
| UNG2 | escitalopram - 10 to 20mg | SSRI | Placebo | 6 | 6-8 week | no | yes |
| UNG3 | fluvoxamine - 50 to 150mg | SSRI | Placebo | 5 | 9-11 week | not informed | not informed |
| UNG6 | paroxetine - 20 to 50mg | SSRI | Placebo | 8 | 6-8 week | no | yes |
| UNG7 | paroxetine - 12 to 38mg | SSRI | Placebo | 8 | 6-8 week | no | yes |
| UNG8 | paroxetine - 42mg | SSRI | Placebo | 12 | 12-14 week | no | not informed |
| UPD3 | desvenlafaxine - 79mg | SNRI | Placebo | 8 | 12-14 week | no | no |
| UPD8 | escitalopram - 15 to 20mg | SSRI | Placebo | 6 | 12-14 week | no | not informed |
| SSRIs, selective serotonin reuptake inhibitors; SNRIs, serotonin and norepinephrine reuptake inhibitors | | | | | | | |
