# Supplementary material for: Selective serotonin reuptake inhibitors, and serotonin and norepinephrine reuptake inhibitors for anxiety, obsessive-compulsive, and stress disorders: A 3-level network meta-analysis
Source: PLoS Med. 2021 Jun 10;18(6):e1003664. doi: 10.1371/journal.pmed.1003664 (PMC8224914; doi:10.1371/journal.pmed.1003664)
Supplement: S8 Appendix — (DOCX) [file pmed.1003664.s008.docx]

**S8 Appendix. Outcomes assessment information**

| **id** | **Scale Abbreviation^a^** | **Scale^b^** | **Associated Factors** | **DSM Factors** | **Type of Analysis** | **Effect Size (yi)** | **Variance (vi)** |
| --- | --- | --- | --- | --- | --- | --- | --- |
| JF10 | LSAS | Liebowitz social anxiety scale (LSAS) | social anxiety | social anxiety | LOCF | -0.896097744888929 | 0.0543638718430562 |
| JF10 | LSAS | Liebowitz social anxiety scale (LSAS) | social anxiety | social anxiety | LOCF | -1.0622091785337 | 0.0570595968681807 |
| JF10 | SPIN | social phobia inventory (SPIN) | social anxiety | social anxiety | LOCF | -0.886115099349378 | 0.0451309430827813 |
| JF11 | BSPS | Brief Social Phobia Scale (BSPS) total score | social anxiety | social anxiety | LOCF | -1.31923006693011 | 0.109599705867586 |
| JF11 | FNE | Fear of Negative Evaluation Scale (FONE) | fear of negative evaluation | social anxiety | LOCF | -1.02837524517162 | 0.0882808067241331 |
| JF11 | LSAS | Liebowitz Social Anxiety Scale (LSAS) total score | social anxiety | social anxiety | LOCF | -1.39140574069214 | 0.103957872435007 |
| JF15 | LSAS | Liebowitz Social Anxiety Scale – Japanese Version. Total score | social anxiety | social anxiety | LOCF | -0.579099136052946 | 0.0381189720175578 |
| JF16 | CAS | Clinical Anxiety Scale (CAS) 1-6 items | anxiety (general) | GAD | LOCF | -0.373684718613133 | 0.0411059226358366 |
| JF16 | CAS_P | Estimate of Panic Attack frequency and severity (item 7 of the CAS) | panic | panic | LOCF | -1.18640405043006 | 0.0766811424809354 |
| JF16 | CGI-S | CGI-S | clinical impression (severity) | psychiatric symptoms (general) | LOCF | -1.00933663443424 | 0.0627586450654598 |
| JF16 | PAAS_PAF_WK_N | No. of full panic attacks week | panic attack (full) | panic | LOCF | -0.274175947317793 | 0.03395420085955 |
| JF16 | PAAS_PAL_WK_N | No. of limited symptom attacks week | panic attack (limited) | panic | LOCF | -0.0453216696070832 | 0.0326123759990558 |
| JF16 | PANICSS | Panic disorder severity DSM-III-R derived | panic attack | panic | LOCF | -0.802753826624984 | 0.0504146864766325 |
| JF20 | CGI-S | CGI-S | clinical impression (severity) | psychiatric symptoms (general) | LOCF | -1.42806339398136 | 0.156104371270559 |
| JF20 | HAM_A | Hamilton Rating Scale For Anxiety (HAM-A) | anxiety (general) | GAD | LOCF | -0.81494602179861 | 0.104941377899764 |
| JF20 | MSPRS_A | Marks- Sheehan Phobia Scale (MSPS)/ anxiety | anxiety (general) | GAD | LOCF | -0.618789538971025 | 0.0982220055055598 |
| JF20 | MSPRS_AGO | Marks- Sheehan Phobia Scale (MSPS)/ agoraphobia | agoraphobia | agoraphobia | LOCF | -0.786910217198722 | 0.0933422824065059 |
| JF20 | OPS | Overall Phobia score | phobia | phobia | LOCF | -1.18076179823524 | 0.125766033250536 |
| JF20 | PAAS_AA | Antecipatory anxiety score | anticipatory anxiety | GAD | LOCF | -1.23276667821622 | 0.114194493164258 |
| JF20 | PAAS_PA_WK_N | Panic frequency (mean number of panic attacks per week) | panic attack | panic | LOCF | -0.197524880314345 | 0.0835319997865033 |
| JF20 | PGI | Patient global evaluation | patient impression (general) | psychiatric symptoms (general) | LOCF | -0.979297653406917 | 0.109421987324686 |
| JF22 | HAM_A | Hamilton Rating Scale for Anxiety (HAMA) | anxiety (general) | GAD | LOCF | -0.387597104456079 | 0.187650018186041 |
| JF22 | HAM_A | Hamilton Rating Scale for Anxiety (HAMA) | anxiety (general) | GAD | LOCF | -0.281429608063417 | 0.182650139736394 |
| JF22 | HAM_A | Hamilton Rating Scale for Anxiety (HAMA) | anxiety (general) | GAD | LOCF | -0.145363921185109 | 0.179584177715256 |
| JF25 | CGI-S | CGI-S | clinical impression (severity) | psychiatric symptoms (general) | LOCF | -0.792960296177988 | 0.0318012552867151 |
| JF25 | LSAS | Liebowitz Social Anxiety Scale (LSAS) | social anxiety | social anxiety | LOCF | -0.853337315451156 | 0.0318630218740182 |
| JF25 | SADS | Social Avoidance and Distress Scalse (SADS) | social anxiety | social anxiety | LOCF | -0.634179328979538 | 0.0298975626103604 |
| JF28 | CGI-S | CGI-S | clinical impression (severity) | psychiatric symptoms (general) | LOCF | 0.0949560700020937 | 0.193811627865147 |
| JF28 | CGI-S | CGI-S | clinical impression (severity) | psychiatric symptoms (general) | LOCF | -0.188564987759888 | 0.19978439550452 |
| JF28 | CGI-S | CGI-S | clinical impression (severity) | psychiatric symptoms (general) | LOCF | -0.613252188812093 | 0.212051885612085 |
| JF28 | FQ | Fear score | fear | phobia | LOCF | -0.355057905474546 | 0.115198178816713 |
| JF28 | FQ | Fear score | fear | phobia | LOCF | -0.713629248409826 | 0.12094762891597 |
| JF28 | FQ | Fear score | fear | phobia | LOCF | -0.912001861563782 | 0.124488919151157 |
| JF28 | HAM_A | Hamilton Anxiety Rating Scale total score | anxiety (general) | GAD | LOCF | -0.102353435230698 | 0.111266174972579 |
| JF28 | HAM_A | Hamilton Anxiety Rating Scale total score | anxiety (general) | GAD | LOCF | -0.143685640684603 | 0.110590542395451 |
| JF28 | HAM_A | Hamilton Anxiety Rating Scale total score | anxiety (general) | GAD | LOCF | -0.550226365719823 | 0.116677908563761 |
| JF28 | MSPRS_AV | Marks-Sheehan Phobia Scale Avoidance score | avoidance (phobia) | phobia | LOCF | -0.194465319213568 | 0.100693393227033 |
| JF28 | MSPRS_AV | Marks-Sheehan Phobia Scale Avoidance score | avoidance (phobia) | phobia | LOCF | -0.28478983152039 | 0.100626629750824 |
| JF28 | MSPRS_AV | Marks-Sheehan Phobia Scale Avoidance score | avoidance (phobia) | phobia | LOCF | -0.464960159239507 | 0.102346446502004 |
| JF28 | PA_INT | Intensity of full panic attacks (0=completely at ease, 10=completely in panic) | panic attack (full) | panic | LOCF | -0.572295326728247 | 0.143895965054973 |
| JF28 | PA_INT | Intensity of full panic attacks (0=completely at ease, 10=completely in panic) | panic attack (full) | panic | LOCF | -0.890529833464921 | 0.15077896407188 |
| JF28 | PA_INT | Intensity of full panic attacks (0=completely at ease, 10=completely in panic) | panic attack (full) | panic | LOCF | -0.811342756280216 | 0.147023207308763 |
| JF28 | PAAS_SIPA_WK_N | Number of situational panic attacks | situational panic attack | panic | LOCF | -0.112654774394894 | 0.0893577278611116 |
| JF28 | PAAS_SPA_WK_N | Number of spontaneous panic attacks | panic attack | panic | LOCF | -0.201768491464134 | 0.0897052206179256 |
| JF28 | PAAS_SIPA_WK_N | Number of situational panic attacks | situational panic attack | panic | LOCF | -0.542395688066405 | 0.0916719679637843 |
| JF28 | PAAS_SPA_WK_N | Number of spontaneous panic attacks | panic attack | panic | LOCF | -0.094989324194495 | 0.088159587992807 |
| JF28 | PAAS_SIPA_WK_N | Number of situational panic attacks | situational panic attack | panic | LOCF | -0.183018953885395 | 0.088099521990374 |
| JF28 | PAAS_SPA_WK_N | Number of spontaneous panic attacks | panic attack | panic | LOCF | -0.0197699437446626 | 0.0872761788862923 |
| JF28 | PAAS_AA_T_P | Anticipatory anxiety (% time worrying) | anticipatory anxiety | GAD | LOCF | -0.0733761203613051 | 0.0886510027064458 |
| JF28 | PAAS_AA_T_P | Anticipatory anxiety (% time worrying) | anticipatory anxiety | GAD | LOCF | -0.260368712007974 | 0.0885667842932907 |
| JF28 | PAAS_AA_T_P | Anticipatory anxiety (% time worrying) | anticipatory anxiety | GAD | LOCF | -0.229542839775623 | 0.0877584898908462 |
| JF28 | PAAS_PA_WK_N | Total number of panic attacks (full and limited) | panic attack | panic | LOCF | -0.224492650470099 | 0.0917174337325355 |
| JF28 | PAAS_PA_WK_N | Total number of panic attacks (full and limited) | panic attack | panic | LOCF | -0.179431076203721 | 0.0903420460150124 |
| JF28 | PAAS_PA_WK_N | Total number of panic attacks (full and limited) | panic attack | panic | LOCF | -0.223865298012914 | 0.0900128682332348 |
| JF28 | PAAS_PAF_WK_N | Number of full panic attacks | panic attack (full) | panic | LOCF | -0.203872731158078 | 0.0900786053439796 |
| JF28 | PAAS_PAF_WK_N | Number of full panic attacks | panic attack (full) | panic | LOCF | -0.301896267678868 | 0.0897294882243462 |
| JF28 | PAAS_PAF_WK_N | Number of full panic attacks | panic attack (full) | panic | LOCF | -0.122965403722179 | 0.087992087670391 |
| JF29 | CGI-S | CGI-S | clinical impression (severity) | psychiatric symptoms (general) | LOCF | -0.597913849345459 | 0.0425127154810276 |
| JF29 | HAM_A | Hamilton Rating Scale for Anxiety total score | anxiety (general) | GAD | LOCF | -0.814204654695805 | 0.0615665547258891 |
| JF3 | HADS_A | HADS-A | anxiety (general) | GAD | LOCF | -0.815729743980988 | 0.0515755546649122 |
| JF3 | HAM_A | Hamilton Anxiety Rating Scale total score | anxiety (general) | GAD | LOCF | -0.83295177435592 | 0.0504750170373928 |
| JF34 | CGI-S | CGI-S | clinical impression (severity) | psychiatric symptoms (general) | LOCF | -0.335467830712653 | 0.120360693897177 |
| JF42 | PARS | Pediatric Anxiety Rating Scale (PARS) | anxiety (general)(child) | GAD | Unclear | -0.811168436727752 | 0.19062696027017 |
| JF42 | SCARED | Screen for Child Anxiety Related Emotional Disorders child-rated ( SCARED-C) | anxiety (general)(child) | GAD | Unclear | -0.377703332496096 | 0.110036987652575 |
| JF42 | SCARED_P | Screen for Child Anxiety Related Emotional Disorders parent-rated ( SCARED-P) | anxiety (general)(child) | GAD | Unclear | -0.667198379499194 | 0.137859058888984 |
| JF45 | CAS | Clinical Anxiety Scale | anxiety (general) | GAD | Unclear | -1.18093321152161 | 0.222508917293493 |
| JF45 | PASS | Panic Attack Severity Score | panic attack | panic | Unclear | -0.128709499427317 | 0.134259036509213 |
| JF56 | CAS | Covi | anxiety (general) | GAD | LOCF | -0.284485742148074 | 0.0265302112310064 |
| JF56 | CGI-S | CGI-S | clinical impression (severity) | psychiatric symptoms (general) | LOCF | 0.736489675754226 | 0.0350413260960901 |
| JF56 | FQ_F | Phobia scale fear | fear (phobia) | phobia | LOCF | -0.210084705830164 | 0.0193541062188335 |
| JF56 | FQ_AV | Phobia scale avoidance | avoidance (phobia) | phobia | LOCF | -0.178120651442753 | 0.0181512657273323 |
| JF59 | CAPS | Clinician Administred Posttraumatic Stress Disorder (PTSD) Scale Part 2 (CAPS-2) | PTSD | PTSD | LOCF | -0.581780035902446 | 0.0600086326822387 |
| JF59 | CAPS_AF | CAPS-2 Associated features | PTSD | PTSD | LOCF | -0.430924232532928 | 0.0425065356956058 |
| JF59 | CGI-S | CGI-S | clinical impression (severity) | psychiatric symptoms (general) | LOCF | -0.528512834923249 | 0.0527575839621797 |
| JF59 | DTS | Davidson PTSD scale total score | PTSD | PTSD | LOCF | -0.599701487634021 | 0.0413265961127933 |
| JF59 | IES | Impact of event Scale (IES) | PTSD | PTSD | LOCF | -0.26936006159537 | 0.0413270189140937 |
| JF61 | CGI-S | CGI-S | clinical impression (severity) | psychiatric symptoms (general) | Mixed/Hierarchical/Random | -0.534807016075285 | 0.133603683930225 |
| JF61 | HAM_A | Hamilton Anxiety Scale (HAMA) total score | anxiety (general) | GAD | Mixed/Hierarchical/Random | -0.137045277320874 | 0.236392476763627 |
| JF7 | CGI-S | CGI Severity | clinical impression (severity) | psychiatric symptoms (general) | LOCF | -1.2093049466451 | 0.0406422779202736 |
| JF7 | HADS_A | Hospital Anxiety and Depreesion scale/ Anxiety | anxiety (general) | GAD | LOCF | -0.495802169270289 | 0.0218552514401096 |
| JF7 | HAM_A | Hamilton anxiety scale | anxiety (general) | GAD | LOCF | -0.907096942581445 | 0.0399000205084083 |
| JF72 | CGI-S | CGI-S | clinical impression (severity) | psychiatric symptoms (general) | LOCF | -0.613645405759402 | 0.0788870048149041 |
| JF72 | MOCI | Maudsley Obsessive compulsive Inventory | OCD | OCD | LOCF | -0.19667102723797 | 0.070175700642075 |
| JF72 | NIMH_GOCS | NIMH Global Obsessive-Compulsive (NIMHOC) | OCD | OCD | LOCF | -0.625477245439855 | 0.0814310908902494 |
| JF72 | YBOCS | Yale-Brown Obsessive-Compulsive Scale (YBOCS) total score | OCD | OCD | LOCF | -0.52119855280954 | 0.0763287912706237 |
| JF78 | DTS | Davidson Trauma Scale (DTS) | PTSD | PTSD | LOCF | -1.16469101721478 | 0.236421907923942 |
| JF78 | DUKE | Duke Global Severity Rating for PTSD (Duke) | PTSD | PTSD | LOCF | -1.33488618844004 | 0.227015072365685 |
| JF78 | SIP | Structured Intreview for PTSD (SIP) | PTSD | PTSD | LOCF | -0.979167675818037 | 0.290729525727433 |
| JF78 | SVS | Vulnerability to the Effects of Stress (VS) | stress vulnerability | stress | LOCF | -0.902698156138078 | 0.14089909856066 |
| JF80 | CGI-S | CGI-S endpoint | clinical impression (severity) | psychiatric symptoms (general) | Mixed/Hierarchical/Random | -0.665637301078418 | 0.0898537882204157 |
| JF80 | HAM_A | Hamilton |  |  |  |  |  |
| Anxiety scale (HAM-A) total score | anxiety (general) | GAD | Mixed/Hierarchical/Random | -0.588183354363679 | 0.105647356899362 |  |  |
| JF82 | MASC | Multidimensional Anxiety Scale for Children (MASC) total score | anxiety (general)(child) | GAD | Unclear | -1.38026136930563 | 0.49619908701048 |
| JF83 | CGI-S | CGI-severity | clinical impression (severity) | psychiatric symptoms (general) | LOCF | -1.16171359788262 | 0.0411589450906771 |
| JF83 | HAM_A | Hamilton Anxiety scale (HAM-A) total score | anxiety (general) | GAD | LOCF | -0.90690783090784 | 0.0405197197025528 |
| JF87 | HAM_A | Hamilton Anxiety scale (HAM-A) total score | anxiety (general) | GAD | LOCF | -0.688665942108189 | 0.123083291465255 |
| JF87 | HAM_A | Hamilton Anxiety scale (HAM-A) total score | anxiety (general) | GAD | LOCF | -0.381620401531615 | 0.114495205280633 |
| JF88 | CAS | Covi Anxiety Scale | anxiety (general) | GAD | LOCF | -0.65700571072329 | 0.0328937747271865 |
| JF88 | CGI-S | CGI Severity score | clinical impression (severity) | psychiatric symptoms (general) | LOCF | -1.1943060234367 | 0.0516955407771696 |
| JF88 | HADS_A | Hospital Anxiety and Depression Scale (HAD) Anxiety Subscale | anxiety (general) | GAD | LOCF | -0.718555246796547 | 0.023849383270864 |
| JF88 | HAM_A | Hamilton Anxiety scale (HAM-A) total score | anxiety (general) | GAD | LOCF | -0.691346721425004 | 0.0477532968296333 |
| JF89 | CGI-S | CGI Severity of Illness | clinical impression (severity) | psychiatric symptoms (general) | LOCF | -0.846554404574765 | 0.0342681449342716 |
| JF89 | LSAS | Liebowitz Social Anxiety Scale (LSAS) | social anxiety | social anxiety | LOCF | -0.834995617665281 | 0.0292088822766303 |
| JF9 | HADS | Hospital Anxiety and Depression Scale | anxiety/depression (general) | GAD | LOCF | -0.691265815584649 | 0.0377600911901718 |
| JF9 | HADS | Hospital Anxiety and Depression Scale | anxiety/depression (general) | GAD | LOCF | -0.616335615414626 | 0.0366548505253204 |
| JF9 | HAM_A | Hamilton Anxiety Scale | anxiety (general) | GAD | LOCF | -0.701723378336957 | 0.0426217562151912 |
| JF9 | HAM_A | Hamilton Anxiety Scale | anxiety (general) | GAD | LOCF | -0.611622385053353 | 0.0412051625865182 |
| JF94 | CGI-S | CGI-S | clinical impression (severity) | psychiatric symptoms (general) | LOCF | 0.022743702824878 | 0.0344742289878599 |
| JF94 | CGI-S | CGI-S | clinical impression (severity) | psychiatric symptoms (general) | LOCF | -0.502883293274385 | 0.0387185732004275 |
| JF94 | LSAS | Liebowitz Social Anxiety Scale Japanese version (LSAS-J) | social anxiety | social anxiety | LOCF | -0.233421623090585 | 0.0361882394298545 |
| JF94 | LSAS | Liebowitz Social Anxiety Scale Japanese version (LSAS-J) | social anxiety | social anxiety | LOCF | -0.590963915422649 | 0.039500404269173 |
| LM10 | FQ | FQ (Fear Questionarie) | fear | phobia | Mixed/Hierarchical/Random | -0.334029437663238 | 0.381783676447242 |
| LM10 | STAI_STA | A-STATE (State-Anxiety Inventory) | state anxiety (general) | GAD | Mixed/Hierarchical/Random | -1.4663202343883 | 0.201522097132981 |
| LM23 | CAPS | CAPS Total | PTSD | PTSD | Completers | -4.92606466181063 | 3.39206465299058 |
| LM24 | CAPS | CAPS Total | PTSD | PTSD | Completers | -3.59830312716196 | 1.7909862795776 |
| LM34 | CAPS | CAPS-2 = Clinician-Administered PTSD Scale | PTSD | PTSD | Mixed/Hierarchical/Random | 0.0908472643721259 | 0.0417784848506463 |
| LM34 | CGI-S | CGI-S = Clinical Global Impressions-Severity of Illness Scale | clinical impression (severity) | psychiatric symptoms (general) | Mixed/Hierarchical/Random | 0.0438558883435708 | 0.0394059897818265 |
| LM34 | DESNOS | DES = Disorders of Extreme Stress–Not Otherwise Specified Scale | stress disorders | stress | Mixed/Hierarchical/Random | 0.141771189402752 | 0.0396029516228681 |
| LM34 | DTS | DTS = Davidson Trauma Scale | PTSD | PTSD | Mixed/Hierarchical/Random | -0.0269776671736612 | 0.0373834663567825 |
| LM34 | HAM_A | HAM-A = Hamilton Rating Scale for Anxiety | anxiety (general) | GAD | Mixed/Hierarchical/Random | 0.180321304688063 | 0.0399599095296002 |
| LM34 | IES | IES = Impact of Event Scale | PTSD | PTSD | Mixed/Hierarchical/Random | -0.0312929021066191 | 0.0388402654473661 |
| LM34 | MISS | MISS = Mississippi Rating Scale for Combat-Related PTSD–Civilian Trauma Version | PTSD | PTSD | Mixed/Hierarchical/Random | -0.104333957737645 | 0.0361108446909316 |
| LM37 | HAM_A | HAM-A total | anxiety (general) | GAD | Completers | -0.973759299220289 | 0.064453406622245 |
| LM39 | CGI-S | CGI-Severity | clinical impression (severity) | psychiatric symptoms (general) | LOCF | -1.10547923852035 | 0.100135498435691 |
| LM39 | COIS | OCD-impact = Obsessive Compulsive Disorder Impact Scale | OCD | OCD | LOCF | -0.32432446218064 | 0.0816422117678798 |
| LM39 | CYBOCS | CY-BOCS total | OCD (child) | OCD | LOCF | -0.739877971551536 | 0.110917635029375 |
| LM39 | NIMH_GOCS | NIMH-OCD = National Institute of Mental Health Global OCD Scale | OCD | OCD | LOCF | -1.39780601733035 | 0.111608602517328 |
| LM4 | CAPS | CAPS-SX17 (Clinician-administered PTSD Scale) TOTAL | PTSD | PTSD | LOCF | -0.649133701057857 | 0.0815058420688498 |
| LM4 | CGI-S | CGI-S | clinical impression (severity) | psychiatric symptoms (general) | LOCF | -0.590614803724663 | 0.0638728683135084 |
| LM4 | SVS | SVS (Sheehan Vulnerability to the Effects of Stress Scale) | stress vulnerability | stress | LOCF | -0.399342216268594 | 0.0263528311796011 |
| LM40 | CYBOCS | CY-BOCS total | OCD (child) | OCD | LOCF | -0.71020291155133 | 0.0506433732429119 |
| LM42 | LSAS | LSAS: liebowitz social anxiety scale | social anxiety | social anxiety | Mixed/Hierarchical/Random | -0.253617510887107 | 0.187754350387284 |
| LM48 | NIMH_GOCS | NIMH-OCD = National Institute of Mental Health Global OCD Scale | OCD | OCD | LOCF | -0.553980710910298 | 0.0432566438612353 |
| LM48 | YBOCS | Y-BOCS total | OCD | OCD | LOCF | -0.461292179918487 | 0.0434170925526235 |
| LM5 | BSPS | BSPS (Brief Social Phobia Scale) | social anxiety | social anxiety | Mixed/Hierarchical/Random | -0.577523559869115 | 0.09264131903487 |
| LM5 | CGI-S | CGI-S | clinical impression (severity) | psychiatric symptoms (general) | Mixed/Hierarchical/Random | -6.8988793022798 | 3.33078077515911 |
| LM5 | SPAI | SPAI (Social Phobia and Anxiety Inventory) | social anxiety | social anxiety | Mixed/Hierarchical/Random | -0.31320378165083 | 0.067802863571517 |
| LM50 | CGI-S | CGI-S = Clinical Global Impression severity scale | clinical impression (severity) | psychiatric symptoms (general) | Unclear | -0.266170281385921 | 0.0987701482803267 |
| LM50 | CGI-S | CGI-S = Clinical Global Impression severity scale | clinical impression (severity) | psychiatric symptoms (general) | Unclear | 0.105151791359413 | 0.0935958260009241 |
| LM50 | CGI-S | CGI-S = Clinical Global Impression severity scale | clinical impression (severity) | psychiatric symptoms (general) | Unclear | -0.266170281385921 | 0.0987701482803267 |
| LM50 | NIMH_GOCS | NIMH-OCD = National Institute of Mental Health Global OCD Scale | OCD | OCD | Unclear | -0.258136097301266 | 0.0955020536024788 |
| LM50 | NIMH_GOCS | NIMH-OCD = National Institute of Mental Health Global OCD Scale | OCD | OCD | Unclear | -0.403951841275587 | 0.097394744313816 |
| LM50 | NIMH_GOCS | NIMH-OCD = National Institute of Mental Health Global OCD Scale | OCD | OCD | Unclear | -0.611598346116329 | 0.101431344322544 |
| LM50 | YBOCS | Y-BOCS total | OCD | OCD | Unclear | -0.139113449783697 | 0.0959502132032371 |
| LM50 | YBOCS | Y-BOCS total | OCD | OCD | Unclear | -0.0561962377330943 | 0.0945522709125012 |
| LM50 | YBOCS | Y-BOCS total | OCD | OCD | Unclear | -0.82672174156386 | 0.108284196915934 |
| LM54 | HADS_A | HADS-A | anxiety (general) | GAD | LOCF | -0.960591707293092 | 0.0528710938123652 |
| LM54 | HADS_A | HADS-A | anxiety (general) | GAD | LOCF | -1.04588739825831 | 0.0533986180789742 |
| LM54 | HAM_A | HAM-A total | anxiety (general) | GAD | LOCF | -0.455603711911817 | 0.0560589081346431 |
| LM54 | HAM_A | HAM-A total | anxiety (general) | GAD | LOCF | -0.716137713375574 | 0.0588003181696513 |
| LM57 | DTS | DTS Davidson Trauma Scale | PTSD | PTSD | Unclear | 0.537144991571468 | 0.533861205539994 |
| LM57 | SIP | SIP Structured Interview for PTSD | PTSD | PTSD | Unclear | -0.00934165202732988 | 0.501316269144212 |
| LM59 | CAS | CAS = Clinical Anxiety Scale | anxiety (general) | GAD | Unclear | -1.22264105790017 | 0.226572101647125 |
| LM6 | CAPS | CAPS-2 total | PTSD | PTSD | Mixed/Hierarchical/Random | -0.405667832387109 | 0.0614108499952165 |
| LM6 | CAPS_AF | CAPS-2 Associated Features | PTSD | PTSD | Mixed/Hierarchical/Random | -0.386467949901448 | 0.0363075256866828 |
| LM6 | CGI-S | CGI-S | clinical impression (severity) | psychiatric symptoms (general) | Mixed/Hierarchical/Random | -0.186820750269397 | 0.0428464983057364 |
| LM6 | DTS | Davidson PTSD Scale total | PTSD | PTSD | Mixed/Hierarchical/Random | -0.433626562334422 | 0.0386486537324069 |
| LM6 | HAM_A | HAM-A total | anxiety (general) | GAD | Mixed/Hierarchical/Random | -0.121053648367075 | 0.0371037341651184 |
| LM6 | IES | IES total | PTSD | PTSD | Mixed/Hierarchical/Random | -0.255838489329648 | 0.0406645978532524 |
| LM60 | CGI-S | CGI-S = Clinical Global Impression severity scale | clinical impression (severity) | psychiatric symptoms (general) | LOCF | -0.351099592294512 | 0.0269748277689889 |
| LM60 | YBOCS | Y-BOCS total | OCD | OCD | LOCF | -1.1405848244277 | 0.0622345602260092 |
| LM67 | CGI | CGI | clinical impression (general) | psychiatric symptoms (general) | Unclear | -0.874319168318061 | 0.194263543895105 |
| LM67 | NIMH_GOCS | NIMH-OCD = National Institute of Mental Health Global OCD Scale | OCD | OCD | Unclear | -0.84450023859519 | 0.210106704115032 |
| LM67 | YBOCS | Y-BOCS total | OCD | OCD | Unclear | -0.895352088450134 | 0.188704887816515 |
| LM69 | CPRS_OCD | OCD scale | OCD | OCD | LOCF | 0.246242535923511 | 0.150383046609692 |
| LM69 | NIMH_GOCS | NIMH-OCD = National Institute of Mental Health Global OCD Scale | OCD | OCD | LOCF | -0.387582713536209 | 0.153139335062664 |
| LM69 | YBOCS | Y-BOCS total | OCD | OCD | LOCF | -0.469573576468501 | 0.142116810645409 |
| LM71 | YBOCS | Y-BOCS total | OCD | OCD | LOCF | -1.13290194955859 | 0.0617237984063304 |
| LM72 | CGI-S | CGI-S = Clinical Global Impression severity scale | clinical impression (severity) | psychiatric symptoms (general) | LOCF | -0.193330294161598 | 0.0396403641963503 |
| LM72 | GA_VAS | Global Anxiety VAS | anxiety (general) | GAD | LOCF | -0.165927220918203 | 0.0277448632340076 |
| LM72 | HADS_A | HADS-A | anxiety (general) | GAD | LOCF | -0.338736948143764 | 0.0363989922454104 |
| LM72 | HAM_A | HAM-A total | anxiety (general) | GAD | LOCF | -0.0969576272981456 | 0.0779721178591617 |
| LM73 | LSAS | LSAS - Liebowitz Social Anxiety Scale | social anxiety | social anxiety | LOCF | -0.336563130476382 | 0.0346512848666479 |
| LM74 | CAS | CAS - COVI Anxiety Scale | anxiety (general) | GAD | LOCF | -0.57383724195528 | 0.0788730042191649 |
| LM74 | CGI-S | CGI-S = Clinical Global Impression severity scale | clinical impression (severity) | psychiatric symptoms (general) | LOCF | -0.49730509661538 | 0.0490214936766551 |
| LM74 | HAM_A | HAM-A total | anxiety (general) | GAD | LOCF | -0.28335456057042 | 0.0560275142198966 |
| LM76 | BSPS | BSPS - Brief Social Phobia Scale | social anxiety | social anxiety | LOCF | -0.941139005461723 | 0.755503387843023 |
| LM76 | FQ | Fear Questionnaire - Total | fear | phobia | LOCF | -0.614738809367854 | 0.565820271791833 |
| LM76 | LSAS | LSAS - Liebowitz Social Anxiety Scale | social anxiety | social anxiety | LOCF | -1.5911838668432 | 0.808970593516625 |
| LM86 | ACQ | Agoraphobic Cognitions Questionarie | panic | panic | Mixed/Hierarchical/Random | -0.0425628318722365 | 0.0583112491060051 |
| LM86 | BSQ | Body Sensations Questionnaire | somatic anxiety | somatic | Mixed/Hierarchical/Random | 0.174021324744664 | 0.07135627783812 |
| LM86 | CGI-S | CGI-S = Clinical Global Impression severity scale | clinical impression (severity) | psychiatric symptoms (general) | Mixed/Hierarchical/Random | -1.55279870974033 | 0.161530717119452 |
| LM86 | MI-AAL | avoidance-alone subscale of the Mobility Inventory for Agoraphobia (MI-AAL) | avoidance (panic) | panic | Mixed/Hierarchical/Random | 0.0471875226281513 | 0.0532228394087688 |
| LM95 | CGI-S | CGI-S = Clinical Global Impression severity scale | clinical impression (severity) | psychiatric symptoms (general) | LOCF | -0.652260824999814 | 0.133559382813197 |
| LM95 | IES_R | Impact of Event Scale-Revised: total score | PTSD | PTSD | LOCF | -1.01257366043148 | 0.868515987667249 |
| MC10 | CGI-S | CGI-Severity | clinical impression (severity) | psychiatric symptoms (general) | LOCF | -0.871485991929581 | 0.0264459542648743 |
| MC10 | LSAS | Liebowitz Social Anxiety Scale (LSAS) | social anxiety | social anxiety | LOCF | -0.487699784207584 | 0.0220836086510707 |
| MC10 | SADS | Social Avoidance and Distress Scale | social anxiety | social anxiety | LOCF | -0.382783741634625 | 0.0214044226735938 |
| MC12 | CGI-S | CGI-S | clinical impression (severity) | psychiatric symptoms (general) | LOCF | -0.491037457211079 | 0.0386571850068216 |
| MC12 | FQ_F | Phobia Scale – Fear | fear (phobia) | phobia | LOCF | -0.222773886214315 | 0.0204721725141613 |
| MC12 | FQ_AV | Phobia Scale – Avoidance | avoidance (phobia) | phobia | LOCF | -0.183866672013408 | 0.0192895857627823 |
| MC12 | HAM_A | HAM-A | anxiety (general) | GAD | LOCF | -0.371719409706261 | 0.0319786746120002 |
| MC12 | PDSS | Panic Disorder Severity Scale (PDSS) | panic | panic | LOCF | -0.983935919138733 | 0.0561754489620449 |
| MC13 | CGI-S | CGI-Severity | clinical impression (severity) | psychiatric symptoms (general) | LOCF | -0.888644607980234 | 0.099728889895995 |
| MC13 | CGI-S | CGI-Severity | clinical impression (severity) | psychiatric symptoms (general) | LOCF | -0.921641798566183 | 0.0991258329837433 |
| MC13 | CGI-S | CGI-Severity | clinical impression (severity) | psychiatric symptoms (general) | LOCF | -0.714580803597877 | 0.0964491869685866 |
| MC13 | LSAS | Liebowitz Social Anxiety Scale (LSAS) | social anxiety | social anxiety | LOCF | -0.733443475950587 | 0.0799368459231838 |
| MC13 | LSAS | Liebowitz Social Anxiety Scale (LSAS) | social anxiety | social anxiety | LOCF | -0.437165714036319 | 0.0749681512922611 |
| MC13 | LSAS | Liebowitz Social Anxiety Scale (LSAS) | social anxiety | social anxiety | LOCF | -0.449402995163015 | 0.0763515504478216 |
| MC13 | SADS | Social Avoidance and Distress Scale | social anxiety | social anxiety | LOCF | -0.761284925589592 | 0.0827191001156355 |
| MC13 | SADS | Social Avoidance and Distress Scale | social anxiety | social anxiety | LOCF | -0.517129460843719 | 0.0780992072805873 |
| MC13 | SADS | Social Avoidance and Distress Scale | social anxiety | social anxiety | LOCF | -0.686404647477936 | 0.0816208107862386 |
| MC14 | CGI-S | CGI-S | clinical impression (severity) | psychiatric symptoms (general) | LOCF | -0.498981303189453 | 0.0299656567097255 |
| MC14 | LSAS | Liebowitz Social Anxiety Scale (LSAS) | social anxiety | social anxiety | LOCF | -0.885828939394371 | 0.0359755911626184 |
| MC15 | BSPS | Duke Brief Social Phobia Scale (BSPS) | social anxiety | social anxiety | LOCF | -0.55629706547761 | 0.0258706534731637 |
| MC15 | CGI-S | CGI-S | clinical impression (severity) | psychiatric symptoms (general) | LOCF | -0.56952606088662 | 0.0245537820120631 |
| MC15 | HAM_A | HAM-A | anxiety (general) | GAD | LOCF | -0.300248152761861 | 0.0151737182033477 |
| MC15 | LSAS | Liebowitz Social Anxiety Scale (LSAS) | social anxiety | social anxiety | LOCF | -0.59148067276381 | 0.0278804565651736 |
| MC16 | CGI-S | CGISeverity (CGI-S) | clinical impression (severity) | psychiatric symptoms (general) | LOCF | -0.604891340850324 | 0.204378787540087 |
| MC16 | CYBOCS | CY-BOCS | OCD (child) | OCD | LOCF | -0.921068041455426 | 0.234478225903155 |
| MC16 | NIMH_GOCS | NIMH-OC | OCD | OCD | LOCF | -0.260933451040715 | 0.198659103548488 |
| MC17 | LSAS | Liebowitz Social Anxiety Scale (LSAS) | social anxiety | social anxiety | LOCF | -0.64659276697537 | 0.0530794184275367 |
| MC17 | LSAS | Liebowitz Social Anxiety Scale (LSAS) | social anxiety | social anxiety | LOCF | -0.811029225652082 | 0.0548237270254037 |
| MC17 | SPIN | Social Phobia Inventory | social anxiety | social anxiety | LOCF | -0.768145871585536 | 0.0436527766693333 |
| MC2 | FQ | Phobia Scale | phobia | phobia | LOCF | -0.380390999422767 | 0.115810716039014 |
| MC2 | FQ | Phobia Scale | phobia | phobia | LOCF | -1.19643183020022 | 0.136189350622542 |
| MC2 | FQ | Phobia Scale | phobia | phobia | LOCF | -7.91741591784332 | 0.597336806929123 |
| MC20a | PAAS_AA_T_P | %time worrying | anticipatory anxiety | GAD | LOCF | -0.100923939280427 | 0.0466205346562094 |
| MC20a | PAAS_PA_WK_N | PAAS - panic attack | panic attack | panic | LOCF | -0.378761385663281 | 0.0466012750023587 |
| MC20a | PAAS_PAL_WK_N | PAAS - Limited symptom attacks | panic attack (limited) | panic | LOCF | -0.324247238477408 | 0.0462877783568242 |
| MC20a | PAAS_UA_WK_DN | PAAS - unexpected attack | unexpected panic attack | panic | LOCF | -0.304858305965752 | 0.0467340134206335 |
| MC20b | PAAS_PA_WK_N | PAAS - panic attack | panic attack | panic | LOCF | -0.522551728759832 | 0.14662051961541 |
| MC20b | PAAS_PA_WK_N | PAAS - panic attack | panic attack | panic | LOCF | -0.423151022606068 | 0.139204400915965 |
| MC20b | PAAS_PA_WK_N | PAAS - panic attack | panic attack | panic | LOCF | -0.121250507149682 | 0.141562228163036 |
| MC20b | PAAS_PAL_WK_N | PAAS - Limited symptom attacks | panic attack (limited) | panic | LOCF | -0.311263192155349 | 0.143932633690107 |
| MC20b | PAAS_PAL_WK_N | PAAS - Limited symptom attacks | panic attack (limited) | panic | LOCF | -0.335910734638967 | 0.137874010852535 |
| MC20b | PAAS_PAL_WK_N | PAAS - Limited symptom attacks | panic attack (limited) | panic | LOCF | -0.283579258723304 | 0.142080581300147 |
| MC20b | PAAS_UA_WK_DN | PAAS - unexpected attack | unexpected panic attack | panic | LOCF | -0.332548766866426 | 0.145587122585403 |
| MC20b | PAAS_UA_WK_DN | PAAS - unexpected attack | unexpected panic attack | panic | LOCF | -0.371890986306236 | 0.139795024444299 |
| MC20b | PAAS_UA_WK_DN | PAAS - unexpected attack | unexpected panic attack | panic | LOCF | -0.106396729938198 | 0.142174679399488 |
| MC22 | HADS_A | HAD-Anxiety Subscale | anxiety (general) | GAD | LOCF | -0.258526786952658 | 0.0601605114616971 |
| MC22 | HAM_A | HAM-A | anxiety (general) | GAD | LOCF | -0.538274520038385 | 0.165198171674461 |
| MC25 | SAS_CA | Social Anxiety Scale for Children and Adolescent (SAS-CA) | social anxiety (child) | social anxiety | LOCF | -0.581372319704758 | 0.0474840267738051 |
| MC26 | CYBOCS | CYBOCS | OCD (child) | OCD | LOCF | -0.151020239937988 | 0.1558385439182 |
| MC28 | CGI-S | CGI-S | clinical impression (severity) | psychiatric symptoms (general) | LOCF | -0.339922905154325 | 0.0422634797071146 |
| MC28 | CYBOCS | CY-BOCS | OCD (child) | OCD | LOCF | -0.700825861250215 | 0.0454088490344553 |
| MC28 | NIMH_GOCS | NIMH GOCS | OCD | OCD | LOCF | -0.499087823020904 | 0.0463462452130454 |
| MC3 | HAM_A | HAM-A | anxiety (general) | GAD | LOCF | -0.307633851572878 | 0.048116193846123 |
| MC31 | CAPS | Clinician-Administered PTSD Scale, part 2 | PTSD | PTSD | LOCF | -0.871293226979787 | 0.0541096386928722 |
| MC31 | CAPS | Clinician-Administered PTSD Scale, part 2 | PTSD | PTSD | LOCF | -0.841221989504018 | 0.0538425142862902 |
| MC32 | CAPS | Clinician Administered PTSD Scale (1-17) | PTSD | PTSD | LOCF | -0.278999173114065 | 0.147643995260599 |
| MC32 | CAPS_AF | Clinician Administered PTSD Scale - Associated Features | PTSD | PTSD | LOCF | 0.00156558065016693 | 0.124262483841618 |
| MC32 | HAM_A | HAM-A | anxiety (general) | GAD | LOCF | -0.138174104223465 | 0.13819855417039 |
| MC33 | CAPS | Clinician Administered PTSD Scale | PTSD | PTSD | LOCF | -0.535523103205238 | 0.117729273552964 |
| MC33 | CAPS | Clinician Administered PTSD Scale | PTSD | PTSD | LOCF | -0.621182041492074 | 0.119774512347 |
| MC33 | CGI-S | CGI-S | clinical impression (severity) | psychiatric symptoms (general) | LOCF | -0.524612198835786 | 0.145091714967783 |
| MC33 | CGI-S | CGI-S | clinical impression (severity) | psychiatric symptoms (general) | LOCF | -0.204658327930409 | 0.140002997179906 |
| MC33 | DTS | Davidson Trauma Scale | PTSD | PTSD | LOCF | -0.233742922398243 | 0.0726520951202202 |
| MC33 | DTS | Davidson Trauma Scale | PTSD | PTSD | LOCF | -0.130644903505671 | 0.0719708169129591 |
| MC33 | HAM_A | HAM-A | anxiety (general) | GAD | LOCF | -0.1195891730282 | 0.0661181492544698 |
| MC33 | HAM_A | HAM-A | anxiety (general) | GAD | LOCF | -0.113388659267244 | 0.0663434191979346 |
| MC33 | TOP8 | TOP-8 | PTSD | PTSD | LOCF | 0.137177792530268 | 0.134590865096738 |
| MC33 | TOP8 | TOP-8 | PTSD | PTSD | LOCF | 0.187753307522724 | 0.134343798679577 |
| MC34 | CAPS | Clinician Administered PTSD Scale | PTSD | PTSD | LOCF | -0.759826304127986 | 0.0949044378054871 |
| MC34 | CGI-S | CGI-S | clinical impression (severity) | psychiatric symptoms (general) | LOCF | -1.78103578440608 | 0.109957978400773 |
| MC34 | DTS | Davidson Trauma Scale | PTSD | PTSD | LOCF | -0.655668031222213 | 0.0637580906001894 |
| MC34 | HAM_A | HAM-A | anxiety (general) | GAD | LOCF | -0.824854613472736 | 0.0696369373439422 |
| MC34 | TOP8 | TOP-8 | PTSD | PTSD | LOCF | -0.645553534993528 | 0.111407445508915 |
| MC38 | CGI-S | CGI-S | clinical impression (severity) | psychiatric symptoms (general) | LOCF | -0.866423672792104 | 0.0475865810537489 |
| MC38 | HAM_A | HAM-A | anxiety (general) | GAD | LOCF | -0.571944765492951 | 0.0520230945354241 |
| MC39 | HAM_A | Hamilton anxiety scale score | anxiety (general) | GAD | LOCF | -0.384077351551397 | 0.0680807191851527 |
| MC39 | HAM_A | Hamilton anxiety scale score | anxiety (general) | GAD | LOCF | -1.37902921093569 | 0.0877818267245112 |
| MC39 | PAAS_PA_WK_N | Total number of panic attacks/week | panic attack | panic | LOCF | -0.105260643106419 | 0.0587399838029328 |
| MC39 | PAAS_PA_WK_N | Total number of panic attacks/week | panic attack | panic | LOCF | -0.140728569123994 | 0.0596367103399871 |
| MC4 | HAM_A | Hamilton Anxiety Rating Scale | anxiety (general) | GAD | LOCF | -0.435814112038605 | 0.413880262569642 |
| MC40 | HAM_A | HAM-A | anxiety (general) | GAD | LOCF | -0.543920688638513 | 0.0682560167918013 |
| MC40 | PAAS_PAF_WK_N | Full Panic Attacks per week | panic attack (full) | panic | LOCF | -0.0944328874263338 | 0.0400205201629529 |
| MC40 | PDSS | Panic Disorder Severity Scale (PDSS) | panic | panic | LOCF | -1.07019730843988 | 0.124790678415017 |
| MC40 | STAI_STA | State Anxiety Inventory (SAI) | state anxiety (general) | GAD | LOCF | -0.536049073426506 | 0.0425923570768259 |
| MC42 | CAS | Covi Anxiety Scale (CAS) | anxiety (general) | GAD | LOCF | -0.210484202232828 | 0.113698196214172 |
| MC42 | CAS | Covi Anxiety Scale (CAS) | anxiety (general) | GAD | LOCF | -0.0510236640920367 | 0.108507791378836 |
| MC42 | CAS | Covi Anxiety Scale (CAS) | anxiety (general) | GAD | LOCF | -0.262424355026377 | 0.112937538228438 |
| MC42 | CGI-S | CGI-S | clinical impression (severity) | psychiatric symptoms (general) | LOCF | -0.361099376901288 | 0.133015582883766 |
| MC42 | CGI-S | CGI-S | clinical impression (severity) | psychiatric symptoms (general) | LOCF | -0.206034325578929 | 0.125376620757144 |
| MC42 | CGI-S | CGI-S | clinical impression (severity) | psychiatric symptoms (general) | LOCF | -0.223392265810162 | 0.129223579249366 |
| MC42 | CPRS_OCD | CPRS-OCD subscale | OCD (child) | OCD | LOCF | -0.198009324495876 | 0.128374797757531 |
| MC42 | CPRS_OCD | CPRS-OCD subscale | OCD (child) | OCD | LOCF | -0.323967332909595 | 0.125628084529934 |
| MC42 | CPRS_OCD | CPRS-OCD subscale | OCD (child) | OCD | LOCF | -0.328140768930346 | 0.129162230181122 |
| MC42 | YBOCS | Y-BOCS | OCD | OCD | LOCF | -0.589389395262429 | 0.12774453840455 |
| MC42 | YBOCS | Y-BOCS | OCD | OCD | LOCF | -0.397794558404583 | 0.119681777699323 |
| MC42 | YBOCS | Y-BOCS | OCD | OCD | LOCF | -0.317326428860534 | 0.121798086521847 |
| MC44 | HADS_A | HADS anxiety subscale | anxiety (general) | GAD | LOCF | -0.4950942258834 | 0.0484548584120231 |
| MC44 | HAM_A | HAMA | anxiety (general) | GAD | LOCF | -0.951411332771208 | 0.0908337944630801 |
| MC45 | NIMH_GOCS | NIMH-OC | OCD | OCD | LOCF | -0.573091249535557 | 0.0739622470143186 |
| MC45 | NIMH_GOCS | NIMH-OC | OCD | OCD | LOCF | -0.821373622734619 | 0.0899650067367786 |
| MC45 | NIMH_GOCS | NIMH-OC | OCD | OCD | LOCF | -0.732789760984332 | 0.0895035109175915 |
| MC45 | YBOCS | Y-BOCS | OCD | OCD | LOCF | -0.743441896260542 | 0.0950321458731142 |
| MC45 | YBOCS | Y-BOCS | OCD | OCD | LOCF | -0.983778603425426 | 0.117433994055235 |
| MC45 | YBOCS | Y-BOCS | OCD | OCD | LOCF | -1.24447112652959 | 0.125250907661653 |
| MC51 | CAS | Clinical Anxiety Scale (CAS) | anxiety (general) | GAD | LOCF | -0.14594221041741 | 0.088482680938531 |
| MC51 | CGI-S | CGI severity | clinical impression (severity) | psychiatric symptoms (general) | LOCF | 0.479003388896702 | 0.0909886236236609 |
| MC51 | CGI_E | CGI efficacy | clinical impression (efficacy) | psychiatric symptoms (general) | LOCF | -0.487554814981119 | 0.151572672307464 |
| MC51 | PAAS_AA | Anticipatory intensity | anticipatory anxiety | GAD | LOCF | -0.227030103316042 | 0.0737279493029697 |
| MC51 | PAAS_AA_T_P | Anticipatory anxiety (%) | anticipatory anxiety | GAD | LOCF | -0.0132022462440178 | 0.0692413825911932 |
| MC51 | PAAS_PAF_WK_N | Full panic attacks/weekb | panic attack (full) | panic | LOCF | -0.227894991425983 | 0.0706884461528982 |
| MC51 | PAAS_PAL_WK_N | Limited panic attacks/week | panic attack (limited) | panic | LOCF | -0.0926199502432197 | 0.0723393736237974 |
| MC55 | HAM_A | HAMA | anxiety (general) | GAD | LOCF | -0.329024818127338 | 0.0872977108467122 |
| MC55 | HAM_A | HAMA | anxiety (general) | GAD | LOCF | -0.599891286427086 | 0.071679562060493 |
| MC55 | HAM_A | HAMA | anxiety (general) | GAD | LOCF | -0.587321015778156 | 0.070778780822593 |
| MC56 | HAM_A | HAM-A | anxiety (general) | GAD | Unclear | -2.24150593386951 | 0.502476547420537 |
| MC6 | HAM_A | HAMA | anxiety (general) | GAD | LOCF | -0.41211079312799 | 0.0450434554565327 |
| MC6 | PSWQ | PSWQ | worry (GAD) | GAD | LOCF | -0.306937304863669 | 0.0363850693412463 |
| MC62 | CGI-S | CGI-S | clinical impression (severity) | psychiatric symptoms (general) | LOCF | -0.698392985897364 | 0.112749724563892 |
| MC62 | IES_R | Event Scale – Revised (IES-R) | PTSD | PTSD | LOCF | -2.26888182854349 | 0.715692996243586 |
| MC73 | PAAS_PAF_WK_N | Full-symptom panic attacks at baseline | panic attack (full) | panic | LOCF | 0.262057841160195 | 0.0408106777635585 |
| MC73 | PAAS_PAF_WK_N | Full-symptom panic attacks at baseline | panic attack (full) | panic | LOCF | -0.0809318455487767 | 0.0408136166412 |
| MC73 | PAAS_PAF_WK_N | Full-symptom panic attacks at baseline | panic attack (full) | panic | LOCF | -0.255910534414473 | 0.0427640284836731 |
| MC73 | PDSS | PDSS | panic | panic | LOCF | -1.42644913175607 | 0.144793023657791 |
| MC77 | HADS_A | HAD - anxiety subscale | anxiety (general) | GAD | LOCF | -0.423292752997891 | 0.0252001794477692 |
| MC77 | HAM_A | HAM-A | anxiety (general) | GAD | LOCF | -1.37080021678031 | 0.0602717014214067 |
| MC79 | CGI-S | CGI-S | clinical impression (severity) | psychiatric symptoms (general) | LOCF | -0.988990328892815 | 0.104123731742637 |
| MC79 | CGI-S | CGI-S | clinical impression (severity) | psychiatric symptoms (general) | LOCF | -0.964072260059015 | 0.103424311254195 |
| MC79 | CGI-S | CGI-S | clinical impression (severity) | psychiatric symptoms (general) | LOCF | -1.0680100738446 | 0.104904108142391 |
| MC79 | FQ_F | Phobia Scale (fear) | fear (phobia) | phobia | LOCF | -0.35035638153721 | 0.0454655422785409 |
| MC79 | FQ_AV | Phobia Scale (avoidance) | avoidance (phobia) | phobia | LOCF | -0.273452821661214 | 0.0432027565564592 |
| MC81 | HAM_A | HAM-A | anxiety (general) | GAD | LOCF | -0.818136179659416 | 0.26579465779166 |
| MC81 | MSPRS_F | Marks-Sheehan Phobia Rating Scale (Fear) | fear | phobia | LOCF | -0.985279753136499 | 0.339745460782456 |
| MC81 | MSPRS_AV | Marks-Sheehan Phobia Rating Scale (Avoidance) | avoidance (phobia) | phobia | LOCF | -0.223860726463671 | 0.258270093731638 |
| MC81 | PAAS_PA_WK_N | Panic Frequecy (per week) | panic attack | panic | LOCF | 0.377374602185948 | 0.292858849863363 |
| MC81 | SCL61 | SCL-61 | psychiatric symptoms (general) | psychiatric symptoms (general) | LOCF | -0.351443722454722 | 0.25074187831987 |
| MC82 | CGI-S | CGI-S (change from baseline) | clinical impression (severity) | psychiatric symptoms (general) | LOCF | -0.644377697147378 | 0.0647607614520025 |
| MC82 | HAM_A | HAM-A | anxiety (general) | GAD | LOCF | 0.099961155583119 | 0.0801222152788349 |
| MC82 | PAAS_AA_T_P | Antecipatory Anxiety (% of time worrying) | anticipatory anxiety | GAD | LOCF | -0.00232890233112867 | 0.0340916708696623 |
| MC82 | PAAS_PAF_EB | Full Panic Attack (ratio: em point-baseline) | panic attack (full) | panic | LOCF | -0.0531182581810795 | 0.0346784711390743 |
| MC82 | PDSS | PDSS | panic | panic | LOCF | -0.164490716992607 | 0.0348270285953204 |
| MJ1 | LSAS | Liebowitz Social Anxiety Scale total | social anxiety | social anxiety | LOCF | -0.837669289416128 | 0.0394553308056188 |
| MJ14 | HADS_A | Hospital Anxiety and Depression Scale Anxiety Subscale | anxiety (general) | GAD | LOCF | -0.46323356234108 | 0.0227732576210231 |
| MJ14 | HADS_D | Hospital Anxiety and Depression Scale Depression Subscale | anxiety (general) | GAD | LOCF | -0.198618700022229 | 0.0197805785467236 |
| MJ14 | HAM_A | Hamilton Anxiety Scale total score | anxiety (general) | GAD | LOCF | -0.35033671458723 | 0.0236453238092626 |
| MJ16 | ADIS_C | Anxiety Disorders Interview Schedule for Children - Revised Severity score Rated by the child | anxiety (general)(child) | GAD | Unclear | -0.317568293680607 | 0.554261854625454 |
| MJ16 | ADIS_P | Anxiety Disorders Interview Schedule for Children - Revised Severity score Rated by the parent | anxiety (general)(child) | GAD | Unclear | -2.35812766502456 | 1.17531252223087 |
| MJ16 | HAM_A | Hamilton Anxiety Rating Scale total score | anxiety (general) | GAD | Unclear | -2.75029454844712 | 0.774801964410262 |
| MJ16 | MASC | Multidimensional Anxiety Scale for Children total score | anxiety (general)(child) | GAD | Unclear | -0.345238890484827 | 0.387730535595439 |
| MJ16 | RCMAS | Revised Children's Manifest Anxiety Scale score | anxiety (general)(child) | GAD | Unclear | -1.06829624114402 | 0.378308139001662 |
| MJ17 | PAAS_PA_WK_N | Number of panic attacks | panic attack | panic | Completers | 0.0165766413667774 | 0.135782033825143 |
| MJ2 | HAM_A | Hamilton Anxiety Scale Total | anxiety (general) | GAD | LOCF | -0.413673426430391 | 0.171267094520831 |
| MJ2 | HAM_A | Hamilton Anxiety Scale Total | anxiety (general) | GAD | LOCF | -0.872519422970301 | 0.190253150813982 |
| MJ2 | HAM_A | Hamilton Anxiety Scale Total | anxiety (general) | GAD | LOCF | -0.604823027543269 | 0.177097151362812 |
| MJ22 | HAM_A | Hamilton Anxiety Scale | anxiety (general) | GAD | LOCF | -0.660889090697237 | 0.161418980047947 |
| MJ25 | CGI-S | Clinical Global Impression Severity Scale | clinical impression (severity) | psychiatric symptoms (general) | LOCF | -0.416065969153984 | 0.0123005035132668 |
| MJ25 | HAM_A | Hamilton Rating Scale for Anxiety | anxiety (general) | GAD | LOCF | -0.313680926323419 | 0.00886875212625279 |
| MJ25 | MSPRS_F | Marks-Sheehan Phobia Scale fear total score | fear | phobia | LOCF | -0.230139342379115 | 0.00760214643170765 |
| MJ25 | MSPRS_AV | Marks-Sheehan Phobia Scale avoidance total score | avoidance (phobia) | phobia | LOCF | -0.226116187348384 | 0.00735365139524046 |
| MJ25 | PAAS_PA_WK_N | Number of full panic attacks on week 2 | panic attack | panic | LOCF | -0.0702896802139858 | 0.00708555692362359 |
| MJ3 | CGI-S | Clinical Global Impression Severity | clinical impression (severity) | psychiatric symptoms (general) | LOCF | -0.838178753230043 | 0.0616240339171956 |
| MJ3 | CGI-S | Clinical Global Impression Severity | clinical impression (severity) | psychiatric symptoms (general) | LOCF | -1.36990376769051 | 0.0684303393331604 |
| MJ3 | HADS_A | Hospital Anxiety and Depression Scale anxiety subscale | anxiety (general) | GAD | LOCF | -1.23928015345067 | 0.0426084266239782 |
| MJ3 | HADS_A | Hospital Anxiety and Depression Scale anxiety subscale | anxiety (general) | GAD | LOCF | -0.920639326655617 | 0.0384585707150832 |
| MJ3 | HAM_A | Hamilton Anxiety Scale Total | anxiety (general) | GAD | LOCF | -0.966086189040117 | 0.0907514777860236 |
| MJ3 | HAM_A | Hamilton Anxiety Scale Total | anxiety (general) | GAD | LOCF | -1.08222178020893 | 0.091217283021375 |
| MJ36 | CGI-S | Clinical Global Impression Severity of Illness scale score | clinical impression (severity) | psychiatric symptoms (general) | LOCF | -0.676875215140103 | 0.0976153080372486 |
| MJ36 | CGI-S | Clinical Global Impression Severity of Illness scale score | clinical impression (severity) | psychiatric symptoms (general) | LOCF | -0.509620878052931 | 0.0960190687207329 |
| MJ36 | HAM_A | Hamilton Anxiety Rating Scale score | anxiety (general) | GAD | LOCF | -0.247056334713087 | 0.0440114234824006 |
| MJ36 | HAM_A | Hamilton Anxiety Rating Scale score | anxiety (general) | GAD | LOCF | -0.0474701379540722 | 0.0433067359042321 |
| MJ36 | PAAS | Panic and Agoraphobia Scale score | panic | panic | LOCF | -0.535829228828397 | 0.0427822377748605 |
| MJ36 | PAAS | Panic and Agoraphobia Scale score | panic | panic | LOCF | -0.406686677991597 | 0.0424998834142318 |
| MJ36 | PAAS_PA_WK_N | Panic attacks/week | panic attack | panic | LOCF | -0.118779192827091 | 0.0381121262986934 |
| MJ36 | PAAS_PA_WK_N | Panic attacks/week | panic attack | panic | LOCF | -0.0935051946876677 | 0.0385316380502873 |
| MJ4 | CGI_OCD | CGI-OCD | OCD | OCD | LOCF | -0.760048351848986 | 0.67193533626221 |
| MJ4 | LOICV | Leyton Obsessional Inventory-Child Version Symptoms | obsession (child) | OCD | LOCF | -0.168948378227262 | 0.449857153133028 |
| MJ4 | RCMAS | Revised Children's Manifest Anxiety Scale Total | anxiety (general)(child) | GAD | LOCF | 0.0972706257365526 | 0.447714762191992 |
| MJ4 | YBOCS | Children's Yale-Brown Obsessive Compulsive Scale Total | OCD | OCD | LOCF | -1.60376312709261 | 0.804867348022844 |
| MJ42 | LSAS | Liebowitz Social Anxiety Scale | social anxiety | social anxiety | LOCF | -0.698339373067568 | 0.0501658416351428 |
| MJ42 | LSAS | Liebowitz Social Anxiety Scale | social anxiety | social anxiety | LOCF | -0.729956068412802 | 0.0507284400850915 |
| MJ42 | SPIN | Social Phobia Inventory | social anxiety | social anxiety | LOCF | -0.842234103213574 | 0.0425414900290739 |
| MJ44 | NIMH_GOCS | National Institute of Mental Health Osessive-Compulsive Scale | OCD | OCD | LOCF | -0.825155331146921 | 0.0769846504701909 |
| MJ44 | NIMH_GOCS | National Institute of Mental Health Osessive-Compulsive Scale | OCD | OCD | LOCF | -0.948013073739099 | 0.0776243423910562 |
| MJ44 | NIMH_GOCS | National Institute of Mental Health Osessive-Compulsive Scale | OCD | OCD | LOCF | -0.640874483810584 | 0.073752437051275 |
| MJ44 | YBOCS | Yale-Brown Obsessive-Compulsive Scale | OCD | OCD | LOCF | -1.09586008769188 | 0.144229509084286 |
| MJ44 | YBOCS | Yale-Brown Obsessive-Compulsive Scale | OCD | OCD | LOCF | -1.11824549659992 | 0.142582582980571 |
| MJ44 | YBOCS | Yale-Brown Obsessive-Compulsive Scale | OCD | OCD | LOCF | -0.787736270308775 | 0.135332485419693 |
| MJ5 | CGI | Clinician Global Impression Scale | clinical impression (general) | psychiatric symptoms (general) | LOCF | -0.375387270679967 | 0.0553416210696491 |
| MJ5 | CYBOCS | Children's Yale-Brown Obsessive Compulsive Scale | OCD (child) | OCD | LOCF | -0.666133572244989 | 0.0696611166716426 |
| MJ5 | NIMH_GOCS | NIMH Global Obsessive Compulsive Scale | OCD | OCD | LOCF | -0.457006504156923 | 0.0682460900585724 |
| MJ5 | PAGI | Parent Global Impression Scale | parent impression (general) | psychiatric symptoms (general) | LOCF | -0.287303610764644 | 0.0543207353927967 |
| MJ5 | PGI | Subject Global Impression Scale | patient impression (general) | psychiatric symptoms (general) | LOCF | 0.0554621280520977 | 0.05374788881016 |
| MJ53 | BSPS | Brief Social Phobia Scale | social anxiety | social anxiety | LOCF | -0.827577424016603 | 0.1061032084092 |
| MJ53 | LSAS | Liebowitz Social Anxiety Scale | social anxiety | social anxiety | LOCF | -0.837567889421583 | 0.0781649129053738 |
| MJ53 | SPIN | Social Phobia Inventory | social anxiety | social anxiety | LOCF | -0.565883724849287 | 0.0774117994407535 |
| MJ54 | LSAS | Liebowitz Social Anxiety Scale total | social anxiety | social anxiety | LOCF | -0.703833520178714 | 0.0441684441435051 |
| MJ56 | CGI-S | Clinical Global Impression Severity Scale | clinical impression (severity) | psychiatric symptoms (general) | LOCF | -0.378622645422273 | 0.0686924468745009 |
| MJ56 | PARS | Pediatric Anxiety Rating Scale Severity total score | anxiety (general)(child) | GAD | LOCF | -1.50643224432366 | 0.11177124003708 |
| MJ6 | CGI-S | Clinician Global Impression Severity | clinical impression (severity) | psychiatric symptoms (general) | LOCF | 0.656237028082903 | 0.157087944631301 |
| MJ6 | CSDC | Child Stress Disorder Checklist | PTSD | PTSD | LOCF | 0.475437963883579 | 0.078520584309376 |
| MJ6 | UCLA_PTSD | University of California at Los Angeles Post-Traumatic Stress Disorder Index for DSM IV | PTSD | PTSD | LOCF | 0.105466226832265 | 0.119842820542054 |
| MJ64 | CAPS | Clinical Administered PTSD Scale | PTSD | PTSD | LOCF | -0.223461252213768 | 1.1880345833822 |
| MJ64 | CAPS | Clinical Administered PTSD Scale | PTSD | PTSD | LOCF | 0.251751300526784 | 1.15381611845229 |
| MJ64 | IES_R | Impact of Event Scale Revised | PTSD | PTSD | LOCF | 1.10028127231664 | 1.10485064990112 |
| MJ64 | IES_R | Impact of Event Scale Revised | PTSD | PTSD | LOCF | 1.11806385138948 | 1.11301118850104 |
| MJ66 | CAPS | Clinical Administered PTSD Scale Part 2 total score | PTSD | PTSD | LOCF | -0.539354388319594 | 0.040853419444631 |
| MJ66 | DTS | Davidson Trauma Scale total score | PTSD | PTSD | LOCF | -0.485783043575366 | 0.030580077648931 |
| MJ66 | TOP8 | Treatment Outcome PTSD Scale | PTSD | PTSD | LOCF | -1.77345804597957 | 0.0313227726369623 |
| MJ70 | BSPS | Brief Social Phobia Scale total | social anxiety | social anxiety | LOCF | -0.787521708305041 | 0.0501733887244773 |
| MJ70 | CGI-S | Clinical Global Impression Severity | clinical impression (severity) | psychiatric symptoms (general) | LOCF | -1.11336305962027 | 0.0635297917474482 |
| MJ70 | FNE | Fear of negative evaluation Scale | fear of negative evaluation | social anxiety | LOCF | -0.657737696791917 | 0.0394613468134167 |
| MJ70 | FQ_SA | Marks Fear Questionnaire Social Phobia subscale | social anxiety | social anxiety | LOCF | -0.810242986009049 | 0.0382902280081083 |
| MJ70 | SADS | Social Avoidance and Distress Scale | social anxiety | social anxiety | LOCF | -0.86525202999456 | 0.0554334409535498 |
| MJ70 | SPAI_SA | Social Phobia And anxiety Inventory social phobia subscale | social anxiety | social anxiety | LOCF | -1.00202630374456 | 0.051505879565903 |
| MJ71 | CAPS | Clinician-Administered PTSD Scale total score | PTSD | PTSD | Unclear | -0.253179755422702 | 0.255590852728322 |
| MJ73 | HAM_A | Hamilton Anxiety Scale | anxiety (general) | GAD | Completers | -1.20798767216634 | 0.379519109564276 |
| MJ73 | LSAS_AV | Social Phobia Avoidance Ratings | avoidance (social anxiety) | social anxiety | Completers | 0.011060286527261 | 0.246732059361244 |
| MJ73 | SCL90 | 90 Items Symptom Checklist general symptom index | psychiatric symptoms (general) | psychiatric symptoms (general) | Completers | -0.0755021784627874 | 0.263034646496896 |
| MJ73 | SPAI | Social Phobia Anxiety Ratings | social anxiety | social anxiety | Completers | -0.915681826164285 | 0.294377709168589 |
| MJ77 | HAM_A | Hamilton Anxiety Scale total | anxiety (general) | GAD | LOCF | -0.509141437683167 | 0.106238045248742 |
| MJ77 | HAM_A | Hamilton Anxiety Scale total | anxiety (general) | GAD | LOCF | -0.634773946781073 | 0.109412284978255 |
| MJ77 | HAM_A | Hamilton Anxiety Scale total | anxiety (general) | GAD | LOCF | -0.811468891051762 | 0.115856672423625 |
| MJ78 | K_GSADS_A | Kutcher Generalized Social Anxiety Disorder Scale for Adolescents | social anxiety | social anxiety | LOCF | -0.882302643766843 | 0.0292469131009751 |
| MJ78 | LSAS_C | Liebowitz Social Anxiety Scale for Children and Adolescents total score | social anxiety (child) | social anxiety | LOCF | -0.771829112570867 | 0.0295705599246609 |
| MJ78 | SPAI | Social Phobia and Anxiety Inventory difference score | social anxiety | social anxiety | LOCF | -1.04715849965763 | 0.0278513641036695 |
| MJ78 | SPIN_C | Social Phobia and Anxiety Inventory for Children | social anxiety (child) | social anxiety | LOCF | -0.762111832225882 | 0.0270852980894693 |
| MJ79 | CGI-S | Clinical Globe Impression Severity | clinical impression (severity) | psychiatric symptoms (general) | LOCF | -1.00237423521189 | 0.083592845910612 |
| MJ79 | PARS | Pediatric Anxiety Rating Scale total score | anxiety (general)(child) | GAD | LOCF | -0.517698398392553 | 0.071579238394674 |
| MJ80 | PARS | Pediatric Anxiety Rating Scale total score | anxiety (general)(child) | GAD | LOCF | -2.86433376814367 | 0.179892442368593 |
| MJ84 | CGI-S | Clinical Global Impression - Severity | clinical impression (severity) | psychiatric symptoms (general) | LOCF | -0.568171678483078 | 0.033872761668266 |
| MJ84 | LSAS | Liebowitz Social Anxiety Scale total score | social anxiety | social anxiety | LOCF | -0.6560624820673 | 0.0387747434811701 |
| MJ85 | FQ | Fear Questionnaire | fear | phobia | Unclear | -0.736939181829259 | 0.183157326512967 |
| MJ85 | HAM_A | Hamilton Anxiety Scale | anxiety (general) | GAD | Unclear | -0.880034168592222 | 0.18871148904592 |
| MJ85 | SCL90 | 90-Item Symptom Checklist - General symptom index | psychiatric symptoms (general) | psychiatric symptoms (general) | Unclear | -1.76667272118889 | 0.228028312594824 |
| MJ85 | STAI_STA | State Trait Anxiety Inventory - State score | state anxiety (general) | GAD | Unclear | -1.43293729800134 | 0.195324965807374 |
| MJ89 | HADS_A | Hospital Anxiety and Depression Scale - Anxiety Subscale total score | anxiety (general) | GAD | LOCF | -0.638696666636714 | 0.0651215534544911 |
| MJ89 | HAM_A | Hamilton Anxiety Rating Scale total score | anxiety (general) | GAD | LOCF | -0.163136671659654 | 0.0820156278573843 |
| MJ93 | CAPS | Clinician-Administered PTSD Scale Part 2 total score | PTSD | PTSD | LOCF | -0.252461497748821 | 0.216352993083127 |
| MJ93 | CGI-S | Clinical Global Impression Severity | clinical impression (severity) | psychiatric symptoms (general) | LOCF | -0.315228943150478 | 0.171987395197936 |
| MJ94 | YBOCS | Yale-Brown Obsessive-Compulsive Scale total score | OCD | OCD | LOCF | -0.630492041094105 | 0.0345696138876144 |
| MJ96 | CGI-S | Clinical Global Impression - Severity of Ilness scale | clinical impression (severity) | psychiatric symptoms (general) | LOCF | -0.829946254844362 | 0.0774881393981623 |
| MJ96 | HADS_A | Hospital Anxiety and Depression Scale anxiety score | anxiety (general) | GAD | LOCF | -1.00508151139845 | 0.0642652584625322 |
| MJ96 | HAM_A | Hamilton Anxiety Rating Scale total score | anxiety (general) | GAD | LOCF | -0.879059112481949 | 0.114423209177242 |
| MJ97 | MOCI | Maudsley Obsessional Compulsive Questionnaire | OCD | OCD | Completers | -0.149403664411349 | 0.320246044620748 |
| MJ97 | NIMH_GOCS | NIMH obsessive-compulsive scale | OCD | OCD | Completers | -0.565352385453409 | 0.326832207584138 |
| MJ97 | YBOCS | Yale-Brown Obsessive-Compulsive Scale | OCD | OCD | Completers | -0.241055863065976 | 0.322772065930144 |
| UNG1 | CGI-S | CGI Severity score | clinical impression (severity) | psychiatric symptoms (general) | LOCF | -1.09604014745262 | 0.0624962400218862 |
| UNG1 | HAM_A | Hamilton Anxiety scale (HAM-A) total score | anxiety (general) | GAD | LOCF | -0.600914741274885 | 0.0765753764569613 |
| UNG10 | CGI-S | CGI-S | clinical impression (severity) | psychiatric symptoms (general) | LOCF | -0.14350793095119 | 0.290003174743885 |
| UNG10 | LSAS | Liebowitz Social Anxiety Scale | social anxiety | social anxiety | LOCF | -0.560683832454027 | 0.315735056964934 |
| UNG11 | CGI-S | CGI-S | clinical impression (severity) | psychiatric symptoms (general) | LOCF | 0.136474626551991 | 0.0790250078525717 |
| UNG11 | CGI-S | CGI-S | clinical impression (severity) | psychiatric symptoms (general) | LOCF | -0.249159424903963 | 0.080470723162884 |
| UNG11 | CGI-S | CGI-S | clinical impression (severity) | psychiatric symptoms (general) | LOCF | -1.00238144718774 | 0.0944677593157014 |
| UNG11 | NIMH_GOCS | NIMHOCS | OCD | OCD | LOCF | -0.0639938077937108 | 0.0795557842415643 |
| UNG11 | NIMH_GOCS | NIMHOCS | OCD | OCD | LOCF | -0.66762265766928 | 0.0849091098326264 |
| UNG11 | NIMH_GOCS | NIMHOCS | OCD | OCD | LOCF | -1.52825003254428 | 0.104977956885164 |
| UNG11 | SCL90 | SCL-90 | psychiatric symptoms (general) | psychiatric symptoms (general) | LOCF | -0.229689747345271 | 0.0694771166375422 |
| UNG11 | SCL90 | SCL-90 | psychiatric symptoms (general) | psychiatric symptoms (general) | LOCF | -0.328337084029851 | 0.0685685331600441 |
| UNG11 | SCL90 | SCL-90 | psychiatric symptoms (general) | psychiatric symptoms (general) | LOCF | -0.612751400959219 | 0.0721554134201447 |
| UNG11 | YBOCS | YBOCS-tot | OCD | OCD | LOCF | 0.358412992122462 | 0.0929292980141077 |
| UNG11 | YBOCS | YBOCS-tot | OCD | OCD | LOCF | -0.100685212146414 | 0.0952969693774826 |
| UNG11 | YBOCS | YBOCS-tot | OCD | OCD | LOCF | -1.32909922144435 | 0.12458013395512 |
| UNG12 | CGI-S | CGI severity of illness | clinical impression (severity) | psychiatric symptoms (general) | LOCF | -0.227317222480397 | 0.0446575854014239 |
| UNG12 | CGI_E | CGI efficacy index | clinical impression (efficacy) | psychiatric symptoms (general) | LOCF | -0.396623867897827 | 0.0391141475455638 |
| UNG12 | NIMH_GOCS | NIMHOCS | OCD | OCD | LOCF | -0.271504652597824 | 0.0498436714048574 |
| UNG12 | SCL90 | SCL-90 | psychiatric symptoms (general) | psychiatric symptoms (general) | LOCF | -0.208504438528707 | 0.0389508828176288 |
| UNG12 | YBOCS | YBOCS-tot | OCD | OCD | LOCF | -0.212310723821987 | 0.0517583248802304 |
| UNG17 | LSAS | Liebowitz social anxiety scale (LSAS) | social anxiety | social anxiety | LOCF | 0.0276080159182011 | 0.0517869107852734 |
| UNG2 | CGI-S | CGI Severity score | clinical impression (severity) | psychiatric symptoms (general) | LOCF | -0.590628987759938 | 0.0562788713170771 |
| UNG2 | HAM_A | Hamilton Anxiety scale (HAM-A) total score | anxiety (general) | GAD | LOCF | -0.406217244392494 | 0.0607766862860209 |
| UNG3 | CYBOCS | CYBOCS | OCD (child) | OCD | LOCF | -0.748148243969816 | 0.301890832516085 |
| UNG6 | CAS | CAS - COVI Anxiety Scale | anxiety (general) | GAD | LOCF | -0.190276512200515 | 0.0226226103031482 |
| UNG6 | CGI-S | CGI-S | clinical impression (severity) | psychiatric symptoms (general) | LOCF | -0.226883923106863 | 0.0220216206791104 |
| UNG6 | HADS | Hospital Anxiety and Depression Scale (HAD) total score | anxiety/depression (general) | GAD | LOCF | -0.275090516176263 | 0.0202208750889241 |
| UNG6 | HAM_A | HAM-A total | anxiety (general) | GAD | LOCF | -0.214914743113866 | 0.0428458125256215 |
| UNG7 | CAS | CAS - COVI Anxiety Scale | anxiety (general) | GAD | LOCF | -0.554192963475487 | 0.0411470215351861 |
| UNG7 | CGI-S | CGI-S | clinical impression (severity) | psychiatric symptoms (general) | LOCF | -0.742914696117978 | 0.0547405801456653 |
| UNG7 | HADS | Hospital Anxiety and Depression Scale (HAD) total score | anxiety/depression (general) | GAD | LOCF | -0.364634968949814 | 0.0250853784964099 |
| UNG7 | HAM_A | HAM-A total | anxiety (general) | GAD | LOCF | -0.272149712524013 | 0.0758941260167892 |
| UNG8 | CAPS | Clinician-Administered PTSD Scale Part 2 total score | PTSD | PTSD | LOCF | 0.0902240150096354 | 1.03070699335518 |
| UNG8 | DTS | Davidson Trauma Scale total score | PTSD | PTSD | LOCF | 2.03162706958249 | 0.84127379382466 |
| UNG8 | TOP8 | TOP8 | PTSD | PTSD | LOCF | 0.528015542385933 | 0.574236854836792 |
| UNG9 | LSAS | Liebowitz Social Anxiety Scale | social anxiety | social anxiety | LOCF | -0.528654104380476 | 0.132429039697646 |
| UPD3 | HAM_A | HAM-A | anxiety (general) | GAD | LOCF | -0.61730276801422 | 0.12501860515779 |
| UPD3 | LSAS | LSAS | social anxiety | social anxiety | LOCF | -0.523244235145171 | 0.225844110692154 |
| UPD8 | PARS | Pediatric Anxiety Rating Scale Severity total score | anxiety (general)(child) | GAD | LOCF | -2.4893943209734 | 0.514003236063282 |
| ^a^ Scales names’ abbreviations as standardized by review authors; ^b^ Scale names as reported in the primary studies; LSAS, Liebowitz Social Anxiety Scale; LOCF, last observation carried forward; SPIN , Social Phobia Inventory; BSPS, Brief Social Phobia Scale; FNE, Fear of Negative Evaluation Scale; CAS, Clinical Anxiety Scale; CAS_P, Clinical Anxiety Scale (panic attack frequency and severity item); GAD, generalized anxiety disorder; CGI-S, Clinical Global Impression – severity; PAAS_PAF_WK_N, Panic and Agoraphobia Scale - number of full panic attacks per week; PAAS_PAL_WK_N, Panic and Agoraphobia Scale - number of limited panic attacks per week; PANICSS, Panic disorder severity - DSM-III-R derived; HAM_A, Hamilton Rating Scale for Anxiety; MSPRS_A, Marks-Sheehan Phobia Scale – anxiety; MSPRS_AGO, Marks-Sheehan Phobia Scale – agoraphobia; OPS, Overall Phobia score; PAAS_AA, Panic and Agoraphobia Scale - anticipatory anxiety score; PAAS_PA_WK_N, Panic and Agoraphobia Scale - mean number of panic attacks per week; PGI, Patient Global Impression; SADS, Social Avoidance and Distress Scale; FQ, Fear Questionnaire; MSPRS_AV, Marks-Sheehan Phobia Scale – avoidance score; PA_INT, Intensity of full panic attacks; PAAS_SIPA_WK_N, Panic and Agoraphobia Scale - number of situational panic attacks; PAAS_SPA_WK_N, Panic and Agoraphobia Scale - number of spontaneous panic attacks; PAAS_AA_T_P, Panic and Agoraphobia Scale - anticipatory anxiety (percentage of time worrying); HADS_A, Hospital Anxiety and Depression Scale – anxiety subscale; PARS, Pediatric Anxiety Rating Scale; SCARED, Screen for Child Anxiety Related Emotional Disorders – child version; SCARED_P, Screen for Child Anxiety Related Emotional Disorders – parent version; PASS, Panic Attack Severity Score; FQ_F, Fear Questionnaire – fear score; FQ_AV, Fear Questionnaire – avoidance score; CAPS, Clinician Administered Post-Traumatic Stress Disorder Scale Part 2; CAPS_AF, Clinician Administered Post-Traumatic Stress Disorder Scale – associated features; PTSD, post-traumatic stress disorder; DTS, Davidson Trauma Scale; IES, Impact of Event Scale; MOCI, Maudsley Obsessive-Compulsive Inventory; OCD, obsessive-compulsive disorder; NIMH_GOCS, National Institute of Mental Health Global Obsessive-Compulsive Scale; YBOCS, Yale-Brown Obsessive-Compulsive Scale; DUKE, Duke Global Rating for Post-Traumatic Stress Disorder; SIP, Structured Interview for Post-Traumatic Stress Disorder; SVS, Vulnerability to Stress Scale; MASC, Multidimensional Anxiety Scale for Children; HADS, Hospital Anxiety and Depression Scale; STAI_STA, State-Trait Anxiety Inventory – state subscale; DESNOS, Disorders of Extreme Stress – Not Otherwise Specified Scale; MISS, Mississippi Rating Scale for Combat-Related Post-Traumatic Stress Disorder - civilian trauma Version; COIS, Child Obsessive-Compulsive Impact Scale; CYBOCS, Yale-Brown Obsessive-Compulsive Scale – child version; SPAI, Social Phobia and Anxiety Inventory; CGI, Clinical Global Impression; CPRS_OCD, Comprehensive Psychopathological Rating Scale – obsessive-compulsive subscale; GA_VAS, Global Anxiety Visual Analog Scale; ACQ, Agoraphobic Cognitions Questionnaire; BSQ, Body Sensations Questionnaire; MI-AAL, Mobility Inventory for Agoraphobia – avoidance subscale; IES_R, Impact of Event Scale – revised version; PDSS, Panic Disorder Severity Scale; PAAS_UA_WK_DN, Panic and Agoraphobia Scale - unexpected panic attack; SAS_CA, Social Anxiety Scale for Children and Adolescents; TOP8, Treatment-Outcome Post-Traumatic Stress Disorder Scale; CGI_E, Clinical Global Impression – efficacy; PSWQ, Penn State Worry Questionnaire; MSPRS_F, Marks-Sheehan Phobia Scale – fear score; SCL61, Symptom Checklist (61 items); PAAS_PAF_EB, Panic and Agoraphobia Scale - full panic attack (endpoint to baseline ratio); HADS_D, Hospital Anxiety and Depression Scale – depression subscale; ADIS_C, Anxiety Disorders Interview Schedule for Children – child version; ADIS_P, Anxiety Disorders Interview Schedule for Children - parent version; RCMAS, Revised Children's Manifest Anxiety Scale; PAAS, Panic and Agoraphobia Scale; CGI_OCD, Clinical Global Impression – Obsessive-Compulsive Disorder; LOICV, Leyton Obsessional Inventory - child version; PAGI, Parent Global Impression; CSDC, Child Stress Disorder Checklist; UCLA_PTSD, The University of California at Los Angeles Post-Traumatic Stress Disorder Reaction Index for DSM-IV; FQ_SA, Fear Questionnaire – social anxiety score; SPAI_SA, Social Phobia and Anxiety Inventory - social anxiety subscale; LSAS_AV, Liebowitz Social Anxiety Scale – avoidance subscale; SCL90, Symptom Checklist (90 items); K_GSADS_A, Kutcher Generalized Social Anxiety Disorder Scale for Adolescents; LSAS_C, Liebowitz Social Anxiety Scale – child version; SPIN_C, Social Phobia Inventory – child version | | | | | | | |
