# Supplementary material for: Selective serotonin reuptake inhibitors, and serotonin and norepinephrine reuptake inhibitors for anxiety, obsessive-compulsive, and stress disorders: A 3-level network meta-analysis
Source: PLoS Med. 2021 Jun 10;18(6):e1003664. doi: 10.1371/journal.pmed.1003664 (PMC8224914; doi:10.1371/journal.pmed.1003664)
Supplement: S9 Appendix — (DOCX) [file pmed.1003664.s009.docx]

| **S9 Appendix.** **Standardized mean change from baseline to endpoint for placebo, medication class and medications within the same class for primary outcome (aggregate measure of mental health related symptoms) retrieved from the included studies** | | | | | | |  |
| --- | --- | --- | --- | --- | --- | --- | --- |
| **Intervention** | **o/k (n)** | **Estimated SMC (95%CI)** | **SE** | **p value** | **τ^2^** | **Heterogeneity I^2^ (%)** |  |
| **Placebo** | 469/135  (12 474) | -1.11 (-1.22 to -1.00) | 0.06 | <.001 | 0.531 | 94.65 |  |
| **SSRIs and SNRIs** | 469/135  (17 763) | -1.70 (-1.83 to -1.57) | 0.07 | <.001 | 0.507 | 89.20 |  |
| **SSRIs** | 396/111  (12 923) | -1.65 (-1.79 to -1.50) | 0.07 | <.001 | 0.551 | 95.07 |  |
| Fluoxetine | 64/16  (1168) | -1.56 (-1.91 to -1.20) | 0.18 | <.001 | 1.01 | 96.17 |  |
| Sertraline | 98/25  (2218) | -1.53 (-1.86 to -1.19) | 0.17 | <.001 | 0.926 | 96.94 |  |
| Paroxetine | 132/36  (5122) | -1.71 (-1.96 to -1.46) | 0.13 | <.001 | 0.680 | 96.73 |  |
| Fluvoxamine | 50/19  (1067) | -1.33 (-1.55 to -1.11) | 0.11 | <.001 | 0.437 | 91.34 |  |
| Citalopram | 19/6  (1113) | -1.80 (-2.34 to -1.26) | 0.28 | <.001 | 0.632 | 95.21 |  |
| Escitalopram | 33/13  (2135) | -2.33 (-2.80 to -1.86) | 0.24 | <.001 | 0.900 | 97.18 |  |
| **SNRIs** | 77/29 (4848) | -1.87 (-2.13 to -1.60) | 0.14 | <.001 | 0.745 | 96.87 |  |
| Venlafaxine | 56/22  (3358) | -1.78 (-2.07 to -1.48) | 0.15 | <.001 | 0.829 | 97.13 |  |
| Duloxetine | 19/8  (1460) | -2.15 (-2.69 to -1.62) | 0.27 | <.001 | 0.736 | 97.15 |  |
| Desvenlafaxine | 2/1  (30) | -1.62 (-2.40 to -0.85) | 0.39 | <.001 | 0.342 | 77.31 |  |
| k, number of studies; n, sample size; o, number of outcomes; SMC, standardized mean change; SE, standard error; SSRIs, selective serotonin reuptake inhibitors; SNRIs, serotonin and norepinephrine reuptake inhibitors | | | | | | | |
