# Supplementary material for: Selective serotonin reuptake inhibitors, and serotonin and norepinephrine reuptake inhibitors for anxiety, obsessive-compulsive, and stress disorders: A 3-level network meta-analysis
Source: PLoS Med. 2021 Jun 10;18(6):e1003664. doi: 10.1371/journal.pmed.1003664 (PMC8224914; doi:10.1371/journal.pmed.1003664)
Supplement: S10 Appendix — (DOCX) [file pmed.1003664.s010.docx]

| **S10 Appendix. Direct and indirect standardized mean differences between available head-to-head medications comparisons for the primary outcome (aggregate measure of mental health related symptoms)** | | | | | | | |
| --- | --- | --- | --- | --- | --- | --- | --- |
| **Medication comparison** | **Number of trials** | **Direct SMD (95%CI)** | **p value** | **Indirect SMD (95%CI)** | | **p value** |  |
| Sertraline vs citalopram | 1 | -0.18 (-0.92 to 0.56) | 0.63 | -0.28 (-0.18 to 0.74) | | 0.23 |  |
| Paroxetine vs escitalopram | 2 | -0.23 (-0.54 to 0.08) | 0.14 | 0.01 (-0.20 to 0.22) | | 0.11 |  |
| Paroxetine vs venlafaxine | 3 | 0.17 (-0.10 to 0.43) | 0.21 | -0.07 (-0.27 to 0.13) | | 0.10 |  |
| Citalopram vs escitalopram | 1 | -0.12 (-0.30 to 0.06) | 0.20 | -0.28 (-0.70 to 0.14) | | 0.21 |  |
| Venlafaxine vs duloxetine | 2 | 0.20 (-0.06 to 0.46) | 0.13 | 0.01 (-0.23 to 0.25) | | 0.12 |  |
| SMD, standardized mean difference; CI, confidence interval | | | | |  |  |  |
