# Supplementary material for: Selective serotonin reuptake inhibitors, and serotonin and norepinephrine reuptake inhibitors for anxiety, obsessive-compulsive, and stress disorders: A 3-level network meta-analysis
Source: PLoS Med. 2021 Jun 10;18(6):e1003664. doi: 10.1371/journal.pmed.1003664 (PMC8224914; doi:10.1371/journal.pmed.1003664)
Supplement: S11 Appendix — (DOCX) [file pmed.1003664.s011.docx]

| **S11 Appendix.** **Univariate meta-regression according to medication versus placebo for each symptomatic domain in included studies** | | | | | | | | |
| --- | --- | --- | --- | --- | --- | --- | --- | --- |
|  | **o/k (n)** | **Estimated SMD (95%CI)** | **SE** | **p value** | **Test of moderators (QM)** | **p value** | **Test for residual heterogeneity (QE)** | **p value** |
| **GAD** Fluoxetine | 15/8 (900) | [Ref] | [Ref] | [Ref] | [Ref] | [Ref] | [Ref] | [Ref] |
| Sertraline | 16/10 (1196) | 0.16 (-0.21 to 0.53) | 0.19 | 0.40 | 10.3253 | 0.24 | 263.8103 | <.001 |
| Paroxetine | 27/11 (2102) | -0.01 (-0.36 to 0.35) | 0.18 | 0.98 |  |  |  |  |
| Fluvoxamine | 12/9 (333) | -0.44 (-0.86 to -0.02) | 0.22 | **0.04** |  |  |  |  |
| Citalopram | 5/3 (417) | 0.08 (-0.43 to 0.59) | 0.26 | 0.76 |  |  |  |  |
| Escitalopram | 15/10 (1330) | -0.11 (-0.48 to 0.27) | 0.19 | 0.57 |  |  |  |  |
| Venlafaxine | 21/13 (1645) | -0.10 (-0.45 to 0.26) | 0.18 | 0.60 |  |  |  |  |
| Duloxetine | 18/8 (1460) | -0.06 (-0.43 to 0.30) | 0.19 | 0.74 |  |  |  |  |
| Desvenlafaxine | 1/1 (30) | -0.11 (-1.08 to 0.85) | 0.49 | 0.82 |  |  |  |  |

|  | **o/k (n)** | **Estimated SMD (95%CI)** | **SE** | **p value** | **Test of moderators (QM)** | **p value** | **Test for residual heterogeneity (QE)** | **p value** |
| --- | --- | --- | --- | --- | --- | --- | --- | --- |
| **Social Anxiety** Fluoxetine | 2/1 (57) | [Ref] | [Ref] | [Ref] | [Ref] | [Ref] | [Ref] | [Ref] |
| Sertraline | 9/3 (352) | -0.30 (-0.85 to 0.25) | 0.28 | 0.29 | 5.7650 | 0.45 | 52.4378 | 0.38 |
| Paroxetine | 24/12 (1312) | -0.27 (-0.77 to 0.24) | 0.26 | 0.30 |  |  |  |  |
| Fluvoxamine | 8/5 (527) | -0.26 (-0.79 to 0.28) | 0.27 | 0.34 |  |  |  |  |
| Escitalopram | 3/2 (573) | 0.05 (-0.53 to 0.62) | 0.29 | 0.87 |  |  |  |  |
| Venlafaxine | 10/6 (919) | -0.33 (-0.85 to 0.19) | 0.26 | 0.21 |  |  |  |  |
| Desvenlafaxine | 1/1 (30) | -0.10 (-1.19 to 0.99) | 0.56 | 0.86 |  |  |  |  |
| **OCD** Fluoxetine | 17/5 (269) | [Ref] | [Ref] | [Ref] | [Ref] | [Ref] | [Ref] | [Ref] |
| Sertraline | 15/5 (414) | 0.05 (-0.26 to 0.36) | 0.16 | 0.75 | 8.2625 | 0.14 | 72.4962 | 0.08 |
| Paroxetine | 13/6 (854) | -0.11 (-0.41 to 0.19) | 0.15 | 0.47 |  |  |  |  |
| Fluvoxamine | 8/5 (299) | -0.21 (-0.55 to 0.12) | 0.17 | 0.21 |  |  |  |  |
| Citalopram | 6/1 (390) | -0.33 (-0.77 to 0.10) | 0.22 | 0.13 |  |  |  |  |
| Escitalopram | 4/1 (232) | -0.47 (-0.93 to 0.001) | 0.24 | 0.05 |  |  |  |  |

|  | **o/k (n)** | **Estimated SMD (95%CI)** | **SE** | **p value** | **Test of moderators (QM)** | **p value** | **Test for residual heterogeneity (QE)** | **p value** |  |
| --- | --- | --- | --- | --- | --- | --- | --- | --- | --- |
| **Panic**  Fluoxetine | 4/2 (255) | [Ref] | [Ref] | [Ref] | [Ref] | [Ref] | [Ref] | [Ref] |  |
| Sertraline | 16/4 (404) | 0.08 (-0.31 to 0.48) | 0.20 | 0.68 | 2.3882 | 0.88 | 72.3849 | 0.01 |  |
| Paroxetine | 18/4 (842) | 0.02 (-0.37 to 0.41) | 0.20 | 0.91 |  |  |  |  |  |
| Fluvoxamine | 8/4 (184) | -0.07 (-0.49 to 0.34) | 0.21 | 0.72 |  |  |  |  |  |
| Citalopram | 2/1 (119) | 0.06 (-0.47 to 0.59) | 0.27 | 0.83 |  |  |  |  |  |
| Escitalopram | 2/1 (128) | -0.02 (-0.55 to 0.52) | 0.27 | 0.95 |  |  |  |  |  |
| Venlafaxine | 5/3 (504) | -0.17 (-0.66 to 0.33) | 0.25 | 0.51 |  |  |  |  |  |
| **Specific Phobias** Paroxetine | 9/3 (691) | [Ref] | [Ref] | [Ref] | [Ref] | [Ref] | [Ref] | [Ref] |  |
| Sertraline | 1/1 (6) | -0.24 (-1.75 to 1.26) | 0.77 | 0.75 | 19.1619 | <.001 | 95.3315 | <.001 |  |
| Fluvoxamine | 2/2 (40) | -0.23 (-0.97 to 0.50) | 0.37 | 0.53 |  |  |  |  |  |
| Citalopram | 3/1 (281) | -1.06 (-1.61 to -0.52) | 0.28 | <.001 |  |  |  |  |  |
| Venlafaxine | 8/4 (527) | 0.13 (-0.13 to 0.39) | 0.13 | 0.33 |  |  |  |  |  |

|  | **o/k (n)** | **Estimated SMD (95%CI)** | **SE** | **p value** | **Test of moderators (QM)** | **p value** | **Test for residual heterogeneity (QE)** | **p value** |  | |  |
| --- | --- | --- | --- | --- | --- | --- | --- | --- | --- | --- | --- |
| **PTSD**  Fluoxetine | 15/5 (496) | [Ref] | [Ref] | [Ref] | [Ref] | [Ref] | [Ref] | [Ref] |  | |  |
| Sertraline | 19/8 (464) | 0.23 (-0.33 to 0.78) | 0.33 | 0.48 | 2.0467 | 0.73 | 113.9119 | <.001 |  | |  |
| Paroxetine | 12/6 567) | -0.14 (-0.71 to 0.54) | 0.36 | 0.69 |  |  |  |  |  | |  |
| Citalopram | 2/1 (25) | 0.76 (-0.93 to 2.45) | 0.91 | 0.41 |  |  |  |  |  | |  |
| Venlafaxine | 1/1 (161) | -0.17 (-1.21 to 0.86) | 0.63 | 0.79 |  |  |  |  |  | |  |
| k, number of studies; n, sample size; o, number of outcomes; SMD, standardized mean difference; SE, standard error; QM, Cochran’s Q test of moderators; QE, Cochran’s Q test for residual heterogeneity; GAD, generalized anxiety disorder; PTSD, post-traumatic stress disorder; OCD, obsessive-compulsive disorder | | | | | | | | |  |  | |
