# Supplementary material for: Selective serotonin reuptake inhibitors, and serotonin and norepinephrine reuptake inhibitors for anxiety, obsessive-compulsive, and stress disorders: A 3-level network meta-analysis
Source: PLoS Med. 2021 Jun 10;18(6):e1003664. doi: 10.1371/journal.pmed.1003664 (PMC8224914; doi:10.1371/journal.pmed.1003664)
Supplement: S12 Appendix — (DOCX) [file pmed.1003664.s012.docx]

| **S12 Appendix.** **Univariate meta-regressions for the primary outcome (aggregate measure of mental health related symptoms) comparing medication versus placebo** | | | | | | | | |
| --- | --- | --- | --- | --- | --- | --- | --- | --- |
|  | **o/k (n)** | **Estimated SMD (95%CI)** | **SE** | **p value** | **Test of moderators (QM)** | **p value** | **Test for residual heterogeneity (QE)** | **p value** |
| **Medication class** SSRI | 284/75 (16 151) | [Ref] | [Ref] | [Ref] | [Ref] | [Ref] | [Ref] | [Ref] |
| SNRI | 62/23 (6893) | 0.06 (-0.08 to 0.19) | 0.07 | 0.40 | 0.7067 | 0.40 | 823.1743 | <.001 |
| **Medication** Fluoxetine | 61/15 (1609) | [Ref] | [Ref] | [Ref] | [Ref] | [Ref] | [Ref] | [Ref] |
| Sertraline | 86/21 (3231) | 0.16 (-0.06 to 0.37) | 0.11 | 0.16 | 10.0751 | 0.18 | 794.6615 | <.001 |
| Paroxetine | 72/21 (6527) | -0.11 (-0.32 to 0.10) | 0.11 | 0.30 |  |  |  |  |
| Fluvoxamine | 33/12 (1733) | -0.05 (-0.30 to 0.20) | 0.13 | 0.69 |  |  |  |  |
| Citalopram | 12/3 (699) | 0.03 (-0.31 to 0.36) | 0.17 | 0.87 |  |  |  |  |
| Escitalopram | 20/7 (2352) | -0.09 (-0.35 to 0.18) | 0.14 | 0.53 |  |  |  |  |
| Venlafaxine | 48/19 (5116) | 0.04 (-0.18 to 0.25) | 0.11 | 0.73 |  |  |  |  |
| Duloxetine | 14/6 (1777) | 0.03 (-0.25 to 0.32) | 0.14 | 0.81 |  |  |  |  |

|  | **o/k (n)** | **Estimated SMD (95%CI)** | **SE** | **p value** | **Test of moderators (QM)** | **p value** | **Test for residual heterogeneity (QE)** | **p value** |
| --- | --- | --- | --- | --- | --- | --- | --- | --- |
| **Comparator** Head-to-head | 40/12 (4503) | [Ref] | [Ref] | [Ref] | [Ref] | [Ref] | [Ref] | [Ref] |
| Different dose | 98/14 (5152) | -0.03 (-0.28 to 0.22) | 0.13 | 0.81 | 0.0771 | 0.96 | 822.8887 | <.001 |
| Placebo | 208/68 (13 389) | -0.03 (-0.24 to 0.18) | 0.11 | 0.79 |  |  |  |  |
| **Equivalent dose** 1 – 1.99 | 120/44 (9011) | [Ref] | [Ref] | [Ref] | [Ref] | [Ref] | [Ref] | [Ref] |
| 2 – 2.99 | 146/47 (9520) | 0.05 (-0.04 to 0.10) | 0.05 | 0.27 | 3.5660 | 0.31 | 753.2485 | <.001 |
| 3 – 3.99 | 52/19 (3137) | 0.08 (-0.06 to 0.22) | 0.07 | 0.26 |  |  |  |  |
| >= 4 | 28/11 (1376) | -0.08 (-0.26 to 0.10) | 0.09 | 0.40 |  |  |  |  |

|  | **o/k (n)** | **Estimated SMD (95%CI)** | **SE** | **p value** | **Test of moderators (QM)** | **p value** | **Test for residual heterogeneity (QE)** | **p value** |
| --- | --- | --- | --- | --- | --- | --- | --- | --- |
| **Main diagnosis** GAD | 59/21 (6916) | [Ref] | [Ref] | [Ref] | [Ref] | [Ref] | [Ref] | [Ref] |
| Social anxiety | 58/20 (5719) | -0.02 (-0.19 to 0.15) | 0.09 | 0.82 | 20.0673 | **0.001** | 656.2081 | <.001 |
| Panic | 93/17 (4430) | 0.31 (0.13 to 0.48) | 0.09 | **<.001** |  |  |  |  |
| PTSD | 104/17 (3030) | 0.23 (0.04 to 0.41) | 0.10 | **0.02** |  |  |  |  |
| OCD | 9/2 (95) | 0.11 (-0.08 to 0.29) | 0.09 | 0.26 |  |  |  |  |
| More than 1 diagnosis | 57/21 (6916) | -0.05 (-0.63 to 0.52) | 0.29 | 0.86 |  |  |  |  |
| **Time to outcome**  12-14 weeks | 159/44 (12 061) | [Ref] | [Ref] | [Ref] | [Ref] | [Ref] | [Ref] | [Ref] |
| 6-8 weeks | 64/20 (4107) | 0.02 (-0.15 to 0.18) | 0.08 | 0.84 | 9.4382 | 0.09 | 720.8498 | <.001 |
| 9-11 weeks | 110/28 (6021) | 0.17 (0.03 to 0.31) | 0.07 | **0.02** |  |  |  |  |
| 15-17 weeks | 4/1 (322) | -0.29 (-0.79 to 0.22) | 0.26 | 0.27 |  |  |  |  |
| 18-20 weeks | 6/1 (204) | -0.28 (-0.78 to 0.22) | 0.26 | 0.27 |  |  |  |  |
| 21-26 weeks | 3/1 (329) | 0.07 (-0.48 to 0.62) | 0.28 | 0.81 |  |  |  |  |

|  |  |  |  |  |  |  |  |  |  |
| --- | --- | --- | --- | --- | --- | --- | --- | --- | --- |
|  | **o/k (n)** | **Estimated SMD (95%CI)** | **SE** | **p value** | **Test of moderators (QM)** | **p value** | **Test for residual heterogeneity (QE)** | **p value** | |
| **Sample age** Adults/Elderly | 306/80 (21 193) | [Ref] | [Ref] | [Ref] | [Ref] | [Ref] | [Ref] | [Ref] | |
| Children/Adolescents | 40/14 (1851) | -0.04 (-0.23 to 0.16) | 0.10 | 0.71 | 0.1385 | 0.71 | 816.4079 | <.001 | |
| **Sampling** Outpatients | 247/70 (17 651) | [Ref] | [Ref] | [Ref] | [Ref] | [Ref] | [Ref] | [Ref] | |
| Community | 10/5 (701) | -0.21 (-0.55 to 0.13) | 0.17 | 0.22 | 1.5568 | 0.67 | 799.3650 | <.001 | |
| Mixed | 11/4 (877) | -0.04 (-0.34 to 0.26) | 0.15 | 0.81 |  |  |  |  | |
| Unclear | 78/15 (3815) | 0.00 (-0.17 to 0.18) | 0.09 | 0.95 |  |  |  |  | |
| **Benzodiazepine use**  No | 184/51 (15 040) | [Ref] | [Ref] | [Ref] | [Ref] | [Ref] | [Ref] | [Ref] | |
| Yes | 58/11 (1598) | 0.13 (-0.07 to 0.34) | 0.10 | 0.21 | 5.7484 | 0.12 | 801.4178 | <.001 | |
| Not informed | 99/30 (5869) | -0.12 (-0.26 to 0.02) | 0.07 | 0.10 |  |  |  |  | |
| Unclear | 5/2 (537) | 0.07 (-0.36 to 0.51) | 0.22 | 0.74 |  |  |  |  | |

|  | **o/k (n)** | **Estimated SMD (95%CI)** | **SE** | **p value** | **Test of moderators (QM)** | **p value** | **Test for residual heterogeneity (QE)** | **p value** |  |
| --- | --- | --- | --- | --- | --- | --- | --- | --- | --- |
| **Placebo lead-in**  No | 105/35 (6492) | [Ref] | [Ref] | [Ref] | [Ref] | [Ref] | [Ref] | [Ref] |  |
| Yes | 200/43 (13 336) | 0.05 (-0.09 to 0.19) | 0.07 | 0.47 | 3.3673 | 0.34 | 799.5166 | <.001 |  |
| Not informed | 34/14 (2666) | -0.14 (-0.36 to 0.08) | 0.11 | 0.21 |  |  |  |  |  |
| Unclear | 7/2 (550) | 0.03 (-0.37 to 0.42) | 0.20 | 0.89 |  |  |  |  |  |
| **Publication year** | 346/94 (23 044) | 0.01 (-0.01 to 0.02) | 0.01 | 0.37 | 0.7890 | 0.37 | 818.0060 | <.001 |  |
| **Analysis** Mix/Hierarchic/Random | 22/5 (856) | [Ref] | [Ref] | [Ref] | [Ref] | [Ref] | [Ref] | [Ref] |  |
| LOCF | 291/78 (21 478) | -0.33 (-0.58 to -0.07) | 0.13 | **0.01** | 9.2512 | **0.03** | 774.5552 | <.001 |  |
| Completers | 4/2 (65) | 0.05 (-0.60 to 0.70) | 0.33 | 0.87 |  |  |  |  |  |
| Unclear | 29/9 (645) | -0.47 (-0.83 to -0.12) | 0.18 | **0.009** |  |  |  |  |  |

|  | **o/k (n)** | **Estimated SMD (95%CI)** | **SE** | **p value** | **Test of moderators (QM)** | **p value** | **Test for residual heterogeneity (QE)** | **p value** |
| --- | --- | --- | --- | --- | --- | --- | --- | --- |
| **Funding** Academic | 17/8 (415) | [Ref] | [Ref] | [Ref] | [Ref] | [Ref] | [Ref] | [Ref] |
| Governmental or non-profit | 21/7 (366) | 0.24 (-0.17 to 0.65) | 0.21 | 0.26 | 4.1129 | 0.25 | 812.6459 | <.001 |
| Industry | 273/71 (20 460) | 0.30 (0.003 to 0.60) | 0.15 | **0.047** |  |  |  |  |
| Unclear | 35/8 (1803) | 0.32 (-0.04 to 0.67) | 0.18 | 0.08 |  |  |  |  |
| o, number of outcomes; k, number of studies; n, sample size; SMD, **standardized mean difference;** SE, standard error; QM, Cochran’s Q test of moderators; QE, Cochran’s Q test for residual heterogeneity; SSRI, Selective Serotonin Reuptake Inhibitor; SNRI, Serotonin and Norepinephrine Reuptake Inhibitor; GAD, generalized anxiety disorder; PTSD, post-traumatic stress disorder; OCD, obsessive-compulsive disorder; LOCF, last observation carried forward | | | | | | | | |
