# Supplementary material for: Selective serotonin reuptake inhibitors, and serotonin and norepinephrine reuptake inhibitors for anxiety, obsessive-compulsive, and stress disorders: A 3-level network meta-analysis
Source: PLoS Med. 2021 Jun 10;18(6):e1003664. doi: 10.1371/journal.pmed.1003664 (PMC8224914; doi:10.1371/journal.pmed.1003664)
Supplement: S13 Appendix — (DOCX) [file pmed.1003664.s013.docx]

| **S13 Appendix.** **Multiple meta-regression for primary outcome (aggregate measure of mental health related symptoms) comparing medication versus placebo** | | | | | | | |
| --- | --- | --- | --- | --- | --- | --- | --- |
|  | **o/k (n)** | **Estimated SMD (95%CI)** | **SE** | **p value** | **Test of moderators (QM)** | **p value** |  |
| **Publication year** | 346/94 (23 044) | 0.03 (0.01 to 0.05) | 0.01 | **0.002** | 9.7947 | **0.002** |  |
| **Medication** Fluoxetine | 61/15 (1609) | [Ref] | [Ref] | [Ref] | [Ref] | [Ref] |  |
| Sertraline | 86/21 (3231) | 0.17 (-0.14 to 0.47) | 0.16 | 0.29 | 8.5882 | 0.28 |  |
| Paroxetine | 72/21 (6527) | -0.14 (-0.41 to 0.13) | 0.14 | 0.31 |  |  |  |
| Fluvoxamine | 33/12 (1733) | 0.05 (-0.28 to 0.38) | 0.17 | 0.77 |  |  |  |
| Citalopram | 12/3 (699) | -0.07 (-0.43 to 0.30) | 0.19 | 0.72 |  |  |  |
| Escitalopram | 20/7 (2352) | -0.14 (-0.47 to 0.20) | 0.17 | 0.42 |  |  |  |
| Venlafaxine | 48/19 (5116) | -0.03 (-0.33 to 0.28) | 0.16 | 0.87 |  |  |  |
| Duloxetine | 14/6 (1777) | -0.03 (-0.45 to 0.38) | 0.21 | 0.87 |  |  |  |
| **Comparator** Head-to-head | 40/12 (4503) | [Ref] | [Ref] | [Ref] | [Ref] | [Ref] |  |
| Different dose | 98/14 (5152) | 0.05 (-0.19 to 0.29) | 0.12 | 0.67 | 1.3188 | 0.52 |  |
| Placebo | 208/68 (13 389) | -0.05 (-0.27 to 0.18) | 0.11 | 0.69 |  |  |  |

|  | **o/k (n)** | **Estimated SMD (95%CI)** | **SE** | **p value** | **Test of moderators (QM)** | **p value** |
| --- | --- | --- | --- | --- | --- | --- |
| **Equivalent dose** 1 – 1.99 | 120/44 (9011) | [Ref] | [Ref] | [Ref] | [Ref] | [Ref] |
| 2 – 2.99 | 146/47 (9520) | -0.04 (-0.15 to 0.07) | 0.06 | 0.49 | 1.2131 | 0.75 |
| 3 – 3.99 | 52/19 (3137) | 0.00 (-0.16 to 0.16) | 0.08 | 0.99 |  |  |
| >= 4 | 28/11 (1376) | -0.10 (-0.33 to 0.12) | 0.11 | 0.37 |  |  |
| **Time to outcome** 12-14 weeks | 159/44 (12 061) | [Ref] | [Ref] | [Ref] | [Ref] | [Ref] |
| 6-8 weeks | 64/20 (4107) | 0.39 (0.16 to 0.63) | 0.12 | **<.001** | 18.3588 | **0.002** |
| 9-11 weeks | 110/28 (6021) | 0.31 (0.14 to 0.49) | 0.09 | **<.001** |  |  |
| 15-17 weeks | 4/1 (322) | -0.02 (-0.51 to 0.47) | 0.25 | 0.93 |  |  |
| 18-20 weeks | 6/1 (204) | -0.33 (-0.77 to 0.11) | 0.22 | 0.14 |  |  |
| 21-26 weeks | 3/1 (329) | -0.21 (-1.15 to 0.73) | 0.48 | 0.66 |  |  |

|  | **o/k (n)** | **Estimated SMD (95%CI)** | **SE** | **p value** | **Test of moderators (QM)** | **p value** |
| --- | --- | --- | --- | --- | --- | --- |
| **Main diagnosis** GAD | 59/21 (6916) | [Ref] | [Ref] | [Ref] | [Ref] | [Ref] |
| Social anxiety | 58/20 (5719) | 0.32 (0.07 to 0.56) | 0.13 | **0.01** | 17.0862 | **0.004** |
| Panic | 93/17 (4430) | 0.42 (0.20 to 0.64) | 0.11 | **<.001** |  |  |
| PTSD | 135/17 (2854) | 0.46 (0.21 to 0.72) | 0.13 | **<.001** |  |  |
| OCD | 104/17 (3030) | 0.40 (0.11 to 0.69) | 0.15 | **0.006** |  |  |
| More than 1 diagnosis | 9/2 (95) | 0.84 (0.01 to 1.68) | 0.42 | **0.047** |  |  |
| **Sampling** Outpatients | 247/70 (17 651) | [Ref] | [Ref] | [Ref] | [Ref] | [Ref] |
| Community | 10/5 (701) | 0.03 (-0.29 to 0.35) | 0.16 | 0.87 | 1.5610 | 0.67 |
| Mixed | 11/4 (877) | 0.13 (-0.17 to 0.42) | 0.15 | 0.41 |  |  |
| Unclear | 78/15 (3815) | -0.09 (-0.29 to 0.11) | 0.10 | 0.38 |  |  |
| **Sample age** Adults/Elderly | 306/80 (21 193) | [Ref] | [Ref] | [Ref] | [Ref] | [Ref] |
| Children/Adolescents | 40/14 (1851) | -0.08 (-0.32 to 0.16) | 0.12 | 0.50 | 0.4525 | 0.50 |

|  | **o/k (n)** | **Estimated SMD (95%CI)** | **SE** | **p value** | **Test of moderators (QM)** | **p value** |
| --- | --- | --- | --- | --- | --- | --- |
| **Benzodiazepine use**  No | 184/51 (15 040) | [Ref] | [Ref] | [Ref] | [Ref] | [Ref] |
| Yes | 58/11 (1598) | 0.04 (-0.16 to 0.25) | 0.11 | 0.67 | 0.8825 | 0.83 |
| Not informed | 99/30 (5869) | -0.03 (-0.19 to 0.13) | 0.08 | 0.74 |  |  |
| Unclear | 5/2 (537) | 0.28 (-0.47 to 1.02) | 0.38 | 0.46 |  |  |
| **Placebo lead-in** No | 105/35 (6492) | [Ref] | [Ref] | [Ref] | [Ref] | [Ref] |
| Yes | 200/43 (13 336) | 0.03 (-0.15 to 0.22) | 0.09 | 0.72 | 2.2593 | 0.52 |
| Not informed | 34/14 (2666) | -0.13 (-0.37 to 0.10) | 0.12 | 0.27 |  |  |
| Unclear | 7/2 (550) | 0.18 (-0.20 to 0.55) | 0.19 | 0.35 |  |  |
| **Analysis** Mixed/Hierarchical/Random | 22/5 (856) | [Ref] | [Ref] | [Ref] | [Ref] | [Ref] |
| LOCF | 291/78 (21 478) | -0.16 (-0.42 to 0.11) | 0.14 | 0.25 | 3.5848 | 0.31 |
| Completers | 4/2 (65) | 0.29 (-0.39 to 0.97) | 0.35 | 0.40 |  |  |
| Unclear | 29/9 (645) | 0.01 (-0.42 to 0.44) | 0.22 | 0.97 |  |  |

|  | **o/k (n)** | **Estimated SMD (95%CI)** | **SE** | **p value** | **Test of moderators (QM)** | **p value** |  |
| --- | --- | --- | --- | --- | --- | --- | --- |
| **Funding** Academic | 17/8 (415) | [Ref] | [Ref] | [Ref] | [Ref] | [Ref] |  |
| Governmental or non-profit | 21/7 (366) | 0.54 (0.05 to 1.04) | 0.25 | **0.03** | 8.7110 | **0.03** |  |
| Industry | 273/71 (20 460) | 0.61 (0.20 to 1.02) | 0.21 | **0.003** |  |  |  |
| Unclear | 35/8 (1803) | 0.56 (0.10 to 1.02) | 0.23 | **0.01** |  |  |  |
| o, number of outcomes; k, number of studies; n, sample size; SMD, **standardized mean difference;** SE, standard error; QM, Cochran’s Q test of moderators; GAD, generalized anxiety disorder; PTSD, post-traumatic stress disorder; OCD, obsessive-compulsive disorder; LOCF, last observation carried forward; QE, Cochran’s Q test for residual heterogeneity; test of moderators of the multiple meta-regression model [QM]=98.1922, p value<.001; test for residual heterogeneity of the multiple meta-regression model [QE]=454.9043, p value<.001 | | | | | | |  |
