# Supplementary figures and images for: Selective serotonin reuptake inhibitors, and serotonin and norepinephrine reuptake inhibitors for anxiety, obsessive-compulsive, and stress disorders: A 3-level network meta-analysis
Source: PLoS Med. 2021 Jun 10;18(6):e1003664. doi: 10.1371/journal.pmed.1003664 (PMC8224914; doi:10.1371/journal.pmed.1003664)

**S14 Appendix. Risk of bias summary**

**
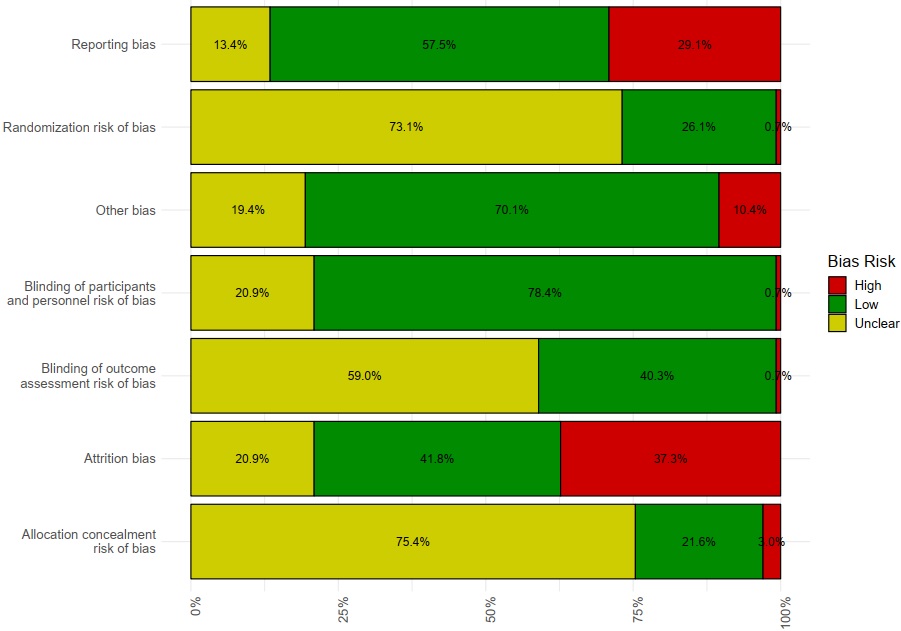
**

Supplement: S14 Appendix — (DOCX) [file pmed.1003664.s014.docx]

**S16 Appendix. Funnel plot for all internalizing symptoms** **
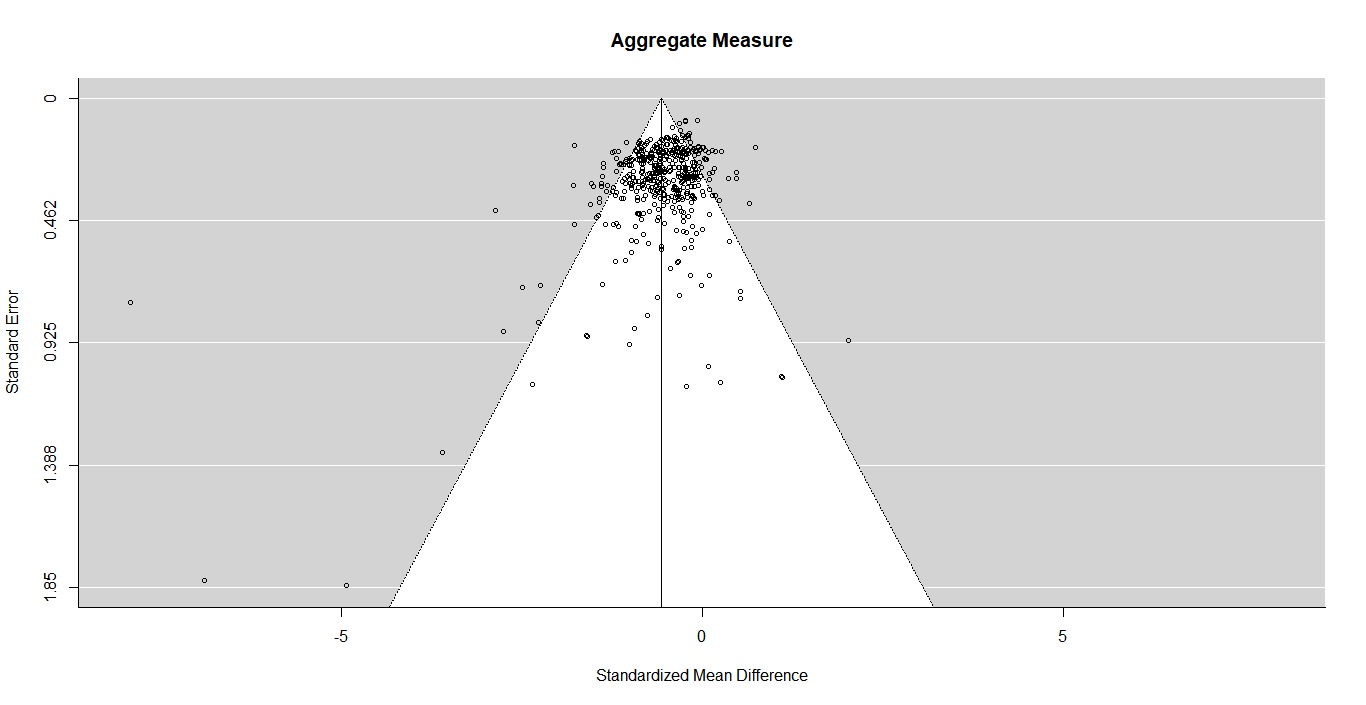
**

Supplement: S16 Appendix — (DOCX) [file pmed.1003664.s016.docx]

**S17 Appendix. Funnel plot for the generalized anxiety disorder domain**

**
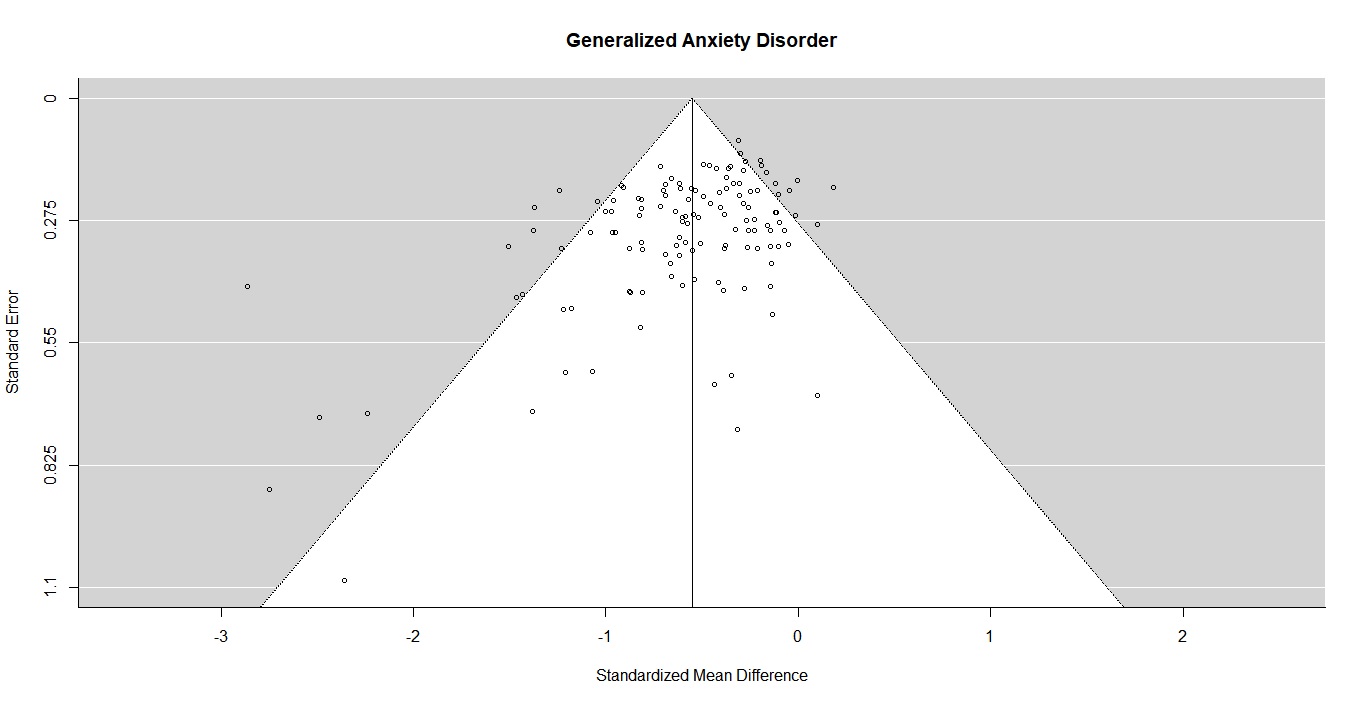
**

Supplement: S17 Appendix — (DOCX) [file pmed.1003664.s017.docx]

**S18 Appendix. Funnel plot for the panic disorder domain**


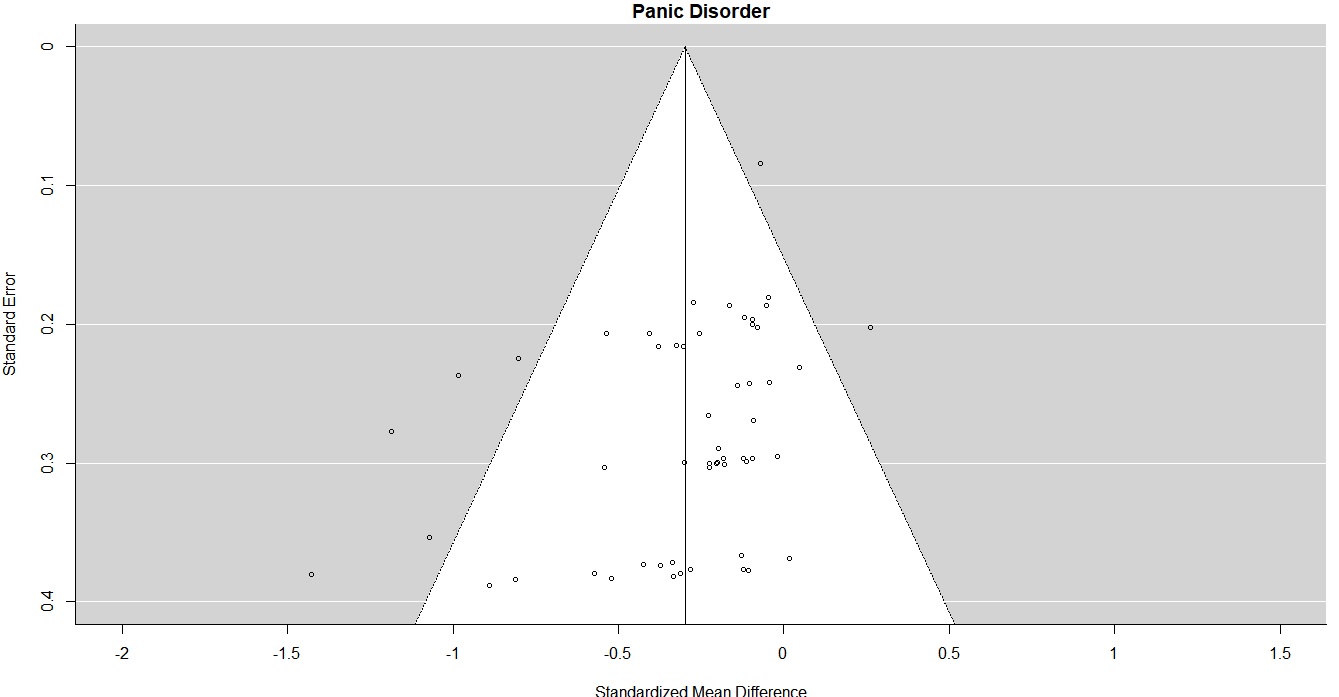

Supplement: S18 Appendix — (DOCX) [file pmed.1003664.s018.docx]

**S19 Appendix. Funnel plot for the social anxiety disorder domain**


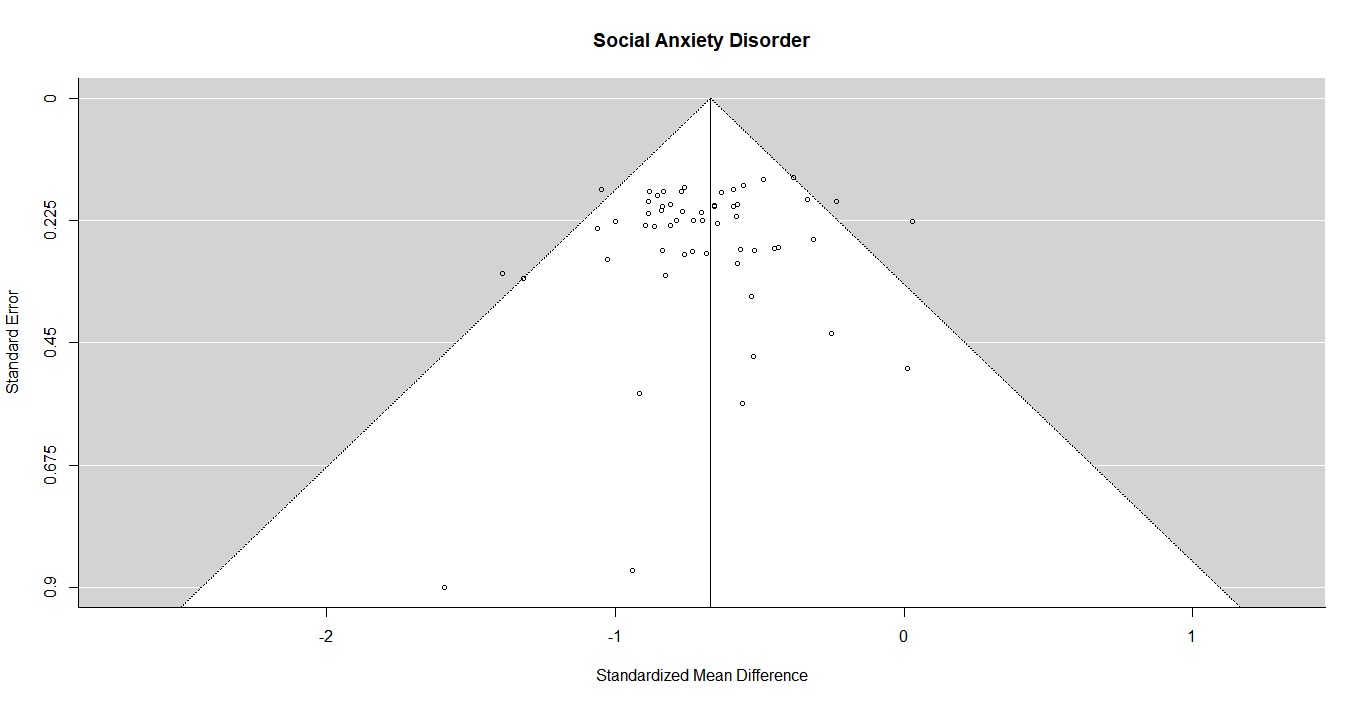

Supplement: S19 Appendix — (DOCX) [file pmed.1003664.s019.docx]

**S20 Appendix. Funnel plot for the specific phobia domain**


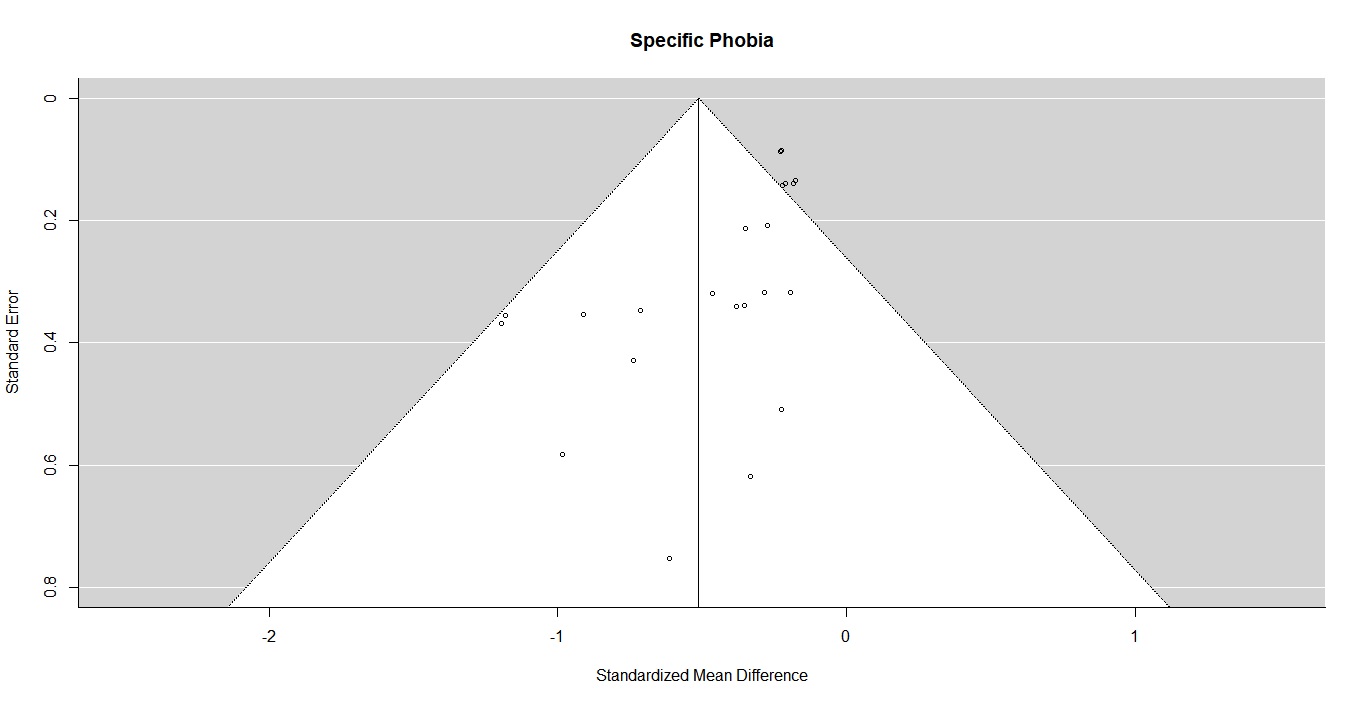

Supplement: S20 Appendix — (DOCX) [file pmed.1003664.s020.docx]

**S21 Appendix. Funnel plot for the obsessive-compulsive disorder domain**

**
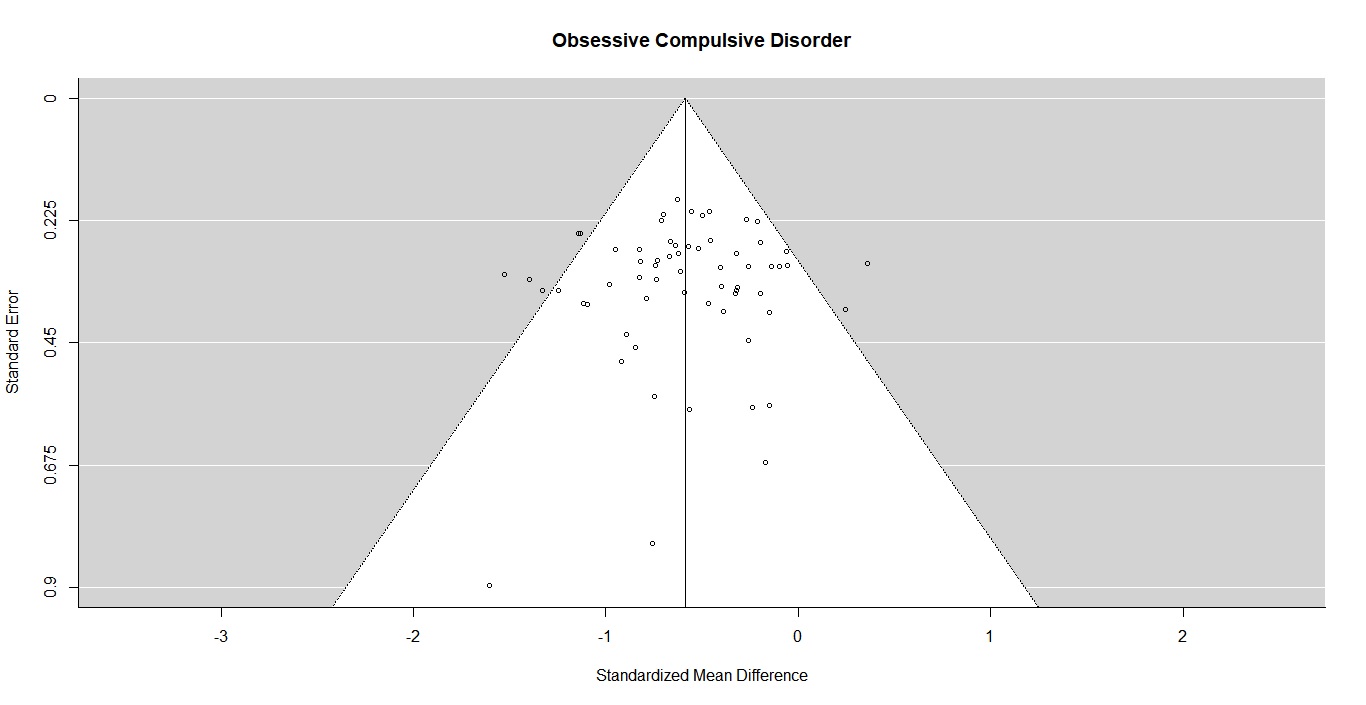
**

Supplement: S21 Appendix — (DOCX) [file pmed.1003664.s021.docx]

**S22 Appendix. Funnel plot for the post-traumatic stress disorder domain**
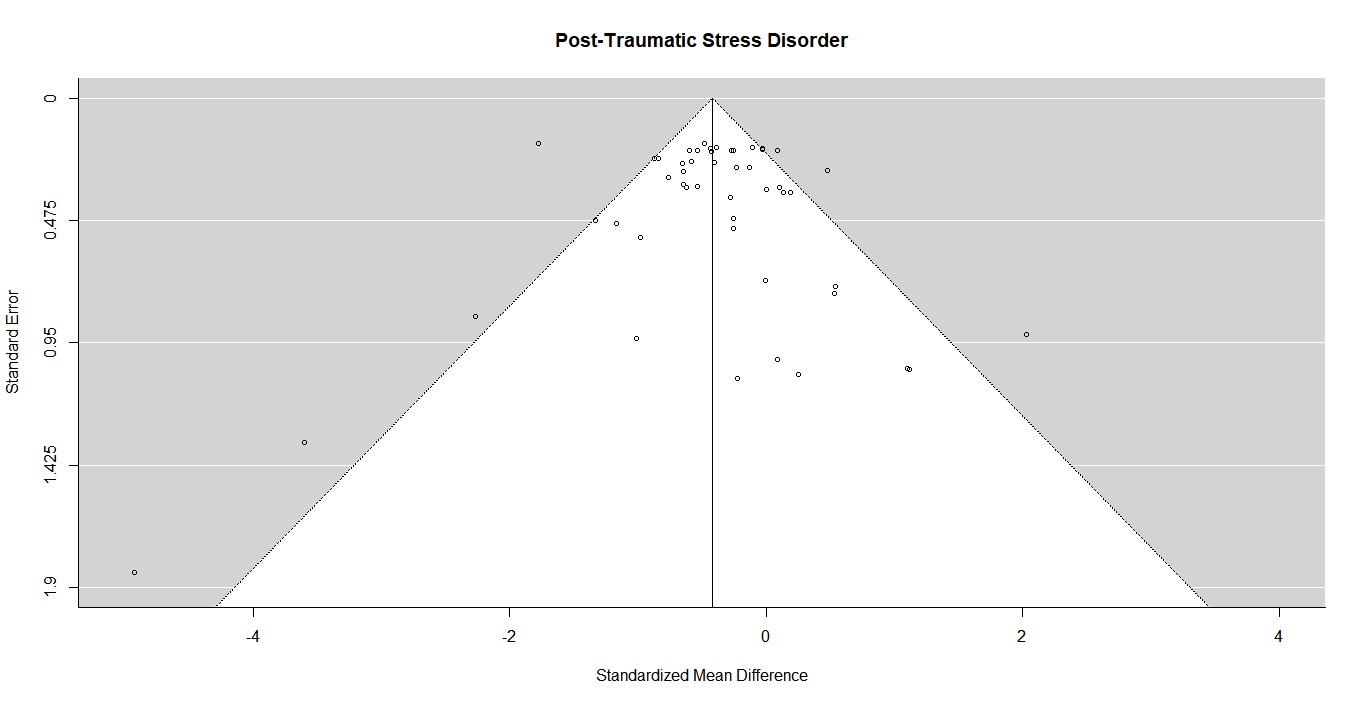

Supplement: S22 Appendix — (DOCX) [file pmed.1003664.s022.docx]
