# Supplementary material for: Selective serotonin reuptake inhibitors, and serotonin and norepinephrine reuptake inhibitors for anxiety, obsessive-compulsive, and stress disorders: A 3-level network meta-analysis
Source: PLoS Med. 2021 Jun 10;18(6):e1003664. doi: 10.1371/journal.pmed.1003664 (PMC8224914; doi:10.1371/journal.pmed.1003664)
Supplement: S15 Appendix — (DOCX) [file pmed.1003664.s015.docx]

**S15 Appendix. Risk of bias in included studies**

| **Id** | **Author** | **Randomization risk of bias** | **Allocation concealment risk of bias** | **Blinding of participants and personnel risk of bias** | **Blinding of outcome assessment risk of bias** | **Attrition risk of bias** | **Reporting risk of bias** | **Other risk of bias** |
| --- | --- | --- | --- | --- | --- | --- | --- | --- |
| JF10 | Allgulander | low | high | low | low | high | unclear | unclear |
| JF11 | Allgulander C. | low | unclear | low | low | low | low | low |
| JF15 | Asakura S. | unclear | unclear | low | unclear | low | low | low |
| JF16 | Asnis | unclear | unclear | low | unclear | low | low | low |
| JF20 | Bakker | unclear | unclear | low | unclear | low | low | low |
| JF22 | Baldwin | low | unclear | low | unclear | low | low | low |
| JF25 | Baldwin D. | unclear | unclear | low | unclear | low | low | low |
| JF28 | Ballenger | unclear | unclear | low | unclear | low | low | low |
| JF29 | Bandelow | low | low | low | low | low | low | low |
| JF3 | Alaka | low | high | low | unclear | unclear | unclear | low |
| JF34 | Beidel D.C. | unclear | low | low | low | unclear | unclear | unclear |
| JF42 | Birmaher | unclear | unclear | low | low | unclear | low | low |
| JF45 | Black | unclear | unclear | low | low | unclear | low | low |
| JF56 | Bradwejn | unclear | low | low | low | unclear | low | low |
| JF59 | Brady | unclear | low | low | low | unclear | low | low |
| JF61 | Brawman-Mintzer O. | low | low | low | low | unclear | low | low |
| JF7 | Allgulander | unclear | unclear | unclear | unclear | low | low | unclear |
| JF72 | Chouinard | unclear | unclear | low | unclear | unclear | low | low |
| JF78 | Connor K.M. | unclear | low | low | unclear | unclear | low | low |
| JF80 | Coric | unclear | unclear | low | unclear | unclear | low | low |
| JF82 | Da Costa | unclear | unclear | low | unclear | unclear | low | low |
| JF83 | Dahl | unclear | unclear | low | unclear | unclear | unclear | low |
| JF87 | Davidson | unclear | unclear | low | unclear | unclear | unclear | low |
| JF88 | Davidson | unclear | unclear | low | unclear | unclear | unclear | low |
| JF89 | Davidson | unclear | unclear | low | unclear | high | low | low |
| JF9 | Koponen | low | unclear | low | unclear | high | high | low |
| JF94 | Asakura S. | low | high | low | unclear | low | low | low |
| LM10 | Den Boer | unclear | unclear | unclear | unclear | low | high | unclear |
| LM23 | Fani N. | unclear | unclear | unclear | high | low | low | low |
| LM24 | Fani N. | unclear | unclear | unclear | high | low | low | low |
| LM34 | Friedman M.J. | unclear | low | low | unclear | low | low | low |
| LM37 | Gelenberg | low | low | unclear | unclear | low | high | low |
| LM39 | Geller D.A. | unclear | unclear | unclear | unclear | low | low | low |
| LM4 | Davidson J. | low | low | unclear | unclear | low | low | unclear |
| LM40 | Geller D.A. | low | low | unclear | unclear | low | high | low |
| LM42 | Gimenez M. | unclear | unclear | unclear | unclear | low | high | high |
| LM48 | Goodman W.K. | unclear | unclear | unclear | unclear | low | high | high |
| LM5 | Davidson J.R.T. | low | low | high | high | low | low | low |
| LM50 | Greist | unclear | unclear | unclear | unclear | low | high | high |
| LM54 | Hartford | unclear | unclear | unclear | unclear | low | low | low |
| LM57 | Hertzberg | unclear | unclear | unclear | unclear | low | unclear | unclear |
| LM59 | Hoehn-Saric | unclear | unclear | unclear | unclear | high | high | high |
| LM6 | Davidson J.R.T. | unclear | low | unclear | unclear | low | high | unclear |
| LM60 | Hollander | unclear | unclear | unclear | unclear | high | low | low |
| LM67 | Jenike | unclear | unclear | unclear | unclear | low | low | low |
| LM69 | Jenike M.A. | unclear | unclear | low | low | high | low | low |
| LM71 | Kamijima K. | unclear | unclear | unclear | unclear | high | high | high |
| LM72 | Kasper | low | unclear | unclear | unclear | low | low | low |
| LM73 | Kasper | unclear | unclear | unclear | unclear | unclear | high | unclear |
| LM74 | Kasper S. | low | low | low | low | low | low | low |
| LM76 | Katzelnick D.J. | unclear | unclear | unclear | unclear | high | high | low |
| LM86 | Koszycki | low | low | low | low | low | low | unclear |
| LM95 | Li | low | low | low | low | low | low | low |
| MC1 | Ledley | unclear | unclear | low | low | high | high | low |
| MC10 | Lepola | unclear | unclear | low | low | high | high | low |
| MC12 | Liebowitz | unclear | unclear | low | low | low | low | low |
| MC13 | Liebowitz | unclear | unclear | low | low | low | low | low |
| MC14 | Liebowitz | unclear | unclear | low | low | low | high | high |
| MC15 | Liebowitz | unclear | unclear | low | low | low | low | high |
| MC16 | Liebowitz | unclear | unclear | low | low | low | low | low |
| MC17 | Liebowitz M.R. | unclear | unclear | low | low | high | high | low |
| MC2 | Leinonen | unclear | unclear | low | low | high | high | low |
| MC20a | Londborg P.D. | low | unclear | low | low | low | high | high |
| MC20b | Londborg P.D. | low | unclear | low | low | low | high | high |
| MC22 | Mahableshwarkar | low | unclear | low | low | high | high | high |
| MC25 | March | low | low | low | low | low | low | low |
| MC26 | March | low | low | low | low | high | low | low |
| MC28 | March J.S. | low | low | low | low | high | low | low |
| MC3 | Lenox-Smith | low | low | low | low | high | high | low |
| MC31 | Marshall | unclear | unclear | low | low | low | low | low |
| MC32 | Marshall | unclear | unclear | low | low | high | low | low |
| MC33 | Martenyi | unclear | unclear | low | low | high | low | low |
| MC34 | Martenyi | unclear | low | low | low | low | low | low |
| MC38 | Merideth | low | unclear | low | low | high | low | low |
| MC39 | Michelson | unclear | unclear | low | low | unclear | high | low |
| MC4 | Lenze | low | low | low | low | high | high | low |
| MC40 | Michelson D. | unclear | unclear | low | low | high | low | low |
| MC42 | Montgomery | unclear | unclear | low | low | low | low | low |
| MC44 | Montgomery | unclear | unclear | low | low | high | low | low |
| MC45 | Montgomery | unclear | unclear | low | low | low | low | low |
| MC51 | Nair | unclear | unclear | low | low | low | high | low |
| MC55 | Nicolini | low | low | low | low | high | high | low |
| MC56 | Nimatoudis I. | unclear | unclear | low | low | low | low | high |
| MC6 | Lenze | low | low | low | low | low | low | low |
| MC62 | Panahi | unclear | low | low | low | high | low | low |
| MC73 | Pollack | unclear | unclear | low | low | high | low | low |
| MC77 | Pollack | unclear | unclear | low | low | low | high | low |
| MC79 | Pollack | unclear | unclear | low | low | high | low | low |
| MC81 | Pollack | unclear | unclear | low | low | high | low | low |
| MC82 | Pollack M.H. | unclear | unclear | low | low | low | low | low |
| MJ1 | Rickels | unclear | unclear | low | unclear | high | high | unclear |
| MJ14 | Rynn | unclear | unclear | low | unclear | high | low | low |
| MJ16 | Rynn M.A. | unclear | unclear | low | unclear | high | low | low |
| MJ17 | Sandmann J. | unclear | unclear | low | unclear | high | high | low |
| MJ2 | Rickels | unclear | unclear | low | low | high | low | low |
| MJ22 | Sharp D.M. | unclear | unclear | low | unclear | low | high | low |
| MJ25 | Sheehan | unclear | unclear | low | unclear | high | low | low |
| MJ3 | Rickels K. | unclear | unclear | low | unclear | low | low | low |
| MJ36 | Stahl | unclear | unclear | low | unclear | low | low | low |
| MJ4 | Riddle | unclear | unclear | low | low | high | high | high |
| MJ42 | Stein | low | unclear | low | unclear | high | high | low |
| MJ44 | Stein | low | low | low | unclear | low | low | low |
| MJ5 | Riddle M.A. | unclear | unclear | low | unclear | high | low | low |
| MJ53 | Stein M.B. | unclear | unclear | low | unclear | low | high | unclear |
| MJ54 | Stein M.B. | low | low | low | unclear | high | low | low |
| MJ56 | Strawn | unclear | low | low | unclear | low | low | low |
| MJ6 | Robb A.S. | unclear | unclear | low | unclear | high | low | low |
| MJ64 | Tucker | unclear | unclear | low | unclear | unclear | low | low |
| MJ66 | Tucker P. | unclear | unclear | low | unclear | low | low | low |
| MJ7 | Rolland P.D. | unclear | unclear | low | unclear | high | high | high |
| MJ70 | Van Ameringen M.A. | unclear | unclear | low | unclear | high | high | low |
| MJ71 | Van Der Kolk | low | unclear | low | unclear | high | high | unclear |
| MJ73 | Van Vliet I.M. | unclear | unclear | low | unclear | high | high | unclear |
| MJ77 | Wade | unclear | unclear | low | unclear | low | high | unclear |
| MJ78 | Wagner | low | unclear | low | unclear | high | low | low |
| MJ79 | Walkup | low | low | low | unclear | high | low | high |
| MJ80 | Walkup | unclear | unclear | low | unclear | high | high | unclear |
| MJ84 | Westenberg | unclear | unclear | low | unclear | high | low | low |
| MJ85 | Westenberg | unclear | unclear | low | unclear | high | high | unclear |
| MJ89 | Wu | unclear | unclear | low | unclear | high | low | low |
| MJ93 | Zohar | unclear | unclear | low | unclear | low | low | low |
| MJ94 | Zohar | unclear | unclear | low | unclear | high | low | low |
| MJ96 | Stein D.J. | low | low | low | unclear | low | high | low |
| MJ97 | Jenike | high | high | low | unclear | high | low | high |
| UNG1 | unknown | unclear | unclear | low | unclear | unclear | unclear | low |
| UNG10 | unknown | unclear | unclear | unclear | unclear | unclear | unclear | unclear |
| UNG11 | unknown | unclear | unclear | unclear | unclear | unclear | unclear | unclear |
| UNG12 | unknown | unclear | unclear | unclear | unclear | unclear | unclear | unclear |
| UNG17 | unknown | unclear | unclear | unclear | unclear | unclear | unclear | unclear |
| UNG2 | unknown | unclear | unclear | low | unclear | unclear | unclear | low |
| UNG3 | unknown | unclear | unclear | unclear | unclear | unclear | unclear | unclear |
| UNG6 | Hewett | unclear | unclear | unclear | unclear | unclear | unclear | unclear |
| UNG7 | unknown | unclear | unclear | unclear | unclear | unclear | unclear | unclear |
| UNG8 | Sonne | low | unclear | unclear | unclear | unclear | unclear | unclear |
| UNG9 | unknown | unclear | unclear | unclear | unclear | unclear | unclear | unclear |
| UPD3 | Liebowitz MR | unclear | unclear | low | low | low | low | unclear |
| UPD8 | Strawn JR | low | low | low | low | low | low | low |
